# Crystallography of organic energetic materials: thermoelastic properties and equation of state

**DOI:** 10.1107/S2052520626008322

**Published:** 2026-09-15

**Authors:** Mathieu Guerain

**Affiliations:** a Université de Lille CNRS, INRA, ENSCL, UMR 8207 – UMET–Unité Matériaux et Transformations F-59650 Villeneuve d’Ascq France; Polish Academy of Sciences, Poland

**Keywords:** energetic materials, crystallography, equation of state, temperature experiments, high pressure

## Abstract

Energetic molecular crystals exhibit complex behaviour under extreme conditions of temperature and pressure, which critically influences their performance and safety. However, thermoelastic data remain scattered throughout the literature and are rarely analysed within a unified comparative framework, particularly across multiple classes of energetic materials. In this work, crystallographic data for 16 energetic materials were compiled and systematically analysed to investigate their thermal expansion and equation of state parameters. Thermal expansion coefficients and equation of state parameters were extracted or re-evaluated from experimental variable-temperature and high-pressure diffraction-based crystallographic studies. While volumetric thermal expansion is found to be relatively similar across materials, significant differences in anisotropy are observed, strongly governed by molecular packing and polymorphism. High-pressure data show that most materials exhibit bulk moduli in the range 10–20 GPa, while crystal density appears to influence compressibility, although considerable dispersion exists among materials. These results highlight the central role of crystal structure in controlling thermoelastic behaviour and provide a unified framework linking crystal structure, anisotropy and thermoelastic response in energetic molecular crystals for modelling energetic materials under extreme conditions.

## Abstract 2

Thermoelastic properties and equation-of-state parameters of 16 organic energetic molecular crystals are systematically compiled and compared from temperature- and pressure-dependent crystallographic data. The analysis highlights anisotropic thermal expansion and compressibility and identifies structural factors governing the mechanical response of these molecular crystals.

## 1. Introduction

Energetic materials are compounds containing large amounts of stored chemical energy that can be rapidly released upon external stimulation. Their performance depends on several factors that include crystal density, detonation velocity and sensitivity to detonation by external stimuli (Akhavan, 2022 ▸; Meyer *et al.*, 2007 ▸). These macroscopic properties are intrinsically governed by the solid-state organization of the material, making crystallography a central tool for understanding and predicting their behaviour.

In molecular energetic crystals, the arrangement of molecules within the lattice controls intermolecular interactions, packing efficiency and structural anisotropy. As a result, detailed crystallographic information is essential to describe not only structural diversity but also the physical properties that emerge from it. Building upon this structural framework, the present study investigates their thermoelastic properties and equation of state parameters under varying temperature and pressure conditions.

Energetic materials detonate under extreme conditions, typically in the high-pressure (10–50 GPa) and high-temperature (873–4273 K) regime. Under these conditions, the crystal structure evolves significantly, and the material transitions from a condensed molecular solid to high-temperature and high-pressure gaseous products. These transformations involve a complex interplay between structural, mechanical and chemical processes, all of which are strongly influenced by the initial crystallographic arrangement.

Modelling such rapid and complex phenomena remains highly challenging but is essential for the safe design and use of energetic materials. Hydro­dynamic simulations used to predict explosive performance rely on an equation of state relating density, temperature, pressure and internal energy. Consequently, accurate determination of thermoelastic parameters, such as compressibility and thermal expansion coefficients (TECs), is essential for improving predictive models of detonation behaviour (Millar, Maynard-Casely *et al.*, 2010 ▸; Millar, Marshall *et al.*, 2010 ▸).

In this context, crystallographic investigations under variable temperature and high-pressure conditions provide direct insight into the evolution of unit-cell parameters, unit-cell volumes and structural anisotropy. These data form the basis for determining thermoelastic properties and for establishing equations of state that describe the pressure–volume–temperature relationships of energetic molecular crystals. However, such data remain scattered in the literature and are rarely analysed within a unified crystallographic framework.

Thermal expansion of energetic materials plays a key role as these compounds generally expand with increasing temperature (Maienschein & Garcia, 2002 ▸). In molecular crystals, this expansion may be anisotropic and strongly dependent on the crystallographic directions. Such anisotropy can significantly affect density, mechanical behaviour and structural stability. Moreover, temperature-induced phase transitions may generate internal stresses, defects or microcracks, which can act as hot spots and increase sensitivity. Accurate determination of TECs is therefore essential for evaluating the thermal stability and safety of energetic materials (Maienschein & Garcia, 2002 ▸; US Dept of Energy, 2023 ▸).

Similarly, the response of energetic materials to high-pressure is of primary importance. A detailed understanding of the high-pressure response of crystal structures and molecular bonding is therefore essential for elucidating the mechanisms of detonation initiation and the stability of energetic crystals (Aslam, 2018 ▸; Manaa *et al.*, 2014 ▸). The pressure–volume relationship of molecular crystals is commonly described using analytical equations of state, among which the Birch–Murnaghan formalism is one of the most widely used (Birch, 1947 ▸). High-pressure experiments provide access to the compressibility and evolution of unit-cell parameters and unit-cell volumes under compression, typically through X-ray diffraction measurements performed in diamond anvil cells (DACs) (Boldyreva, 2008 ▸; Oswald *et al.*, 2010 ▸). These experiments also enable the detection of pressure-induced phase transitions, which may significantly influence stability and detonation behaviour.

Despite the importance of thermoelastic parameters, crystallographic data describing the equation of state of energetic molecular crystals remain scattered throughout the literature, and no systematic comparative analysis of thermoelastic properties across multiple energetic molecular crystals has been performed to date. The present study provides a systematic comparison of thermoelastic properties for 16 energetic materials. Reported crystallographic data are compiled and analysed to examine the influence of temperature and pressure on their crystal structures through the evolution of unit-cell parameters and unit-cell volumes. Thermoelastic properties, including TECs and compressibility, are collected or determined when possible. These data are then used to compare the parameters of the Birch–Murnaghan equation of state and to identify general trends in the thermoelastic behaviour of energetic molecular crystals.

## 2. High-pressure and variable-temperature experiments

### 2.1. Tools

To determine the thermoelastic coefficients and the pressure dependence of the unit-cell volume, crystallographic analysis must be performed under controlled temperature and pressure conditions. In this work, only diffraction-based crystallographic studies (neutron or X-ray) were considered, as those techniques provide direct access to unit-cell parameters, unit-cell volumes and their evolution as a function of temperature and pressure. The main experimental approaches used in the literature are briefly outlined below.

For variable-temperature experiments, the sample is placed in a temperature-controlled stage mounted on the diffracto­meter, allowing *in situ* collection of X-ray diffraction patterns over a wide temperature range. Most experiments are performed using laboratory X-ray sources, either on single crystals [RDX (Bolotina & Pinkerton, 2015 ▸), TNT (Vrcelj *et al.*, 2003 ▸), HMX (Bolotina & Pinkerton, 2015 ▸; Deschamps *et al.*, 2011 ▸; Yan *et al.*, 2023 ▸), PETN (Bolotina & Pinkerton, 2015 ▸), FOX-7 (Bolotina & Pinkerton, 2015 ▸; Evers *et al.*, 2006 ▸; Crawford *et al.*, 2007 ▸), CL-20 (Bolotina & Pinkerton, 2015 ▸; Bolotina *et al.*, 2004 ▸), NTO (Wang *et al.*, 2025 ▸), DNAN (Takahashi & Tamura, 2015 ▸)] or a powder sample [RDX (Sun *et al.*, 2011 ▸), FOX-7 (Evers *et al.*, 2006 ▸; Crawford *et al.*, 2007 ▸), CL-20 (Liang *et al.*, 2022 ▸; Pu *et al.*, 2016 ▸), TATB (Sun *et al.*, 2010 ▸; Kolb & Rizzo, 1979 ▸), LLM-105 (Xu *et al.*, 2019 ▸; Li *et al.*, 2016 ▸), HNS (Shu *et al.*, 2011 ▸), DNAN (Stankevich *et al.*, 2024 ▸)]. For higher accuracy, synchrotron radiation is sometimes used for both single-crystal [FOX-7 (McMonagle *et al.*, 2022 ▸)] and powder diffraction experiments [FOX-7 (Zhang *et al.*, 2016 ▸), LLM-105 (Gump *et al.*, 2011 ▸), HNS (Gump *et al.*, 2012 ▸)], providing improved resolution and signal-to-noise ratios.

High-pressure experiments are most commonly performed using DACs, such as the Merrill–Bassett design (Merrill & Bassett, 1974 ▸). Both powder [TNT (Stevens *et al.*, 2008*a* ▸), HMX (Gao *et al.*, 2019 ▸; Yoo & Cynn, 1999 ▸), FOX-7 (Zhang *et al.*, 2016 ▸), CL-20 (Gump & Peiris, 2008 ▸; Konar *et al.*, 2020 ▸), TATB (Sun *et al.*, 2018 ▸; Steele *et al.*, 2020 ▸; Steele *et al.*, 2019 ▸; Stevens *et al.*, 2008*b* ▸), LLM-105 (Xu *et al.*, 2019 ▸; Gump *et al.*, 2011 ▸; Stavrou *et al.*, 2015 ▸), HNS (Gump *et al.*, 2007 ▸), DAAF (Chellappa *et al.*, 2014 ▸), DNAN (Rajan *et al.*, 2022 ▸)] and single-crystal [RDX (Oswald *et al.*, 2010 ▸; Millar, Oswald *et al.*, 2010 ▸), TNT (Bowden *et al.*, 2014 ▸), HMX (Sui *et al.*, 2019 ▸), FOX-7 (Dreger *et al.*, 2016 ▸), TATB (Plisson *et al.*, 2017 ▸)] experiments have been reported.

Regardless of the experimental conditions, the extraction of crystallographic parameters from diffraction data requires appropriate refinement procedures. In most cases, unit-cell parameters and unit-cell volumes are determined using the Rietveld refinement method. This approach consists of fitting a calculated diffraction pattern to the experimental data by refining structural and instrumental parameters. Starting from a reference structure, the refinement minimizes the difference between observed and calculated intensities, typically through the weighted profile *R*-factor (*R*_wp_), defined by equation (1):
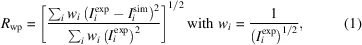
where 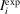 is the intensity of the experimental diffraction pattern at point *i* and 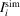 is the intensity of the simulated diffraction pattern at the same point (Young, 1993 ▸). The peak positions in the experimental diffraction pattern depend directly on unit-cell parameters and angles.

The positions of diffraction peaks are directly related to the unit-cell parameters and unit-cell geometry, allowing accurate determination of their evolution with temperature and pressure. Reference structures used as starting models are generally obtained from the literature and are commonly available in crystallographic databases such as the Cambridge Structural Database (CSD) (Groom *et al.*, 2016 ▸).

### 2.2. Data processing and fitting methods

A systematic literature survey was conducted for the 16 energetic molecular crystals considered in this review in order to identify variable-temperature and variable-pressure diffraction studies reporting unit-cell parameters and/or unit-cell volumes. These materials include RDX, TNT, HMX, PETN, FOX-7, CL-20, TATB, LLM-105, HNS, TNP, DAAF, NTO, DATB, TNB, BTF and DNAN. To facilitate the identification of the investigated materials, Table S1 summarizes the chemical formula, full name, abbreviation and molecular structure of each compound.

For each study, crystallographic data obtained from diffraction-based techniques were preferentially collected, including both X-ray diffraction and neutron diffraction investigations where available. The objective of this review was to compile thermoelastic and high-pressure information derived from crystallographic determination of unit-cell parameters and unit-cell volumes. Consequently, non-crystallographic measurements of thermal expansion or compressibility, such as dilatometric studies, were not considered in the quantitative analysis. Neutron diffraction provides valuable complementary information, particularly for hydrogen-bonded energetic materials, owing to its improved sensitivity to hydrogen-atom positions and reduced absorption effects compared with X-ray diffraction.

Numerical crystallographic data were preferentially extracted from the original publications. When numerical values were not reported in the main article, the associated supplementary information was examined. Additional datasets were also searched in related doctoral theses where available. Graphical digitization of published figures was only performed as a last resort when no tabulated numerical values could be located in any available source. The method used to obtain the data is described material by material in Tables S2 (analyses under temperature) and S3 (analyses under pressure).

Polymorphic phases were treated independently throughout the analysis. Each crystalline phase was considered as a distinct dataset and thermoelastic parameters were determined separately for each polymorph. In several cases, structural phase transitions have been reported under temperature or pressure variations, leading to the existence of multiple polymorphs. These transformations must be carefully considered, as they may significantly affect the determination and interpretation of thermoelastic parameters. Furthermore, when phase transitions were reported in the original studies, the corresponding transition pressures or temperatures were explicitly indicated in the tables and figures. Data belonging to different crystallographic phases were not combined within a single fit.

TECs were compiled for all materials and polymorphs considered in this work. When linear or volumetric TECs were directly reported by the original authors, these values were adopted without modification and referenced accordingly. When such coefficients were not available, they were recalculated from the reported unit-cell parameter or unit-cell volume data using linear regression over the investigated temperature range. The origin of each TEC (literature value or recalculated in the present work) is specified in supporting information (Table S2).

Equation of state parameters were compiled from available high-pressure diffraction studies. To facilitate comparison between materials and polymorphs, the parameters reported correspond to the third-order Birch–Murnaghan equation of state [equation (2)], namely the zero-pressure unit-cell volume (*V*_0_), the bulk modulus (*B*_0_) and its first pressure derivative (*B*′). In this equation, *V* is unit-cell volume and *P* is pressure. In the vast majority of cases, these parameters were directly taken from the original publications. For two datasets [PETN (Olinger *et al.*, 1975 ▸) and TATB (Plisson *et al.*, 2017 ▸)], where Birch–Murnaghan parameters were not reported, *B*_0_ and *B*′ were recalculated from the available pressure–volume data using this third-order Birch–Murnaghan fit:
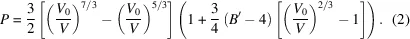


Whenever uncertainties were reported in the original publications, these values are retained in the present compilation. In cases where uncertainty estimates were not provided by the original authors, the notation ‘n.r.’ (not reported) was used. A detailed summary of the provenance of all datasets, including the source of the numerical values and the data-processing methodology, is provided in Table S3. Weighted regressions and reduced χ^2^ values were not considered because experimental uncertainties associated with the original unit-cell parameters were not systematically reported and heterogeneous datasets originating from different experimental techniques were sometimes combined. Consequently, recalculated uncertainties only reflect the statistical quality of the linear regression and should not be interpreted as full experimental uncertainties.

High-pressure crystallographic studies of energetic materials are predominantly performed with DACs using a variety of pressure-transmitting media. Because the apparent compressibility and elastic anisotropy may depend on the degree of hydro­staticity achieved during compression, the experimental conditions associated with each dataset are summarized in Table S3, including the diffraction technique employed (single-crystal or powder diffraction, X-ray or neutron diffraction) and the pressure-transmitting medium used. In principle, pressure-transmitting media may lose hydro­staticity above characteristic pressure limits, potentially generating deviatoric stresses that affect lattice strains, axial compressibility, bulk moduli (*B*_0_) and their pressure derivatives (*B*′). However, several studies included in this review report low deviatoric stress values even when the nominal hydro­static limit of the pressure-transmitting medium is exceeded (Stavrou *et al.*, 2015 ▸; Plisson *et al.*, 2017 ▸), suggesting that quasi-hydro­static conditions may still be maintained over part of the investigated pressure range. Consequently, differences observed between literature datasets may reflect both intrinsic material behaviour and variations in experimental conditions. The corresponding equation of state parameters should therefore be interpreted within the context of the experimental methodology used in each study.

For each compound, available studies were analysed in order to extract unit-cell parameters, unit-cell volumes and their evolution as a function of temperature and pressure. The results are summarized in supporting information (Tables S4–S39). Where possible, data from multiple sources were combined to extend the accessible pressure or temperature ranges and improve the consistency of the derived thermoelastic parameters. An example of such analysis is provided for the RDX.

### 2.3. Energetic materials investigated

**Figure 1:**
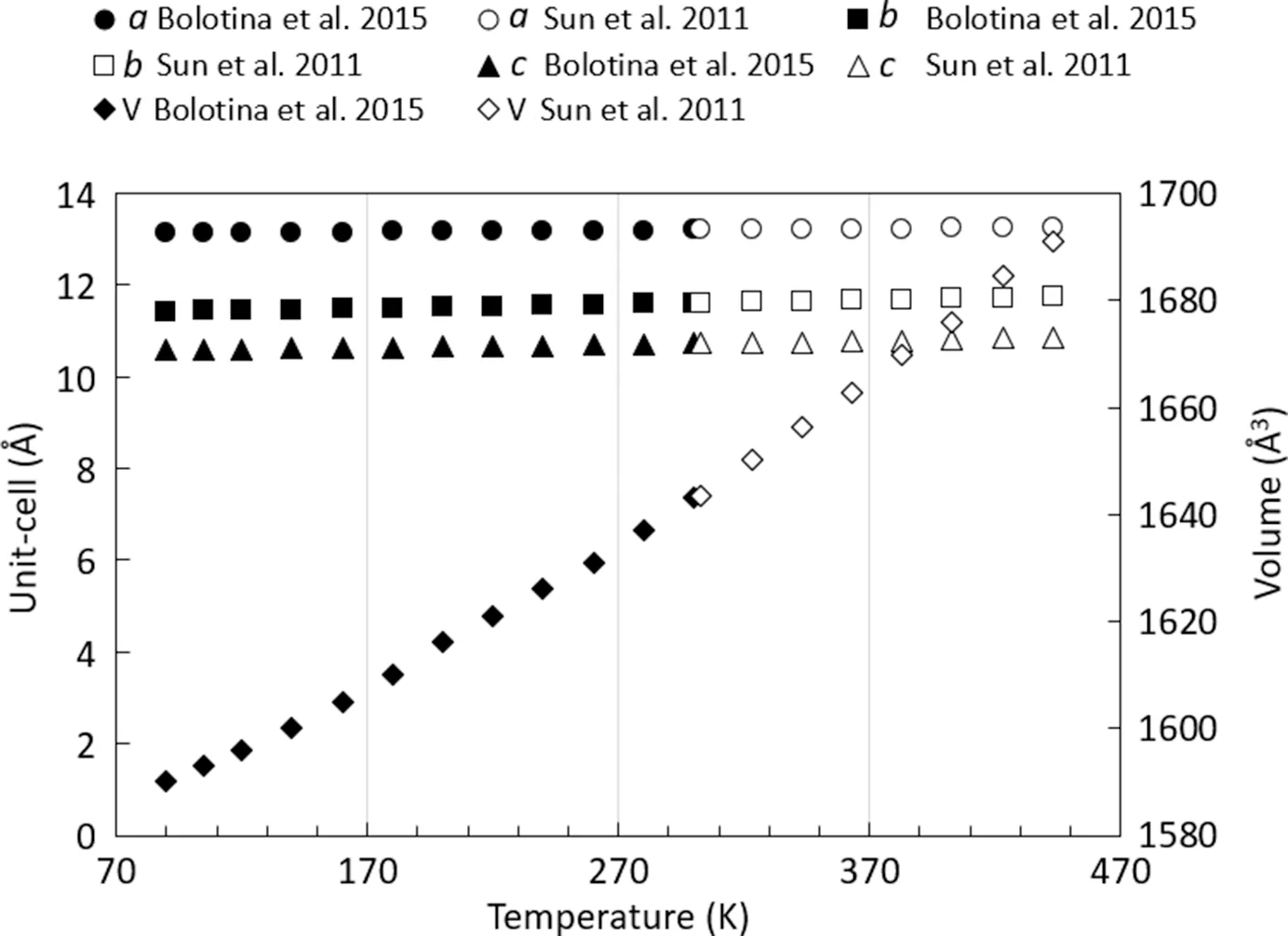
Evolution of *a*, *b* and *c* axes and volume of RDX during the heating process.

The thermal behaviour of α-RDX has been studied by Bolotina & Pinkerton (2015 ▸) at low temperatures (90–300 K) and Sun *et al.* (2011 ▸) at high temperatures (303–443 K), providing a continuous description of unit-cell parameters and unit-cell volume over a temperature range in excess of 350 K (Fig. 1 ▸). High-pressure experiments performed up to 4 GPa show a decrease of approximately 15% in unit-cell volume for α-RDX (Oswald *et al.*, 2010 ▸). A polymorphic transition to γ-RDX is reported between 4 and 8.1 GPa. At 8 GPa, the volume of γ-RDX is about 75% of that of α-RDX at ambient pressure. Recrystallization experiments at 5.7 GPa led to the stabilization of the ɛ-RDX form (Millar, Oswald *et al.*, 2010 ▸). For this polymorph, a volume reduction of about 12% is observed between 1 and 5 GPa (Fig. S1). The ɛ form is denser than α-RDX over this pressure range, suggesting a higher detonation performance. Between 4 and 5 GPa, ɛ-RDX and γ-RDX exhibit comparable densities.

Thermal expansion of TNT has been investigated for both monoclinic and orthorhombic polymorphs over the temperature range 123–295 K (Vrcelj *et al.*, 2003 ▸). In both polymorphs, anisotropic thermal expansion is observed. High-pressure studies on monoclinic TNT show a decrease in unit-cell volume of approximately 25% at 10 GPa and 30% at 20 GPa (Fig. S2) (Stevens *et al.*, 2008*a* ▸; Bowden *et al.*, 2014 ▸). Compression is anisotropic, with the *c* axis being more compressible. A significant discontinuity in volume is observed between 20 and 26 GPa, which is attributed to a phase transition from the monoclinic to the orthorhombic form (Stevens *et al.*, 2008*a* ▸).

Thermal behaviour of β-HMX has been studied over temperature ranges extending from 85 K to 423 K (Bolotina & Pinkerton, 2015 ▸; Deschamps *et al.*, 2011 ▸; Yan *et al.*, 2023 ▸). These studies indicate a gradual increase of about 4% in unit-cell volume between 85 K and 373 K, where the β→α phase transition occurs (Fig. S3). High-pressure behaviour has been investigated up to ∼42 GPa (Fig. S4) (Gao *et al.*, 2019 ▸; Yoo & Cynn, 1999 ▸; Sui *et al.*, 2019 ▸). Up to 10 GPa, all studies report similar results, with a volume reduction of about 20%. At higher pressures, discrepancies appear. Gao *et al.* (2019 ▸) suggest a phase transition to a new η phase at ∼16 GPa. In contrast, Sui *et al.* (2019 ▸) and Yoo & Cynn (1999 ▸) observed changes in Raman spectra but concluded from X-ray diffraction that the structure remains in form β. Overall, a volume reduction of 36% at 40 GPa (Sui *et al.*, 2019 ▸) and 40% at 42.6 GPa (Yoo & Cynn, 1999 ▸) is reported. Compression is initially isotropic up to ∼6 GPa (≃ 1% GPa^−1^), then becomes anisotropic between 6 and 14 GPa. Above 14 GPa, a decrease in *a* and *b* parameters is observed, while the *c* parameter increases (Yoo & Cynn, 1999 ▸).

Thermal expansion of PETN (form I) indicates a relatively isotropic expansion, with an overall increase in unit-cell volume of about 7% between 10 K and 373 K (Fig. S5) (Bolotina & Pinkerton, 2015 ▸; Stankevich *et al.*, 2019 ▸). High-pressure experiments up to 10.45 GPa show a decrease of approximately 9% along the *a* axis and 10% along the *c* axis at 10 GPa, resulting in an overall volume reduction of ∼25% (Olinger *et al.*, 1975 ▸). No crystallographic data are currently available for the II-PETN polymorph, either for thermal expansion or for equation of state determination.

FOX-7 is one of the most extensively studied energetic materials in terms of thermal expansion, with data available over a wide temperature range from 80 K to 493 K (Fig. S6). At low temperatures, a lattice expansion of about 3% for α-FOX-7 between 80 K and 295 K is shown (Evers *et al.*, 2006 ▸; Aree *et al.*, 2022 ▸). Between 295 K and 373 K, an additional expansion of approximately 2% is reported (Evers *et al.*, 2006 ▸; McMonagle *et al.*, 2022 ▸). A phase transition to the β form is observed around 373 K, followed by a transition to γ-FOX-7 at around 443 K (McMonagle *et al.*, 2022 ▸). The γ form has been further studied between 200 and 493 K and indicate an overall volume expansion of about 7% between 295 K and 493 K (Crawford *et al.*, 2007 ▸; McMonagle *et al.*, 2022 ▸). Under moderate pressure (∼1 GPa), Zhang *et al.* (2016 ▸) report a reduction in unit-cell volume, indicating an increase in density. Thermal expansion is also reduced under pressure, with only a 3% volume increase observed between 295 K and 485 K. The high-pressure behaviour of α-FOX-7 has been investigated by several authors (Zhang *et al.*, 2016 ▸; Dreger *et al.*, 2016 ▸; Peiris *et al.*, 2004 ▸; Hunter *et al.*, 2015 ▸), showing consistent results (Fig. S7). Between 0 and 4 GPa, the *a* and *c* parameters decrease by about 3%, while the *b* parameter decreases by approximately 9%, indicating stronger compressibility along this direction. The overall volume reduction is about 15%. A polymorphic transition from α to ɛ is reported at ∼5 GPa by Dreger *et al.* (2016 ▸). The ɛ form exhibits a further volume decrease of about 10% between 6 and 12.8 GPa.

Thermal expansion of CL-20 has been investigated for several polymorphs. In particular, the stable ɛ form has been studied over a wide temperature range from 100 K to 403 K using combined datasets (Fig. S8) (Bolotina & Pinkerton, 2015 ▸; Liang *et al.*, 2022 ▸; Pu *et al.*, 2016 ▸). Such works indicate that ɛ-CL-20 exhibits relatively isotropic thermal expansion. For γ-CL-20, the *b* parameter decreases with increasing temperature, while the *a* and *c* parameters increase, highlighting anisotropic thermal expansion (Bolotina & Pinkerton, 2015 ▸; Pu *et al.*, 2016 ▸). In α-CL-20, expansion mainly occurs along the *b* and *c* directions, whereas in β-CL-20 it occurs along the *a* and *c* axes (Pu *et al.*, 2016 ▸). High-pressure behaviour has only been reported for ɛ-CL-20. The work of Gump & Peiris (2008 ▸) shows quasi-isotropic compression between 0 and 5.6 GPa at 295 K, with reductions of approximately 5% (*a*), 7% (*b*) and 5.5% (*c*), corresponding to a 15% decrease in volume. Similar results are obtained at 348 K. These observations are consistent with the results of Konar *et al.* (2020 ▸), who report a volume reduction of about 17% at 5.2 GPa and nearly 20% at 7.2 GPa (Fig. S9).

**Figure 2:**
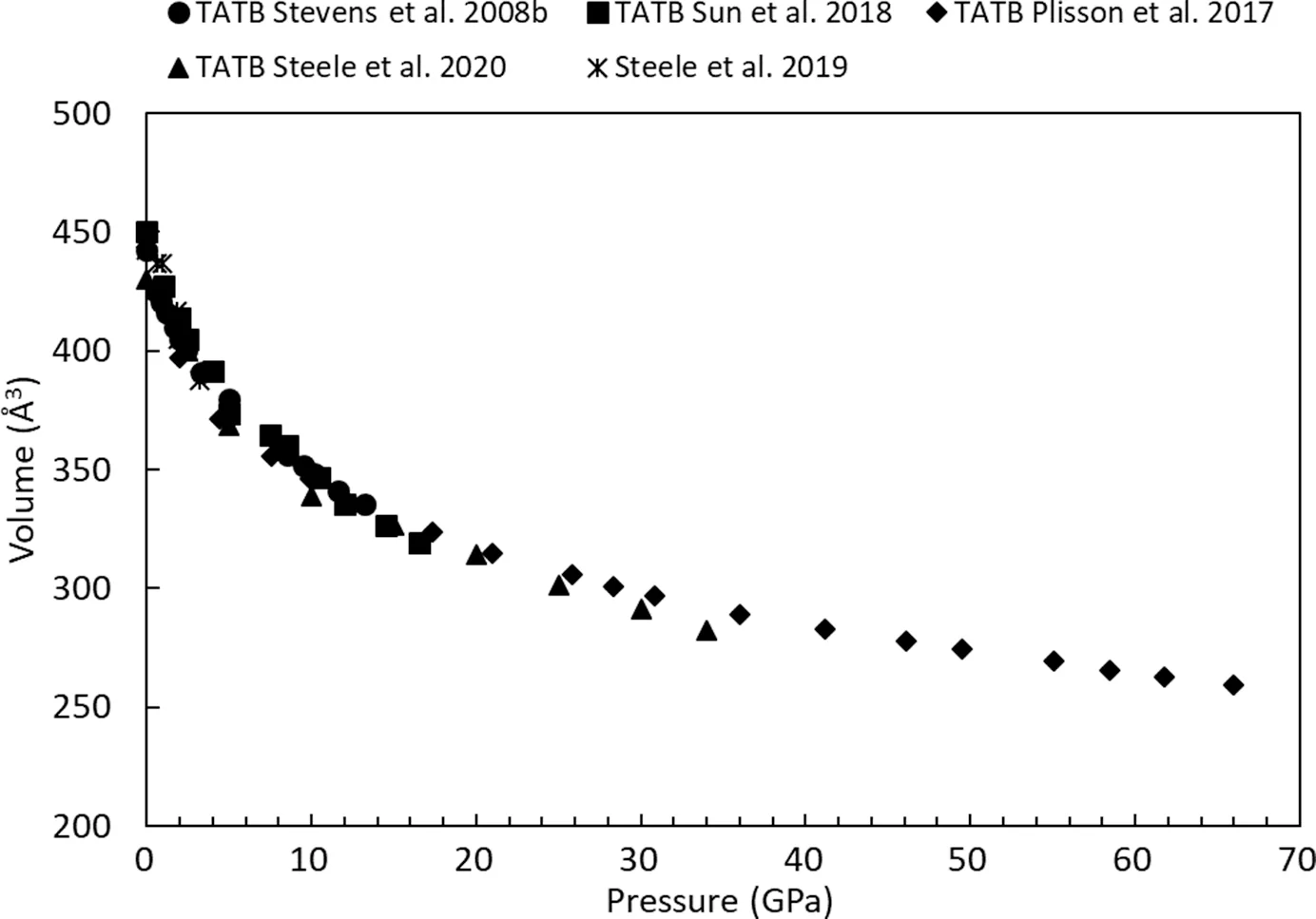
Variation of unit-cell volume of TATB with pressure.

Thermal expansion of TATB has been studied by Kolb & Rizzo (1979 ▸) and Sun *et al.* (2010 ▸) over the temperature range 213 K to 513 K (Fig. S10). Both studies show anisotropic expansion, with significantly larger expansion along the *c* axis compared to the *a* and *b* directions. Kolb & Rizzo (1979 ▸) report a change in slope around 323 K for the *c* parameter and unit-cell volume, whereas Sun *et al.* (2010 ▸) observe a more linear behaviour over the entire temperature range. Despite these differences, both datasets lead to comparable TECs when considering overlapping temperature ranges. This anisotropy is directly related to the layered crystal structure of TATB, where molecules form hydrogen-bonded networks in the *ab* plane and are stacked along the *c* axis through weaker van der Waals interactions. As a result, expansion is favoured along the stacking *c* direction (Sun *et al.*, 2010 ▸; Kolb & Rizzo, 1979 ▸). High-pressure studies performed up to 66 GPa show consistent results among different authors (Sun *et al.*, 2018 ▸; Steele *et al.*, 2020 ▸; Stevens *et al.*, 2008*b* ▸; Plisson *et al.*, 2017 ▸) (Fig. 2 ▸). The unit-cell volume decreases by approximately 20% at 7 GPa, 25% at ∼13 GPa, 33% at 30 GPa and 42% at 66 GPa. Compression is strongly anisotropic and occurs preferentially along the *c* axis, again reflecting the layered structure. A possible triclinic-to-monoclinic transition at ∼4 GPa has been proposed by some authors (Steele *et al.*, 2020 ▸; Steele *et al.*, 2019 ▸) but remains debated. The strong anisotropy of TATB has important implications for its mechanical behaviour, as it may lead to preferential defect formation in the *ab* plane during processing (Guerain *et al.*, 2016 ▸; Mathew & Sewell, 2015 ▸).

Thermal behaviour of LLM-105 has been investigated over a very wide temperature range from 5 to 513 K (Fig. S11) (Xu *et al.*, 2019 ▸; Li *et al.*, 2016 ▸; Gump *et al.*, 2011 ▸). Although some dispersion is observed between datasets, all studies show consistent trends. The unit-cell volume increases from approximately 730 Å^3^ to 778 Å^3^ over the full temperature range, corresponding to an expansion of ∼5.4%. The expansion is anisotropic and occurs predominantly along the *b* axis. High-pressure behaviour has been studied up to 25.8 GPa (Fig. S12) and an overall volume reduction of about 30% is observed (Xu *et al.*, 2019 ▸; Gump *et al.*, 2011 ▸; Stavrou *et al.*, 2015 ▸). Compression is anisotropic and is more pronounced along the *b* axis, consistent with the thermal expansion behaviour. Additional experiments performed by Gump *et al.* (2011 ▸) at 373 K and 453 K show that compressibility follows similar trends at elevated temperatures, indicating a relatively stable mechanical response.

Thermal behaviour of HNS has been studied at high temperature (393–563 K, see Fig. S13) (Shu *et al.*, 2011 ▸; Gump *et al.*, 2007 ▸), showing a volume increase of ∼5% over this range. The expansion is anisotropic and occurs mainly along the *a* and *b* directions. High-pressure behaviour indicates a volume reduction of about 20% at 5 GPa, increasing to 24% at 9 GPa (Gump *et al.*, 2007 ▸; Deng *et al.*, 2025 ▸). Compression is anisotropic, with decreases of approximately 10% along the *a* and *b* axes, while the *c* parameter remains nearly unchanged.

To date, no systematic studies have investigated thermal expansion of TNP over a wide temperature range. However, crystallographic data are available at 100 K and 298 K, showing a modest increase in unit-cell volume of less than 3% (Nelyubina *et al.*, 2011 ▸). High-pressure behaviour has been studied up to 7.3 GPa (Fig. S14) (Atceken *et al.*, 2023 ▸). A volume decrease of about 15% is observed for polymorph I up to 4.4 GPa, followed by a transition to polymorph II. This transition is associated with significant changes in unit-cell parameters, including an increase in *a* and a decrease in *c*, while the overall volume remains continuous. At 7.3 GPa, polymorph II exhibits a total volume reduction of ∼22% compared to polymorph I at ambient pressure (Fig. S15) (Atceken *et al.*, 2023 ▸).

No detailed studies on thermal expansion of DAAF are available. However, crystallographic data from the CSD (KANQAR and KANQAR01) (Beal *et al.*, 2000 ▸) indicate a slight increase in unit-cell volume of ∼1% between 233 K and 295 K. High-pressure behaviour has been investigated up to 18 GPa (Fig. S16) and a volume reduction of approximately 27% is observed (Chellappa *et al.*, 2014 ▸). Compression is anisotropic, occurring preferentially along the *c* axis, followed by the *a* axis.

Thermal expansion of α-NTO has been determined by combining the low-temperature data of Bolotina & Pinkerton (2015 ▸) and the high-temperature data of Wang *et al.* (2025 ▸), covering the range 100–423 K (Fig. S17). An overall volume expansion of about 5% is observed, mainly along the *c* axis and then the *b* axis. For β-NTO, only low-temperature data are available (100–298 K) (Bolotina & Pinkerton, 2015 ▸). These results indicate anisotropic expansion, primarily along the *b* axis, with a volume increase of 2.6%. The high-pressure behaviour of NTO has been studied by Stavrou *et al.* (2016 ▸) using optical microscopy and interferometry methods up to 33 GPa. These authors report a volume reduction of approximately 43%. However, it should be noted that these measurements are not based on X-ray diffraction. As a result, only volumetric data are available and no information on unit-cell parameters or anisotropy can be obtained. Therefore, these results should be considered separately from diffraction-based studies.

Very limited data are available for DATB. The crystal structure has been reported at 223 K and 295 K (Kohno *et al.*, 2009 ▸; Holden, 1967 ▸), showing a small increase in unit-cell volume of less than 1%. No experimental high-pressure data are available. Molecular dynamics simulations by Kohno *et al.* (2009 ▸) suggest the evolution of unit-cell parameters up to 25 GPa, but these results remain unvalidated experimentally.

For TNB (form I), crystallographic data are available at 130 K, 183 K and 295 K (Yang *et al.*, 2020 ▸; Thallapally *et al.*, 2004 ▸; Choi & Prince, 1972 ▸). These data indicate a modest increase in unit-cell volume of ∼3.6% over this temperature range. No thermal expansion data are available for polymorphs II and III. In addition, no high-pressure crystallographic studies have been reported for this compound.

Thermal behaviour of BTF has been studied over the temperature range 150–450 K (Fig. S18), indicating anisotropic expansion, mainly along the *b* axis, with a total volume increase of ∼4.5% (Stankevich *et al.*, 2019 ▸). No high-pressure studies are available for this compound.

Thermal behaviour of DNAN has been investigated for polymorphs I and II. For I-DNAN, a volume expansion of ∼3.3% between 150 and 353 K, mainly along the *a* axis and then the *c* axis is reported (Stankevich *et al.*, 2024 ▸). For II-DNAN, Takahashi & Tamura (2015 ▸) and Stankevich *et al.* (2024 ▸) provide complementary datasets covering 100 K to the melting point. An overall volume increase of ∼6.5% is observed. Despite some discrepancies in absolute volume values, both studies agree on negative thermal expansion along the *b* axis and suggest a phase transition around 265 K, associated with a discontinuity in volume (Fig. S19). High-pressure behaviour of I-DNAN between 0 and 3 GPa shows an unusual expansion along the *b* axis, while the other parameters decrease, resulting in an overall volume reduction of 8.5% (Fig. S20) (Rajan *et al.*, 2022 ▸). At 3 GPa, a monoclinic-to-orthorhombic phase transition occurs, leading to a sharp decrease in volume. Above this pressure, the orthorhombic phase shows further compression of ∼6.25% up to 9 GPa, with a decrease along the *b* axis and a slight increase along the *a* axis.

Table 1 ▸ summarizes the temperature and pressure ranges over which the crystallographic parameters of each energetic material have been experimentally investigated. Where relevant, different polymorphs of a given compound are distinguished and linked to their corresponding literature references. CSD reference codes are also provided whenever available. This overview highlights the heterogeneity of available datasets and motivates the comparative analysis of thermoelastic properties presented in the following sections.

## 3. Thermal expansion of energetic molecular crystals

### 3.1. Data collection and determination of TECs

TECs of 12 energetic materials are summarized in Table 2 ▸. For consistency, TECs are reported in units of 10^−5^ K^−1^, allowing direct comparison between compounds. Both volumetric and, where available, linear expansion coefficients along the crystallographic axes are reported, together with the temperature ranges over which the measurements were performed.

TECs were preferentially taken from the original publications in which they were explicitly reported and derived using methodologies directly comparable to those employed in the present review. However, recalculation of the coefficients was performed in three situations: (i) when no TECs were reported by the original authors, (ii) when the reported coefficients were obtained using fitting procedures different from the linear regression approach adopted here and (iii) when complementary datasets from different studies covered distinct temperature ranges and could be combined to provide a more complete description of thermoelastic behaviour over an extended temperature interval. Indeed, most materials have been investigated over wide temperature ranges, extending from cryogenic conditions (*e.g.* 90 K for RDX, 10 K for PETN and 5 K for LLM-105) up to temperatures close to their melting points. However, complete datasets over such broad ranges are rarely obtained within a single study and it is often necessary to combine results from different authors (see Fig. 1 ▸). In Table 2 ▸, recalculated values are shown in italics, while literature values are not. For recalculated TECs, uncertainties correspond to the standard errors obtained from the linear regression procedure and therefore reflect the quality of the fit rather than the full experimental uncertainty associated with the original measurements.

### 3.2. Magnitude and anisotropy of thermal expansion

Data summarized in Table 2 ▸ show that despite differences in experimental conditions and data sources, volumetric TECs fall within a relatively narrow range, typically between 10 × 10^−5^ K^−1^ and 30 × 10^−5^ K^−1^. However, significant variability is observed between different studies for the same material. For instance, values reported for TATB and LLM-105 show noticeable discrepancies depending on the temperature range and experimental methodology, highlighting the sensitivity of these parameters to experimental conditions and data treatment.

While volumetric expansion remains relatively similar across materials, major differences arise in the anisotropy of thermal expansion. Materials composed of planar molecules, such as TATB, HNS, NTO or DNAN, tend to exhibit highly anisotropic thermal expansion, reflecting their layered crystal structures. In contrast, more compact, three-dimensionally packed molecules such as RDX, TNT, PETN and CL-20 generally show more isotropic expansion behaviour.

In some cases, negative linear thermal expansion is observed along specific crystallographic directions, as reported for γ-FOX-7 (Crawford *et al.*, 2007 ▸), γ-CL-20 (Bolotina & Pinkerton, 2015 ▸; Bolotina *et al.*, 2004 ▸; Pu *et al.*, 2016 ▸; Sun *et al.*, 2010 ▸) and II-DNAN (Takahashi & Tamura, 2015 ▸; Stankevich *et al.*, 2024 ▸), where one crystallographic axis contracts upon heating while the overall unit-cell volume continues to increase. Although relatively uncommon, this phenomenon is well documented in molecular crystals and generally reflects the presence of cooperative structural mechanisms operating within the crystal structure. Such behaviour is commonly attributed to hinge-like mechanisms, molecular tilting, layer shearing or rotations of relatively rigid molecular units, which may induce contraction along one crystallographic direction while expansion occurs along the other crystallographic directions (Bolotina & Pinkerton, 2015 ▸; Crawford *et al.*, 2007 ▸; Bolotina *et al.*, 2004 ▸; Takahashi & Tamura, 2015 ▸; Pu *et al.*, 2016 ▸; Stankevich *et al.*, 2024 ▸). The occurrence of such phenomena in γ-FOX-7 and γ-CL-20 is accompanied by a marked increase in thermoelastic anisotropy compared with the lower-temperature polymorphs, suggesting that polymorphic transformations can activate additional structural degrees of freedom responsible for these anomalous responses. Similar changes in thermoelastic anisotropy are also observed for DNAN, where polymorphic transitions significantly modify the expansion along specific crystallographic directions. In contrast, TNT represents a counter-example, since the transition between the orthorhombic and monoclinic forms induces only minor changes in the thermal expansion behaviour of the unit-cell parameters. These results demonstrate that thermal expansion in energetic molecular crystals is not only governed by overall intermolecular cohesion but also strongly influenced by crystal packing and polymorphism. In particular, highly anisotropic expansion appears to be associated with layered structures and may be linked to an increased propensity for structural transformations. Interestingly, no clear correlation is observed between volumetric thermal expansion and crystal density, in contrast to the behaviour observed for compressibility. Taken together, these observations suggest that negative linear thermal expansion in energetic molecular crystals is closely associated with anisotropic crystal packing and structural flexibility, particularly in layered systems undergoing polymorphic transformations.

It should be noted that the anisotropy discussed throughout this review is described with respect to the crystallographic axes. However, many energetic molecular crystals crystallize in low-symmetry systems, for which the principal directions of thermal expansion or compressibility do not necessarily coincide with the crystallographic axes. Determination of principal expansivities and compressibilities requires strain tensor analyses, such as those implemented in *PASCal*, which are not available for most of the datasets compiled here. Consequently, the present discussion is restricted to crystallographic directions. A systematic comparison of principal thermoelastic directions and their relationships with molecular packing motifs, hydrogen-bonding networks and layered arrangements would constitute a valuable direction for future investigations.

While thermal expansion provides insight into the temperature-dependent behaviour of energetic materials, their response to pressure is equally critical and is discussed in the following section in terms of compressibility and equation of state parameters.

## 4. Equation of state and compressibility of energetic molecular crystals

### 4.1. Data collection and equation of state parameters

The pressure dependence of the unit-cell volume is commonly described using analytical equations of state, among which the Birch–Murnaghan formalism is one of the most widely used in high-pressure crystallography (Birch, 1947 ▸). Table 3 ▸ summarizes the pressure ranges used to fit the Birch–Murnaghan equation of state. These ranges may be lower than the maximum pressures reached during the experiments, particularly when a phase transition is suspected. Where CIF files corresponding to the experiments are available in the Cambridge Structural Database (CSD), the corresponding reference codes are also indicated. For each material, the initial unit-cell volume *V*_0_, the bulk modulus *B*_0_ and its pressure derivative *B*′ are reported. These parameters, along with their corresponding uncertainties, are those directly obtained by the authors of the publication, except for two specific cases: PETN and one reference (Plisson *et al.*, 2017 ▸) on TATB where they were not mentioned. They have therefore been recalculated here using equation (2). The temperature at which these parameters were obtained is consistently specified in Table 3 ▸ when it differs from the ambient temperature. The compression ratios along each crystallographic direction and for the unit-cell volume are also systematically calculated. However, these ratios depend not only on the intrinsic properties of the material but also on the pressure range explored during the experiment. Moreover, the pressure derivative *B*′ shows significant variability and is often poorly constrained, particularly when limited pressure ranges are used. In some studies, it is fixed during the fitting procedure (such fixed values are indicated in the Table 3 ▸), which may introduce additional uncertainty in the derived bulk modulus values.

### 4.2. Magnitude and anisotropy of compression

**Figure 3:**
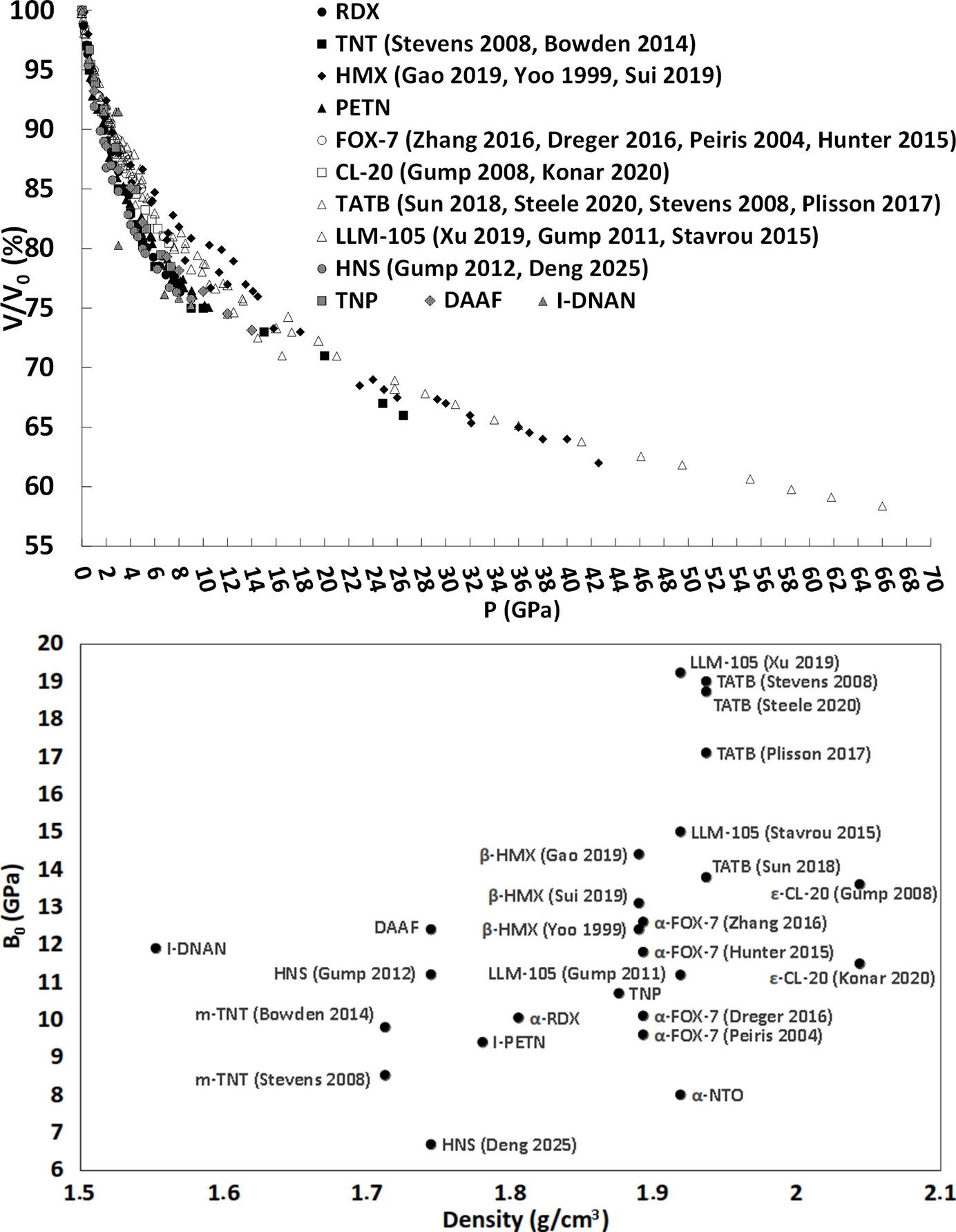
Pressure dependence of the normalized unit-cell volume *V*/*V*_0_ for 12 energetic molecular crystals (top). Bulk modulus *B*_0_ plotted as a function of the crystallographic density of the corresponding materials (bottom). Where several literature values are available for a given compound, the corresponding references are indicated in parentheses.

Most energetic materials exhibit bulk modulus values in the range 10–20 GPa, indicating a relatively similar overall compressibility. Fig. 3 ▸ (up) shows the evolution of normalized unit-cell volume as a function of pressure for several energetic materials with a volume reduction of approximately 15% at 5 GPa, 23% at 10 GPa, 28% at 20 GPa, 33% at 30 GPa and up to 38% at 50 GPa. However, noticeable discrepancies are observed between different studies for the same material, as illustrated for compounds such as HNS, LLM-105, TATB, FOX-7 and TNT. These differences likely arise from variations in experimental conditions, pressure calibration, data quality and refinement procedures. The effect of temperature on compressibility is illustrated for LLM-105 and CL-20, for which combined pressure–temperature studies show a decrease in *B*_0_ with increasing temperature. This behaviour is consistent with the expected softening of the crystal lattice at elevated temperature.

Anisotropic compression is observed for many energetic materials and is strongly influenced by molecular packing. Compounds composed of planar molecules, such as TATB, LLM-105, HNS, DAAF and DNAN, typically exhibit a preferred compression direction, reflecting their layered crystal structures. In contrast, more compact molecular systems such as HMX, PETN and CL-20 tend to display more isotropic compression behaviour.

In addition to the pronounced anisotropy of compression observed in several energetic molecular crystals, a few materials exhibit unusual axial responses under pressure. The most notable example is II-DNAN, for which a slight expansion of the *b* unit-cell parameter has been reported upon compression, corresponding to a negative linear compressibility over part of the investigated pressure range. Similarly, HNS displays an almost pressure-independent behaviour along the *c* direction, with only very limited contraction compared with the other crystallographic axes. Although rare, these anomalous responses illustrate that compressibility in energetic molecular crystals can involve cooperative structural mechanisms similar to those responsible for negative linear thermal expansion discussed in Section 3. As discussed previously for negative linear thermal expansion, these anomalous behaviours are generally associated with cooperative structural mechanisms such as layer shearing, hinge-like deformations, molecular tilting or rotations of relatively rigid molecular units. In layered molecular crystals, compression along one direction may induce transverse structural rearrangements that partially compensate, or even reverse, the expected contraction along another direction. These observations further emphasize that the compressibility of energetic molecular crystals is governed not only by intermolecular interaction strength but also by the geometry and flexibility of the crystal packing.

### 4.3. Relationship between density and bulk modulus

A more discriminating trend emerges when considering the relationship between bulk modulus and crystal density at ambient temperature for each polymorph, as shown in Fig. 3 ▸ (bottom). Overall, *B*_0_ tends to increase with density, indicating that denser materials are generally less compressible. This trend reflects the more efficient packing of molecules and stronger intermolecular interactions in high-density crystals. However, the relationship is characterized by a significant dispersion and cannot be adequately described by a simple linear model. This indicates that, although density constitutes an important parameter governing the mechanical response of energetic molecular crystals, it is not the sole controlling factor. Crystal packing arrangements, intermolecular interactions, hydrogen-bonding networks, structural anisotropy and polymorphism also contribute significantly to the observed variations in bulk modulus. Consequently, the relationship between density and compressibility should be regarded as a general qualitative trend rather than a predictive empirical law.

Two notable exceptions to this trend are the ɛ-CL-20 polymorph, which exhibits a very high density but a bulk modulus within the average range of values, and I-DNAN, which shows a relatively low density while having a bulk modulus comparable to those of denser energetic materials. However, for the latter compound, the monoclinic-to-ortho­rhombic phase transition occurring at around 3 GPa significantly limits the pressure range available for equation of state determination and the corresponding values should therefore be interpreted with caution.

Polymorphism also plays a key role in compressibility. High-pressure polymorphs generally exhibit higher densities, but their bulk moduli may either increase or decrease depending on the structural rearrangement, as observed for ɛ-FOX-7, II-DNAN and γ-RDX. This highlights the complex relationship between density, molecular packing and mechanical response.

Interestingly, while volumetric thermal expansion shows limited variation across materials, compressibility exhibits a clearer dependence on crystal density, highlighting the different physical mechanisms governing thermal and mechanical responses. Overall, these results demonstrate that while energetic molecular crystals share similar compressibility ranges, their detailed thermoelastic behaviour is strongly governed by crystal packing, density and polymorphism. The combination of these factors must therefore be considered to accurately describe their response under extreme conditions.

## 5. Conclusion

A literature review of the evolution of crystallographic parameters of 16 energetic molecular crystals under variable temperature and high-pressure conditions has been performed. The collected studies provide a consistent set of thermoelastic data, including TECs and equation of state parameters, which are essential for describing the behaviour of energetic materials under conditions relevant to storage and detonation.

Thermal expansion measurements show that, although volumetric expansion coefficients fall within a relatively narrow range (typically between 1.0 × 10^−4^ and 2.7 × 10^−4^ K^−1^) significant differences arise in the anisotropy of expansion. This anisotropy strongly depends on both the crystal structure and the polymorph. In particular, materials composed of planar molecules, such as TATB, LLM-105, HNS or FOX-7, tend to exhibit highly anisotropic thermal expansion, reflecting their layered crystal packing. In contrast, more compact molecular systems such as RDX, PETN or CL-20 generally display more isotropic behaviour.

High-pressure studies provide complementary information through the determination of equation of state parameters, most commonly described using the Birch–Murnaghan formalism. The bulk modulus *B*_0_ typically ranges between 10 and 20 GPa for most energetic molecular crystals, indicating a relatively similar overall compressibility. However, anisotropic compression is again observed for layered systems, whereas more isotropic behaviour is found for three-dimensionally packed structures. A qualitative tendency for denser materials to exhibit higher bulk moduli is observed, although significant deviations indicate that density alone is insufficient to predict compressibility.

Overall, this work provides a comparative framework for analysing the equation of state of energetic molecular crystals and emphasizes the importance of combining temperature- and pressure-dependent crystallographic data to achieve a comprehensive understanding of their physical behaviour. The compiled crystallographic data and derived parameters provide a basis for analysing the relationships between crystal structure, intermolecular interactions, anisotropy and the response of energetic molecular crystals to variations in temperature and pressure.

## Supplementary Material

Tables S1-S39, Figs. S1-S19. DOI: 10.1107/S2052520626008322/lo5133sup1.pdf

## Figures and Tables

**Table 1:** Temperature and pressure ranges over which the unit-cell parameters and unit-cell volumes of 16 energetic materials have been experimentally determined Corresponding Cambridge Structural Database (CSD) reference codes are provided where available.

Energetic materials	Temperature range (K)	Pressure range (GPa)	CSD reference code
RDX (C_3_H_6_N_6_O_6_)	α-RDX: 90–300 (Bolotina & Pinkerton, 2015 ▸)	α/γ-RDX: 0–8 (Oswald *et al.*, 2010 ▸)	
	α-RDX: 303–443 (Sun *et al.*, 2011 ▸)	ɛ-RDX: 0–5 (Millar, Oswald *et al.*, 2010 ▸)	
TNT (C_7_H_5_N_3_O_6_)	m-TNT: 123–300 (Vrcelj *et al.*, 2003 ▸)	m-TNT: 0–10 (Bowden *et al.*, 2014 ▸)	
	o-TNT: 123–300 (Vrcelj *et al.*, 2003 ▸)	m-TNT: 0–26.5 (Stevens *et al.*, 2008*a* ▸)	
HMX (C_4_H_8_N_8_O_8_)	β-HMX: 85–295 (Bolotina & Pinkerton, 2015 ▸)	β-HMX: 0–42.6 (Yoo & Cynn, 1999 ▸)	OCHTET14–OCHTET21 (Deschamps *et al.*, 2011 ▸)
	β-HMX: 123–295 (Deschamps *et al.*, 2011 ▸)	β-HMX: 0–40 (Sui *et al.*, 2019 ▸)	OCHTET44–OCHTET56 (Yan *et al.*, 2023 ▸)
	β-HMX: 173–433 (Yan *et al.*, 2023 ▸)	β-HMX: 0–14.5 (Gao *et al.*, 2019 ▸)	
PETN (C_5_H_8_N_4_O_12_)	I-PETN: 10–300 (Bolotina & Pinkerton, 2015 ▸)	I-PETN: 0–10.45 (Olinger *et al.*, 1975 ▸)	
	I-PETN: 150–370 (Stankevich *et al.*, 2019 ▸)		
FOX-7 (C_2_H_4_N_4_O_4_)	α/β-FOX-7: 200–393 (Evers *et al.*, 2006 ▸)	α-FOX-7: 0–4.14 (Hunter *et al.*, 2015 ▸)	SEDTUQ02–SEDTUQ08 (Evers *et al.*, 2006 ▸)
	α/β-FOX-7: 80–360 (Aree *et al.*, 2022 ▸)	α-FOX-7: 0–3.86 (Peiris *et al.*, 2004 ▸)	SEDTUQ15–SEDTUQ24 (Aree *et al.*, 2022 ▸)
	α/β/γ-FOX-7: 274–485 (McMonagle *et al.*, 2022 ▸)	α-FOX-7: 0–6.76 (Zhang *et al.*, 2016 ▸)	SEDTUQ33–SEDTUQ49 (Dreger *et al.*, 2016 ▸)
	α-FOX-7: 300–473 (Zhang *et al.*, 2016 ▸)	α-FOX-7: 0–12.8 (Dreger *et al.*, 2016 ▸)	
	γ-FOX-7: 200–493 (Crawford *et al.*, 2007 ▸)		
CL-20 (C_6_H_6_N_12_O_12_)	ɛ-CL-20: 25–115 (Liang *et al.*, 2022 ▸)	ɛ-CL-20: 0–12 (Konar *et al.*, 2020 ▸)	PUBMUU07–PUBMUU16 (Bolotina *et al.*, 2004 ▸)
	ɛ-CL-20: 298–388 (Bolotina & Pinkerton, 2015 ▸; Bolotina *et al.*, 2004 ▸)	ɛ-CL-20: 0–5.6 (Gump & Peiris, 2008 ▸; Gump *et al.*, 2007 ▸)	PUBMUU28–PUBMUU44 (Konar *et al.*, 2020 ▸)
	ɛ-CL-20: 100–298 (Pu *et al.*, 2016 ▸)		
	γ-CL-20: 303–403 (Bolotina & Pinkerton, 2015 ▸; Bolotina *et al.*, 2004 ▸)		
	γ-CL-20: 303–453 (Pu *et al.*, 2016 ▸)		
	α-CL-20: 303–393 (Pu *et al.*, 2016 ▸)		
	β-CL-20: 303–393 (Pu *et al.*, 2016 ▸)		
TATB (C_6_H_6_N_6_O_6_)	214–377 (Kolb & Rizzo, 1979 ▸)	0–40 (Steele *et al.*, 2020 ▸; Steele *et al.*, 2019 ▸)	
	303–513 (Sun *et al.*, 2010 ▸)	0–16 (Sun *et al.*, 2018 ▸)	
		0–66 (Plisson *et al.*, 2017 ▸)	
		0–13.2 (Stevens *et al.*, 2008*b* ▸)	
LLM-105 (C_4_H_4_N_6_O_5_)	5–360 (Xu *et al.*, 2019 ▸)	0–20 (Stavrou *et al.*, 2015 ▸)	
	298–513 (Gump *et al.*, 2011 ▸)	0–25 (Xu *et al.*, 2019 ▸)	
	303–473 (Li *et al.*, 2016 ▸)	0–5.4 (Gump *et al.*, 2011 ▸)	
*trans*-HNS (C_14_H_6_N_6_O_12_)	303–513 (Shu *et al.*, 2011 ▸)	0–9 (Deng *et al.*, 2025 ▸)	GIMBOT03–GIMBOT15 (Deng *et al.*, 2025 ▸)
	303–573 (Gump *et al.*, 2007 ▸)	0–5.4 (Gump *et al.*, 2007 ▸)	
I/II-TNP (C_3_HN_5_O_6_)	–	0–7.3 (Atceken *et al.*, 2023 ▸)	KAHHIM04–KAHHIM12 (Atceken *et al.*, 2023 ▸)
DAAF (C_4_H_4_N_8_O_3_)	–	0–16 (Chellappa *et al.*, 2014 ▸)	
NTO (C_2_H_2_N_4_O_3_)	α-NTO: 100–300 (Bolotina & Pinkerton, 2015 ▸)	α-NTO: 0–33.3 (Stavrou *et al.*, 2016 ▸)	QOYJOD01–QOYJOD05 (Bolotina *et al.*, 2003 ▸)
	α-NTO: 30–150 (Wang *et al.*, 2025 ▸)		
	β-NTO: 100–298 (Bolotina & Pinkerton, 2015 ▸; Bolotina *et al.*, 2003 ▸)		
DATB (C_6_H_5_N_5_O_6_)	–	–	
TNB (C_6_H_3_N_3_O_6_)	–	–	
o-BTF (C_6_N_6_O_6_)	150–450 (Stankevich *et al.*, 2019 ▸)	–	
DNAN C_7_H_6_N_2_O_5_	I-DNAN: 150–353 (Stankevich *et al.*, 2024 ▸)	I-DNAN: 0–3 (Rajan *et al.*, 2022 ▸)	FESNEW03–FESNEW21 (Takahashi & Tamura, 2015 ▸)
	II-DNAN: 150–353 (Stankevich *et al.*, 2024 ▸)	I-DNAN: 3–9 (Rajan *et al.*, 2022 ▸)	
	II-DNAN: 100–298 (Takahashi & Tamura, 2015 ▸)		

**Table 2:** Linear and volumetric thermal expansion coefficients (TECs) for 12 energetic molecular crystals reported in the literature Linear coefficients are given along the crystallographic axes *a*, *b* and *c*, and the corresponding volumetric coefficient α_*V*_ is also reported. The temperature range over which the coefficients were determined is indicated for each study. Italics are used to indicate recalculated values.

Materials	α_*a*_ (10^−5^ K^−1^)	α_*b*_ (10^−5^ K^−1^)	α_*c*_ (10^−5^ K^−1^)	α_*V*_ (10^−5^ K^−1^)	Temperature range (K), reference and CSD refcode where available
α-RDX	*2 (0.1)*	*7.74 (0.1)*	*5.98 (0.1)*	*15.94 (0.4)*	90–300 (Bolotina & Pinkerton, 2015 ▸)
	3.07 (n.r.)	8.28 (n.r.)	9.19 (n.r.)	20.66 (n.r.)	303–403 (Sun *et al.*, 2011 ▸)
	*2.42 (0.1)*	*7.95 (0.1)*	*7.13 (0.1)*	*17.92 (0.4)*	*Calculated over 90–433* (Bolotina & Pinkerton, 2015 ▸; Sun *et al.*, 2011 ▸)
m-TNT	4.6 (0.1)	5.2 (0.1)	9.5 (0.2)	20.6 (0.6)	123–300 (Vrcelj *et al.*, 2003 ▸)
o-TNT	3.9 (0.1)	4.9 (0.1)	10.7 (0.2)	19.8 (0.5)	123–300 (Vrcelj *et al.*, 2003 ▸)
β-HMX	*1.40 (0.1)*	*10.37 (0.3)*	*3.22 (0.1)*	*14.16 (0.5)*	85–295 (Bolotina & Pinkerton, 2015 ▸)
	0.043 (n.r.)	9.9 (n.r.)	3.3 (n.r.)	12.4 (n.r.)	123–303 (Deschamps *et al.*, 2011 ▸) (OCHTET14–OCHTET21)
	1.40 (0.01)	9 (0.84)	2.56 (0.12)	8.47 (0.14)	173–433 (Yan *et al.*, 2023 ▸) (OCHTET44–OCHTET56)
	*1.08 (0.15)*	*11.52 (0.3)*	*3.33 (0.1)*	*15.02 (0.4)*	*Calculated over 85–423* (Bolotina & Pinkerton, 2015 ▸; Deschamps *et al.*, 2011 ▸; Yan *et al.*, 2023 ▸)
I-PETN	*4.97 (0.2)*	*4.97 (0.2)*	*6.57 (0.2)*	*16.73 (0.7)*	10–300 (Bolotina & Pinkerton, 2015 ▸)
	7.62 (n.r.)	7.62 (n.r.)	9.30 (n.r.)	24.54 (n.r.)	150–370 (Stankevich *et al.*, 2019 ▸)
	*5.50 (0.2)*	*5.50 (0.2)*	*6.95 (0.2)*	*18.63 (0.7)*	*Calculated over 10–300* (Bolotina & Pinkerton, 2015 ▸; Stankevich *et al.*, 2019 ▸)
α-FOX-7	*2.74 (0.8)*	*13.05 (2)*	*4.32 (0.3)*	*18.33 (2.2)*	200–373 (Evers *et al.*, 2006 ▸) (SEDTUQ02–SEDTUQ08)
	*1.80 (0.1)*	*11.27 (0.4)*	*3.42 (0.1)*	*16.66 (0.6)*	80–360 (Aree *et al.*, 2022 ▸) (SEDTUQ15–SEDTUQ24)
	2.18 (0.07)	5.38 (0.03)	14.64 (0.08)	22.35 (0.13)	274–374 (McMonagle *et al.*, 2022 ▸)
	Not provided			14 (n.r.)	300–485 (Zhang *et al.*, 2016 ▸) (This work was not performed at atmospheric pressure)
β-FOX-7	1.12 (0.06)	5.78 (0.16)	18.19 (0.16)	25.19 (0.17)	380–460 (McMonagle *et al.*, 2022 ▸)
γ-FOX-7	*22.11 (0.5)*	*2.34 (0.4)*	*−0.36 (0.9)*	*24.60 (1.3)*	200–493 (Crawford *et al.*, 2007 ▸)
ɛ-CL-20	5.75 (n.r.)	6.43 (n.r.)	5.33 (n.r.)	16.67 (n.r.)	100–403 (Liang *et al.*, 2022 ▸)
	*4.24 (0.3)*	*4.63 (0.3)*	*4.23 (0.3)*	*12.15 (0.8)*	100–298 (Bolotina & Pinkerton, 2015 ▸; Bolotina *et al.*, 2004 ▸) (PUBMUU07–PUBMUU16)
	4.95 (n.r.)	4.91 (n.r.)	4.4 (n.r.)	13.5 (n.r.)	30–130 (Pu *et al.*, 2016 ▸)
	*4.46 (0.3)*	*4.25 (0.5)*	*4.51 (0.1)*	*12.49 (0.9)*	*Calculated over 100–403* (Bolotina & Pinkerton, 2015 ▸; Bolotina *et al.*, 2004 ▸; Liang *et al.*, 2022 ▸; Pu *et al.*, 2016 ▸)
γ-CL-20	*7.47 (0.4)*	*−0.55 (0.1)*	*4.95 (0.4)*	*10.1 (0.9)*	100–298 (Bolotina & Pinkerton, 2015 ▸; Bolotina *et al.*, 2004 ▸)
	8.72 (n.r.)	−0.75 (n.r.)	6.4 (n.r.)	11.9 (n.r.)	303–453 (Pu *et al.*, 2016 ▸)
	*8.28 (0.1)*	*−0.64 (0.1)*	*5.35 (0.1)*	*11.0 (0.2)*	*100–453* (Bolotina & Pinkerton, 2015 ▸; Bolotina *et al.*, 2004 ▸; Pu *et al.*, 2016 ▸)
α-CL-20	2.80 (n.r.)	5.26 (n.r.)	7.23 (n.r.)	15.39 (n.r.)	303–403 (Pu *et al.*, 2016 ▸)
β-CL-20	5.35 (n.r.)	1.48 (n.r.)	6.61 (n.r.)	13.52 (n.r.)	303–403 (Pu *et al.*, 2016 ▸)
TATB	0.83 (n.r.)	2.09 (n.r.)	24.8 (n.r.)	30.2 (n.r.)	214–377 (Kolb & Rizzo, 1979 ▸)
	1.04 (n.r.)	0.98 (n.r.)	16.9 (n.r.)	20.1 (n.r.)	303–513 (Sun *et al.*, 2010 ▸)
	*1.18 (0.2)*	*0.93 (0.2)*	*18.4 (1)*	*22.4 (1.4)*	*Calculated over 214–513* (Sun *et al.*, 2010 ▸; Kolb & Rizzo, 1979 ▸)
LLM-105	3.44 (n.r.)	8.47 (n.r.)	3.44 (n.r.)	15.8 (n.r.)	5–360 (Xu *et al.*, 2019 ▸) (The authors calculate the TECs from 160 to 360 K)
	*5.59 (0.4)*	*12.3 (0.5)*	*3.99 (0.9)*	*21.0 (0.9)*	298–513 (Gump *et al.*, 2011 ▸)
	3.31 (n.r.)	6.82 (n.r.)	3.06 (n.r.)	12.98 (n.r.)	303–473 (Li *et al.*, 2016 ▸)
	*3.26 (0.3)*	*8.75 (0.4)*	*2.95 (0.3)*	*14.72 (0.8)*	*Calculated over 160–513* (Xu *et al.*, 2019 ▸; Li *et al.*, 2016 ▸; Gump *et al.*, 2011 ▸)
*trans*-HNS	7.67	6.80	1.12	16.72	303–515 (Shu *et al.*, 2011 ▸)
	*7.91 (0.4)*	*10.04 (0.5)*	*2.84 (0.4)*	21 (0.56)	303–573 (Gump *et al.*, 2012 ▸)
α-NTO	2.3 (n.r.)	6.2 (n.r.)	9.1 (n.r.)	17.7 (n.r.)	100–300 (Bolotina & Pinkerton, 2015 ▸)
	1.4 (n.r.)	5.3 (n.r.)	8.2 (n.r.)	15.3 (n.r.)	303–423 (Wang *et al.*, 2025 ▸)
	*1.93 (0.1)*	*6.12 (0.2)*	*7.94 (0.1)*	*16.86 (0.4)*	*Calculated over 100–423* from Bolotina & Pinkerton (2015 ▸) and Wang *et al.* (2025 ▸)
β-NTO	*0.44 (0.2)*	*6.95 (0.3)*	*4.67 (0.1)*	*13.20 (0.2)*	100–298 (Bolotina & Pinkerton, 2015 ▸; Bolotina *et al.*, 2003 ▸) (QOYJOD01–QOYJOD05)
o-BTF	*4.18 (0.1)*	*8.48 (0.3)*	*1.31 (0.1)*	*14.12 (0.4)*	150–450 (Stankevich *et al.*, 2019 ▸)
I-DNAN	*12.15 (0.3)*	*0.11 (0.1)*	*4.67 (0.2)*	*15.70 (0.3)*	150–353 (Stankevich *et al.*, 2024 ▸)
II-DNAN	*20.32 (0.5)*	*−2.67 (0.6)*	*2.02 (0.4)*	*22.69 (0.9)*	150–353 (Stankevich *et al.*, 2024 ▸)
II-DNAN	*22.27 (1.4)*	*−3.15 (0.4)*	*4.07 (0.3)*	*27.07 (2)*	100–298 (Takahashi & Tamura, 2015 ▸) (FESNEW03–FESNEW21)

**Table 3:** Summary of equation of state parameters for 12 energetic materials and corresponding literature references Temperature is indicated when it differs from ambient temperature.

Energetic materials	Birch–Murnaghan coefficients			Compression ratio at the end of the experiments				Pressure range (GPa), reference and CSD reference
	*V*_0_ (Å^3^)	*B*_0_ (GPa)	*B*′	Δ*a*/*a*_0_ (%)	Δ*b*/*b*_0_ (%)	Δ*c*/*c*_0_ (%)	Δ*V*/*V*_0_ (%)	
α-RDX	1640 (2)	10.05 (5)	11.30 (7)	–	–	–	84.5	0–3.9 (Oswald *et al.*, 2010 ▸)
γ-RDX	1632 (4)	8.73 (16)	11.26 (fixed)	–	–	–	76.5	4–8.1 (Oswald *et al.*, 2010 ▸)
ɛ-RDX	808.30 (3.7)	10.34 (0.84)	7.78 (0.31)	–	–	–	80.5	1–5 (Millar, Oswald *et al.*, 2010 ▸)
m-TNT	1828.8 (n.r.)	9.80 (0.4)†	8.20 (0.3)†	93.3	93.8	88.5	75	0–10 (Bowden *et al.*, 2014 ▸)
	1828.8 (n.r.)	8.52 (n.r.)	8 (n.r.)	–	–	–	71	0–20 (Stevens *et al.*, 2008*a* ▸)
β-HMX	519.39 (n.r.)	12.4 (n.r.)	10.4 (n.r.)	86.8	83.1	93.7	67.5	0–27 (Yoo & Cynn, 1999 ▸)
	519.24 (n.r.)	13.1 (n.r.)	11.1 (n.r.)	–	–	–	76	0– 15 (Sui *et al.*, 2019 ▸)
	521.6 (3.5)	14.4 (4.2)	12.8 (5.5)	95.6	95.9	96.2	87.6	0–4.2 (Gao *et al.*, 2019 ▸)
	482.7 (1.4)	65.1 (2.9)	0.7 (0.3)	93.6	91.9	95	80.9	6.2–9.1 (Gao *et al.*, 2019 ▸)
I-PETN	*590.84* (n.r.)	*9.4*‡ (n.r.)	*11.3*‡ (n.r.)	*91.2*	*–*	*90.2*	*75*	0–10.45 (Olinger *et al.*, 1975 ▸)
α-FOX-7	521.07 (0.04)	11.81 (0.47)	11.41 (0.95)	97	91.5	96.7	85.8	0–4.1 (Hunter *et al.*, 2015 ▸)
	514.60 (3.3)	9.6 (1.6)	20.8 (4.3)	97.7	92	97.2	87.5	0–3.86 (Peiris *et al.*, 2004 ▸)
	516.76 (n.r.)	12.6 (1.4)	11.3 (2.1)	–	–	–	81	0–6.76 (Zhang *et al.*, 2016 ▸)
	522.92 (0.06)	10.1 (0.7)	14.3 (1.7)	96.6	90.9	96.7	77.5	0–4.5 (Dreger *et al.*, 2016 ▸) (SEDTUQ34–SEDTUQ44)
ɛ-FOX-7	261.46 (0.03)	15.5 (1.6)	6.3 (0.9)	93.7	98.3	98.5	89.8	4.5–12.8 (Dreger *et al.*, 2016 ▸) (SEDTUQ33; SEDTUQ45–SEDTUQ49)
ɛ-CL-20	1430.04 (83)	11.49 (20)	11.16 (23)	94.1	91	92.6	80.4	0–7.2 (Konar *et al.*, 2020 ▸) (PUBMUU28–PUBMUU44)
	1422.34 (5.14)	13.6 (2)	11.7 (3.2)	95	93.1	94.5	84.7	0–5.6 (Gump & Peiris, 2008 ▸; Bolotina *et al.*, 2003 ▸)
	1427.02 (3.59)	11 (1.3)	14 (2.7)	95.2	92	94.2	83.7	0–5.6 (Gump & Peiris, 2008 ▸; Gump *et al.*, 2007 ▸) (*T* = 348 K)
TATB	442.5 (n.r.)	13.6 (0.6)	12.4 (1.1)	–	–	–	81.3	0–8 (Stevens *et al.*, 2008*b* ▸)
	442.5 (n.r.)	17.1 (0.3)	8.1 (1.4)	–	–	–	75.8	0–13.2 (Stevens *et al.*, 2008*b* ▸)
	450 (n.r.)	18.73 (n.r.)	5.19 (n.r.)	95	94.6	81.8	71	0–16 (Sun *et al.*, 2018 ▸)
	*443.80* (n.r.)	*13.79*‡ (n.r.)	*10.35*‡ (n.r.)	91	89.9	74.4	58.3	0–66 (Plisson *et al.*, 2017 ▸)
	442.64 (n.r.)	19 (3)	4 (fixed)§	–	–	–	87.3	0–3.5 (Steele *et al.*, 2019 ▸)
	424.8 (n.r.)	45 (5)	4 (fixed)§	–	–	–	80.7	4–16.2 (Steele *et al.*, 2019 ▸)
	443.9 (n.r.)	18.5 (5)	4 (fixed)§	97.1	97.2	92.1	85.7	0–3.5 (Steele *et al.*, 2020 ▸)
	421.7 (n.r.)	27.9 (8)	6.6 (4)	92.3	92.6	80	65.6	4–41 (Steele *et al.*, 2020 ▸)
LLM-105	747.3 (1.2)	11.19 (0.02)	18.54 (0.04)	96.2	91.5	95.5	84.2	0–5.4 (Gump *et al.*, 2011 ▸)
	754.1 (1.5)	4.61 (0.02)	29.8 (0.2)	96.3	90.5	96	84.5	0–5.5 (Gump *et al.*, 2011 ▸) (*T* = 373 K)
	768.4 (0.9)	0.74 (0.01)	233 (29)	96.3	90.6	95.6	85.9	0–5 (Gump *et al.*, 2011 ▸) (*T* = 453 K)
	750.1 (n.r.)	15.32 (n.r.)	8.96 (n.r.)	90	85.8	91.9	72.3	0–20 (Stavrou *et al.*, 2015 ▸)
	747.38 (n.r.)	19.23 (n.r.)	6.7 (n.r.)	93.9	83.4	91	68.2	0–25 (Xu *et al.*, 2019 ▸)
*trans*-HNS	1702.59 (n.r.)	11.2 (0.66)	6.2 (0.69)	89.8	89.5	100.1	80	0–5.4 (Gump *et al.*, 2007 ▸)
	1715.47 (n.r.)	6.68 (8)	14.20 (6)	92.4	82.5	98.9	76	0–9 (Deng *et al.*, 2025 ▸) (GIMBOT03–GIMBOT15)
I-TNP	2178 (5)	10.7 (7)	8.9 (8)	94.4	92.9	96.9	85	0–4.4 (Atceken *et al.*, 2023 ▸) (KAHHIM04–KAHHIM12)
DAAF	798.7 (0.05)	9.14 (0.49)	8.26 (0.37)	89.2	94.4	86.7	73	0–16 (Chellappa *et al.*, 2014 ▸)
α-NTO	902.06 (fixed)	8 (1.6)	10.6 (2.3)	–	–	–	56.7	0–33.3 (Stavrou *et al.*, 2016 ▸)
I-DNAN	1693.4 (n.r.)	11.9 (n.r.)	10 (n.r.)	92.3	100	96.6	91.5	0–3 (Rajan *et al.*, 2022 ▸)
o-DNAN	1360 (n.r.)	26.9 (n.r.)	10 (n.r.)	1.03	93.9	97.6	93.8	3–9 (Rajan *et al.*, 2022 ▸)

† Three experiments were performed; the intermediate values are reported here.

‡ Values recalculated in the present work from literature data using the third-order Birch–Murnaghan equation of state.

§ Values fixed by the authors and not refined during the fitting procedure.
