# Supplementary material for: Crystallography of organic energetic materials: thermoelastic properties and equation of state
Source: Acta Crystallogr B Struct Sci Cryst Eng Mater. 2026 Sep 15;82(Pt 5):449–61. doi: 10.1107/S2052520626008322 (PMC13729041; doi:10.1107/S2052520626008322)
Supplement: Supplementary file 1 [file b-82-00449-sup1.pdf]

STRUCTURAL SCIENCE
CRYSTAL ENGINEERING
MATERIALS

Volume 82 (2026)

Supporting information for article:

Crystallography of organic energetic materials: thermoelastic properties and equation of state

Mathieu Guerain

Supplementary Information

1) Data processing and fitting method

Table S1 : Chemical formula, name, abbreviations and molecular structure of the 16 energetic materials discussed in this work.

Energetic compounds	Abbreviations	Molecular structure
Cyclotrimethylene-trinitramine $C_3H_6N_6O_6$	RDX	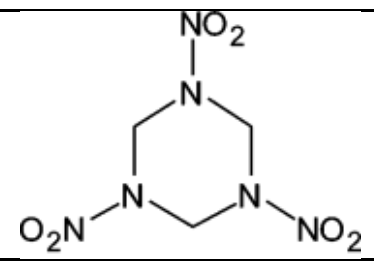
2,4,6-Trinitrotoluene $C_7H_5N_3O_6$	TNT	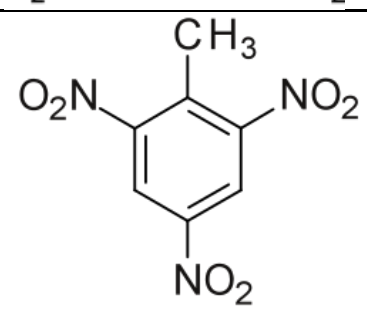
1,3,5,7-Tetranitro-1,3,5,7-tetra-azacyclo-octane $C_4H_8N_8O_8$	HMX	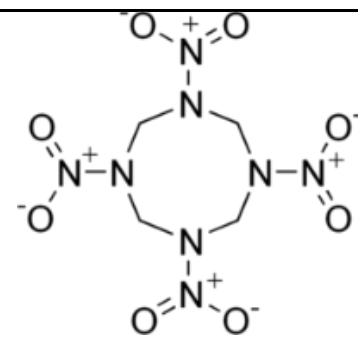
Pentaerythritol tetranitrate $C_5H_8N_4O_{12}$	PETN	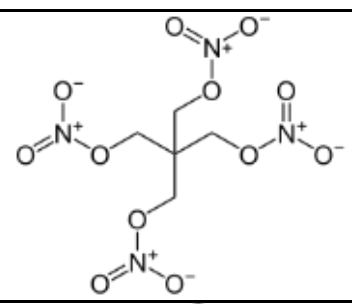
1,1-Diamino-2,2-dinitroeth-ylene $C_2H_4N_4O_4$	FOX-7	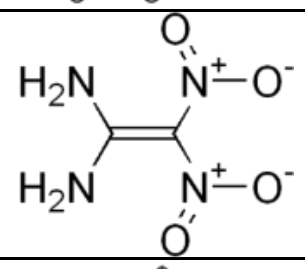
hexanitrohexa-azaisowurtzitane $C_6H_6N_{12}O_{12}$	CL-20	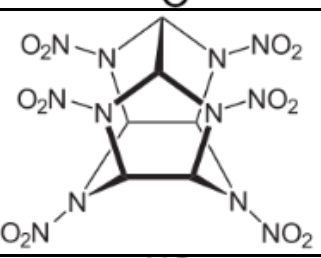
1,3,5-Triamino-2,4,6-trinitro-benzene $C_6H_6N_6O_6$	TATB	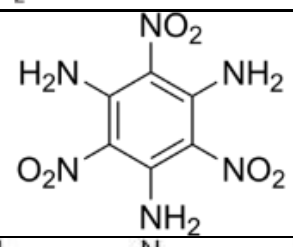
2,6-Diamino-3,5-dinitro-1,4-pyrazine 1-oxide $C_4H_4N_6O_5$	LLM-105	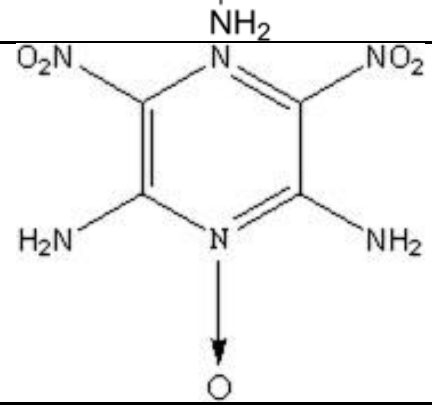

2,2',4,4',6,6'-Hexanitrostilbene $C_{14}H_6N_6O_{12}$	HNS	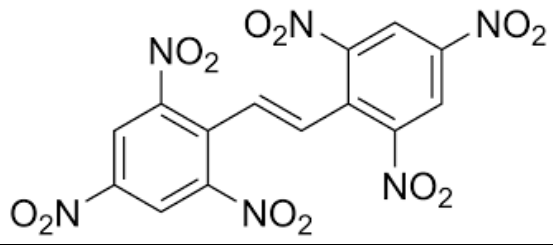
3,4,5-Trinitro-1H-pyrazole $C_3HN_5O_6$	TNP	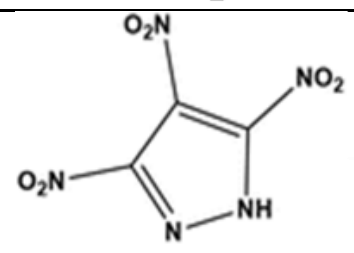
4,4'-Azoxybis-1,2,5-oxadiazol-3-amine $C_4H_4N_8O_3$	DAAF	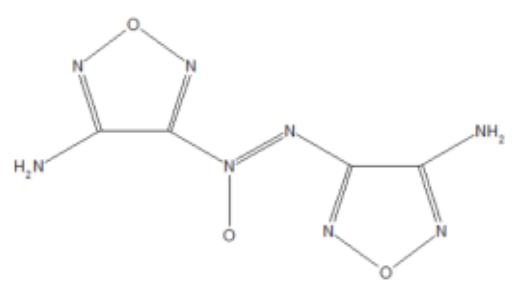
5-Nitro-2,4-dihydro-3H-1,2,4-triazol-3-one $C_2H_2N_4O_3$	NTO	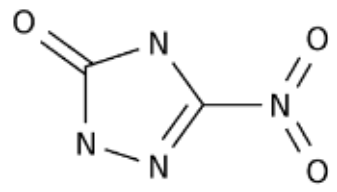
1,3-Diamino-2,4,6-trinitrobenzene $C_6H_5N_5O_6$	DATB	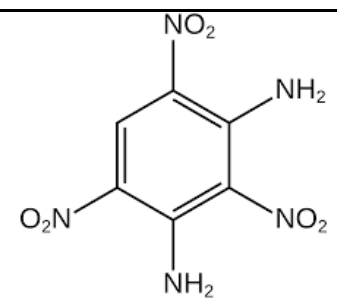
1,3,5-Trinitrobenzene $C_6H_3N_3O_6$	TNB	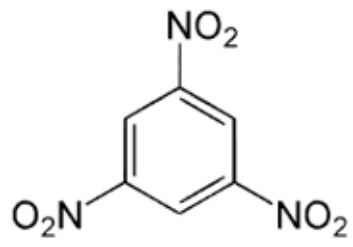
Benzotrifuroxan $C_6N_6O_6$	BTF	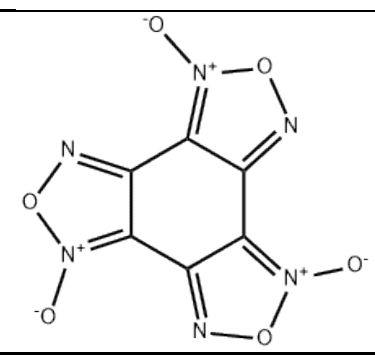
2,4-Dinitroanisole $C_7H_6N_2O_5$	DNAN	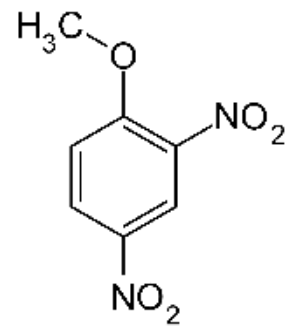

Table S2 : Methodology used for collection of lattice parameters, cell volume, and thermal expansion coefficients (TECs) calculation.

Energetic materials	Reference	Lattice parameters and cell volume collection	TEC calculation
α-RDX	[1]	Numerical Table	The values were recalculated in this work
	[2]	Numerical Table	Values calculated by the original authors. No uncertainties are given.
m-TNT	[3]	Graphical extraction	Values and uncertainties calculated by the original authors.
o-TNT	[3]	Graphical extraction	Values and uncertainties calculated by the original authors.
β-HMX	[1]	Numerical Table	The values were recalculated in this work
	[4]	Numerical Table	Values calculated by the original authors. No uncertainties are given.

	5	Numerical table. The original authors performed experiments on three samples and report three values. An average value was systematically calculated.	Values and uncertainties calculated by the original authors.
I-PETN	1	Numerical Table	Values and uncertainties calculated by the original authors.
	6	Graphical extraction	The values were recalculated in this work
α-FOX-7	7	Graphical extraction	The values were recalculated in this work
	8	Numerical Table	The values were recalculated in this work
	9	Graphical extraction	Values and uncertainties calculated by the original authors.
β-FOX-7	9	Graphical extraction	Values and uncertainties calculated by the original authors.
γ-FOX-7	10	Numerical Table	The values were recalculated in this work
ε-CL-20	1.12	Numerical Table	The values were recalculated in this work
	12	Numerical Table	Values calculated by the original authors. No uncertainties are given.
	13	Graphical extraction	Values calculated by the original authors. No uncertainties are given.
γ-CL-20	1.12	Numerical Table	The values were recalculated in this work
	13	Graphical extraction	Values calculated by the original authors. No uncertainties are given.
α-CL-20	13	Graphical extraction	Values calculated by the original authors. No uncertainties are given.
β-CL-20	13	Graphical extraction	Values calculated by the original authors. No uncertainties are given.
TATB	14	Numerical Table	Values calculated by the original authors. No uncertainties are given.
	15	Numerical Table	Values calculated by the original authors. No uncertainties are given.
LLM-105	16	Graphical extraction	Values calculated by the original authors. No uncertainties are given.
	17	Numerical Table	The values were recalculated in this work
	18	Numerical Table	Values calculated by the original authors. No uncertainties are given.
Trans-HNS	19	Numerical Table	Values calculated by the original authors. No uncertainties are given.
	20	Graphical extraction	The values for a, b and c were recalculated in this work. The value and uncertainties for V were calculated by the original authors.
α-NTO	1	Numerical Table	Values calculated by the original authors. No uncertainties are given.
	21	Graphical extraction	Values calculated by the original authors. No uncertainties are given.

β-NTO	[1]	Numerical Table	The values were recalculated in this work
Ortho-BTF	[6]	Graphical extraction	The values were recalculated in this work
I-DNAN	[22]	Graphical extraction	The values were recalculated in this work
II-DNAN	[22]	Graphical extraction	The values were recalculated in this work
	[23]	Numerical Table	The values were recalculated in this work

Table S3 : Methodology used for collection of lattice parameters, cell volume, and Birch-Murnaghan parameters: V_0 , Bulk modulus B and B' the derivative of Bulk Modulus. The densities of the different polymorph at ambient temperature are provided. Additional information's on the experimental setup are also described: Use of powder or single crystal as a sample, X-Ray or neutron diffraction, and Pressure transmitting medium.

Energetic materials and [density at ambient temperature in g/cm^3]	Reference	Lattice parameters and cell volume collection	V_0, B and B' collection	Experimental setup: -powder or single crystal -X-Ray or neutron diffraction -Pressure transmitting medium (PTM)
α-RDX [1.806]	[24,25]	Numerical Table	Values and uncertainties calculated by the original authors.	Single crystal Neutron 4:1 perdeuterated methanol-ethanol
γ-RDX [2.267]	[25]	Numerical Table	Values and uncertainties calculated by the original authors.	Single crystal Neutron 4:1 perdeuterated methanol-ethanol
ε-RDX [2.266]	[24,26]	Numerical Table	Values and uncertainties calculated by the original authors.	Single crystal Neutron 4:1 perdeuterated methanol-ethanol
m-TNT [1.713]	[27]	Graphical extraction	Values calculated by the original authors. No uncertainties are given for V_0	Powder X-Ray No PTM
	[28]	Numerical Table	Values calculated by the original authors. No uncertainties are given.	Powder X-Ray Liquid Nitrogen or No PTM
β-HMX [1.89]	[29]	Numerical Table	Values calculated by the original authors. No uncertainties are given.	Powder X-Ray Argon or No PTM
	[30]	Numerical Table	Values calculated by the original authors. No uncertainties are given.	Single crystal X-Ray Neon
	[31]	Numerical Table	Values and uncertainties calculated by the original authors.	Powder X-Ray No PTM

I-PETN [1.781]	32	Numerical Table	The values were re-calculated in this work	Powder X-Ray 4:1 methanol-ethanol
α-FOX-7 [1.893]	33	Graphical extraction	Values calculated by the original authors. No uncertainties are given for V_0	Powder X-Ray Amorphous boron-epoxy resin
	34	Numerical Table	Values and uncertainties calculated by the original authors.	Powder Neutron 4:1 perdeuterated methanol-ethanol
	35	Numerical Table	Values and uncertainties calculated by the original authors.	Powder X-Ray No PTM
	36	Numerical Table	Values and uncertainties calculated by the original authors.	Single crystal X-Ray 4:1 Methanol-ethanol
ε-FOX-7 [2.308]	36	Numerical Table	Values and uncertainties calculated by the original authors.	Single crystal X-Ray 4:1 Methanol-ethanol
ε-CL-20 [2.044]	37	From CIF	Values and uncertainties calculated by the original authors.	Powder X-Ray 4:1 Methanol-ethanol Powder Neutron 4:1 perdeuterated Methanol-ethanol
	38,39	Numerical Table	Values and uncertainties calculated by the original authors.	Powder X-Ray Dow Corning 200 Fluid
TATB [1.937]	40	Numerical Table	Values calculated by the original authors. No uncertainties are given for V_0	Powder X-Ray 4:1 Methanol-ethanol
	41	Graphical extraction	Values calculated by the original authors. No uncertainties are given.	Powder X-Ray Helium
	42	Numerical Table	The values were re-calculated in this work	Powder X-Ray No PTM<br><br>Single crystal X-Ray Neon
	43,44	Numerical table between 0 and 3.5 GPa, graphical extraction above 3.5 GPa	Values calculated by the original authors. No uncertainties are given for V_0	Powder X-Ray Neon

LLM-105 [1.919]	[16]	Graphical extraction	Values calculated by the original authors. No uncertainties are given.	Powder<br>X-Ray<br>4:1 methanol-ethanol
	[45]	Graphical extraction	Values calculated by the original authors. No uncertainties are given.	Single crystal<br>X-Ray<br>Silicone Oil
	[17]	Numerical Table	Values and uncertainties calculated by the original authors.	Powder<br>X-Ray<br>Dow Corning 200 Fluid
Trans-HNS [1.745]	[17,20]	Graphical extraction	Values calculated by the original authors. No uncertainties are given for V_0	Powder<br>X-Ray<br>Dow Corning 200 Fluid
	[46]	From CIF	Values calculated by the original authors. No uncertainties are given for V_0	Single crystal<br>X-Ray<br>4:1 Methanol/Ethanol
I-TNP [1.876]	[47]	Numerical Table	Values and uncertainties calculated by the original authors.	Powder<br>Neutron<br>1:1 perdeuterated *n*-pentane/iso-pentane<br><br>Single crystal<br>X-Ray<br>1:1 perdeuterated *n*-pentane/iso-pentane
DAAF [1.745]	[48]	Graphical extraction	Values and uncertainties calculated by the original authors.	Powder<br>X-Ray<br>Nitrogen/neon/no PTM
α-NTO [1.919]	[49]	Graphical extraction	Values and uncertainties calculated by the original authors.	Interferometry measurements
I-DNAN [1.553]	[50]	Graphical extraction	Values calculated by the original authors. No uncertainties are given.	Powder<br>X-Ray<br>No PTM
II-DNAN [1.556]	[50]	Graphical extraction	Values calculated by the original authors. No uncertainties are given.	Powder<br>X-Ray<br>No PTM

2) Lattice parameters and cell volume in function of temperature and pressure available in literature

Tables S4–S39 compile the complete lattice-parameter and unit-cell-volume datasets used for the determination of thermal expansion coefficients and Birch–Murnaghan equation-of-state parameters discussed in the main text. Data are reproduced from the original publications, supplementary materials, doctoral theses, or digitized from published figures when numerical values were otherwise unavailable.

1) RDX

Table S4 : Lattice parameters and cell volume of α-RDX in function of temperature

Energetic materials	T (°C)	a (Å)	b (Å)	c (Å)	Volume (Å^3)	V/Z (Å^3)
α-RDX Bolotina et al.[1]	-183	13.145	11.425	10.593	1590	198.75
	-168	13.147	11.437	10.599	1593	199.125
	-153	13.149	11.448	10.605	1596	199.5
	-133	13.153	11.464	10.615	1600	200
	-113	13.156	11.481	10.626	1605	200.625
	-93	13.163	11.499	10.639	1610	201.25
	-73	13.172	11.518	10.652	1616	202
	-53	13.176	11.537	10.665	1621	202.625
	-33	13.181	11.554	10.678	1626	203.25
	-13	13.185	11.571	10.693	1631	203.875
	7	13.192	11.591	10.707	1637	204.625
	27	13.199	11.61	10.728	1643	205.375
α-RDX Sun et al.[2]	30	13.1997	11.6089	10.7244	1643.35	205.418
	50	13.2088	11.6292	10.7426	1650.14	206.2675
	70	13.2157	11.6484	10.7599	1656.40	207.05
	90	13.2230	11.6670	10.7779	1662.74	207.842
	110	13.2314	11.6868	10.7978	1669.7	208.712
	130	13.2380	11.7045	10.8166	1675.98	209.497
	150	13.2499	11.7263	10.8414	1684.45	210.556
	170	13.2567	11.7428	10.8624	1690.96	211.37

Table S5 : Lattice parameters and cell volume of α-RDX in function of Pressure

Energetic materials	P (GPa)	a (Å)	b (Å)	c (Å)	Volume (Å^3)	V/Z (Å^3)
α-RDX Millar et al.[24] Oswald et al.[25]	0	13.192	11.602	10.723	1641.1	205.13
	0.12	13.153	11.542	10.663	1618.9	202.36
	0.46	13.075	11.452	10.553	1580	197.5
	0.82	13.008	11.351	10.454	1543.6	192.95
	1.35	12.93	11.248	10.346	1504.8	188.1
	1.82	12.868	11.166	10.264	1474.8	184.35
	2.43	12.809	11.08	10.181	1445	180.62
	3.01	12.754	11.005	10.104	1418.2	177.27
	3.62	12.703	10.938	10.032	1394	174.25
	3.85	12.686	10.912	10.013	1386.2	173.27

Table S6 : Lattice parameters and cell volume of γ-RDX in function of Pressure

Energetic materials	P (GPa)	a (Å)	b (Å)	c (Å)	Volume (Å^3)	V/Z (Å^3)
γ-RDX Oswald et al.[25]	3.98	12.698	9.651	11.085	1358.3	169.78
	4.33	12.6691	9.6096	11.048	1345.1	168.13
	4.87	12.6321	9.5554	11.003	1328.2	166.025
	5.37	12.5975	9.5096	10.963	1313.4	164.17

	5.88	12.5625	9.4725	10.925	1300.1	162.512
	6.39	12.533	9.4344	10.894	1288.1	161.012
	6.91	12.5025	9.4007	10.8555	1275.86	159.482
	7.45	12.4718	9.3659	10.825	1264.5	158.062
	8.07	12.4366	9.3321	10.788	1252	156.5

Table S7 : Lattice parameters and cell volume of ε-RDX in function of Pressure

Energetic materials	P (GPa)	a (Å)	b (Å)	c (Å)	Volume (Å ^{3})	V/Z (Å ^{3})
ε-RDX Millar et al.[24]	0.99	7.3589	11.2064	10.719	751.33	187.83
	1.23	7.3404	11.1501	10.465	743.91	185.97
	1.32	7.3213	11.1235	9.1107	739.35	184.83
	1.59	7.2975	11.0659	9.0891	731.24	182.81
	1.8	7.2759	11.0159	9.0786	723.71	180.92
	2.1	7.2458	10.9616	9.0552	715.27	178.81
	2.51	7.2164	10.8977	9.0294	705.72	176.43
	2.89	7.1875	10.8383	9.0056	696.99	174.24
	3.16	7.1702	10.8038	8.9737	691.53	172.88
	3.65	7.1355	10.7355	8.9471	681.37	170.34
	4.33	7.1062	10.6810	8.9269	672.55	168.13
	4.64	7.0849	10.6373	8.8947	666.64	166.66
	5.04	7.0655	10.6043	8.8608	661.00	165.25

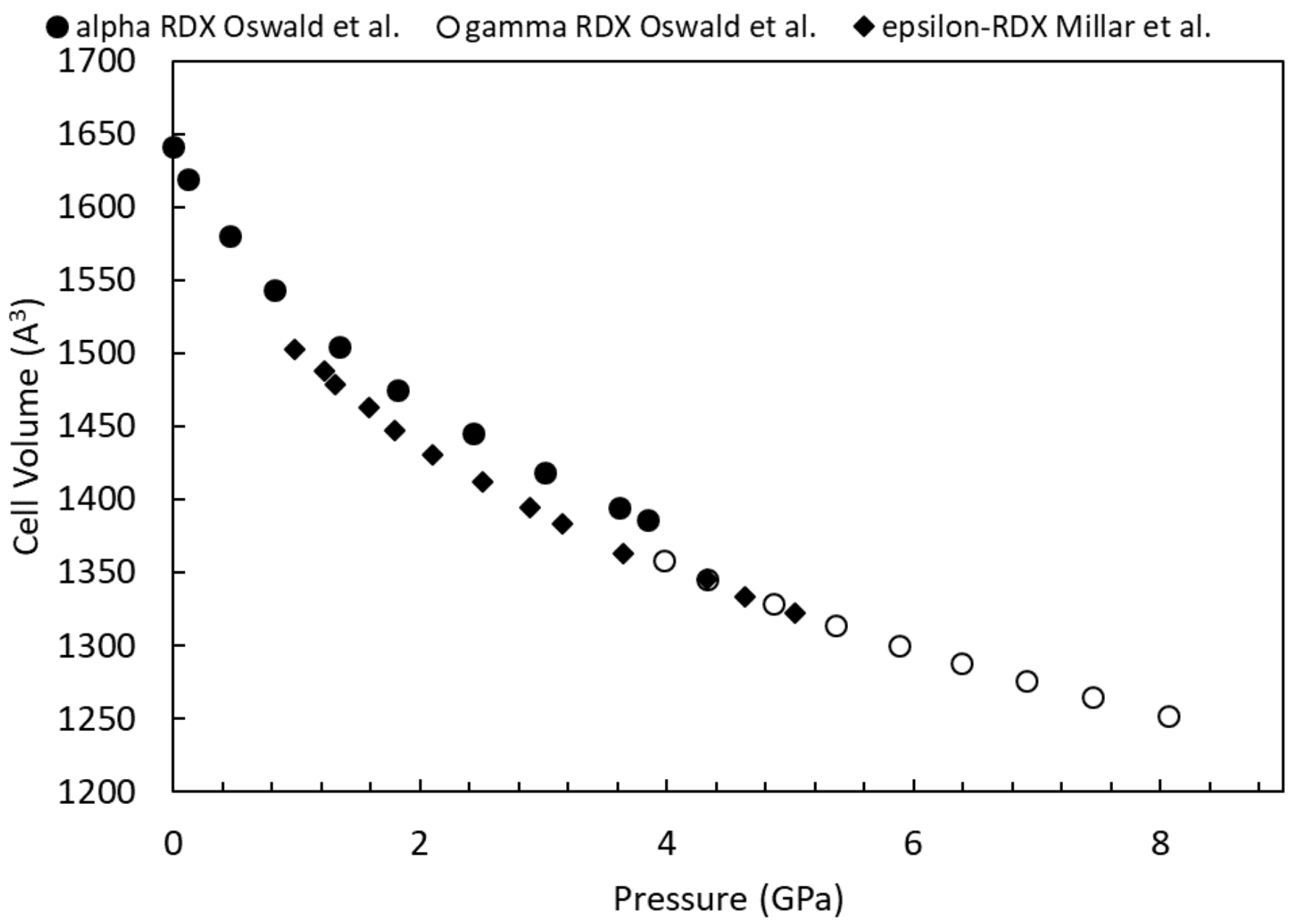

Figure S1 : Variation of cell volume of α, γ and ε-RDX with pressure. For graphical comparison with α-RDX and γ-RDX, the unit-cell volumes of ε-RDX have been multiplied by a factor of two. The original crystallographic volumes are reported in Table S7.

2) TNT

Table S8 : Lattice parameters and cell volume of m-TNT in function of temperature

Energetic materials	T (°C)	a (Å)	b (Å)	c (Å)	β (°)	Volume (Å ^{3})

m-TNT Vrcelj et al.[3]	-150	14.911	6.034	20.881	110.365	1761.37
	-120	14.941	6.046	20.923	110.309	1772.59
	-100	14.956	6.052	20.985	110.254	1782.08
	-70	14.9634	6.055	21.006	110.210	1786.14
	-50	14.970	6.064	21.048	110.177	1793.64
	-25	14.985	6.070	21.132	110.144	1804.73
	0	15.015	6.082	21.194	110.033	1818.57
	27	15.060	6.100	21.320	109.923	1841.52

Table S9 : Lattice parameters and cell volume of o-TNT in function of temperature

Energetic materials	T (°C)	a (Å)	b (Å)	c (Å)	Volume (Å^3)
o-TNT Vrcelj et al.[3]	-150	14.91	6.034	19.68	1770.54938
	-120	14.92491	6.043051	19.71936	1778.52837
	-100	14.93982	6.049085	19.7784	1787.41833
	-70	14.95473	6.055119	19.83744	1796.33315
	-50	14.96964	6.06417	19.89648	1806.17145
	-25	14.98455	6.070204	19.926	1812.45452
	0	14.99946	6.076238	20.01456	1824.13278
	27	15.01437	6.088306	20.0736	1834.96951

Table S10 : Lattice parameters and cell volume of m-TNT in function of Pressure

Energetic materials	P (GPa)	a (Å)	b (Å)	c (Å)	β (°)	Volume (Å^3)
m-TNT Bowden etal.[27]	0	15	6.05	21	not provided	1828.8
	0.4	14.8	6	20.9	not provided	1773.936
	0.6	14.7	5.9	20.8	not provided	1737.36
	2	14.5	5.82	20.6	not provided	1609.344
	2.5	14.4	5.8	20.3	not provided	1591.056
	3	14.3	5.75	20.2	not provided	1554.48
	4.55	14.25	5.74	19.8	not provided	1499.616
	5	14.2	5.73	19.6	not provided	1481.328
	6	14.15	5.72	19.3	not provided	1435.608
	7.6	14.1	5.71	19.1	not provided	1417.32
	8	14.1	5.71	19	not provided	1408.176
	9	14	5.7	18.8	not provided	1389.888
	10	14	5.68	18.6	not provided	1371.6
m-TNT Stevens et al.[28]	0.2	14.847	6.074	21.035	111.07	1770.124
	0.5	14.709	5.998	20.886	111.14	1718.651
	1	14.499	5.904	20.854	111.14	1665.009
	1.5	14.431	5.855	20.722	111.01	1634.472
	2	14.327	5.816	20.569	111.15	1598.477
	2.9	14.279	5.798	20.479	111.09	1581.881
	3.1	14.272	5.766	20.181	111.06	1549.812
	4.2	14.233	5.738	19.95	111.19	1519.133
	4.7	14.196	5.732	19.721	111.29	1495.211
	5.6	14.122	5.731	19.406	111.86	1457.658
	5.9	14.137	5.724	19.383	111.59	1458.432
	6.1	14.118	5.724	19.396	111.59	1457.449
	6.8	14.052	5.731	19.303	112.08	1440.501
	7.1	14.064	5.727	19.177	112.16	1430.509
	7.7	14.072	5.727	19.059	112.05	1423.625

	7.9	14.028	5.721	19.073	112.1	1418.226
	8.1	14.046	5.716	19.014	112	1415.416
	8.7	14.018	5.713	18.924	111.94	1405.764
	9.6	14.044	5.701	18.761	112.01	1392.621
	10.2	14.052	5.679	18.637	112.11	1377.888
	11.5	14.085	5.662	18.522	112.13	1368.299
	13.6	13.956	5.63	18.477	112.33	1342.912
	14.5	13.897	5.618	18.498	112.54	1333.881
	15.1	13.805	5.581	18.606	112.71	1322.373
	17	13.874	5.555	18.414	112.51	1311.045
	20.3	13.967	5.549	18.128	112.76	1295.571
	23	14.13	5.455	16.469	90	1269.416
	24.3	14.288	5.451	15.693	90	1222.231
	26.6	14.441	5.459	15.431	90	1216.478

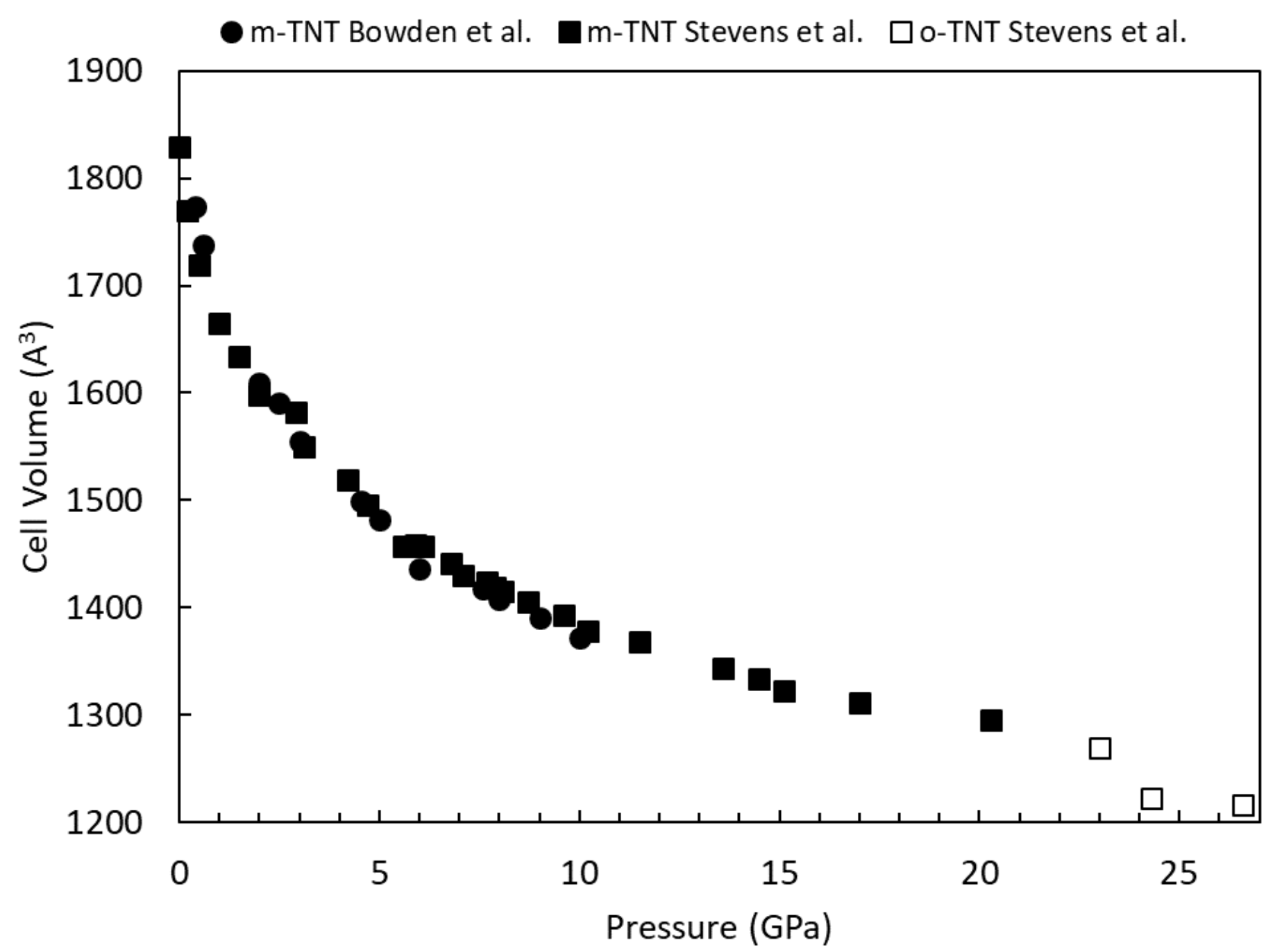

Figure S2 : Variation of Cell volume of m-TNT with pressure[27,28]. A polymorphic transformation to orthorhombic form is observed above 20.3 GPa[27].

3) HMX

Table S11 : Lattice parameters and cell volume of β-HMX in function of temperature

Energetic materials	T (°C)	a (Å)	b (Å)	c (Å)	β (°)	Volume (Å^3)
β-HMX Bolotina et al.[1]	-188	6.5227	10.809	7.3142	102.192	504.051
	-178	6.5233	10.8183	7.3159	102.211	504.617
	-158	6.5246	10.837	7.3195	102.25	505.756
	-138	6.5259	10.8559	7.3232	102.291	506.919
	-98	6.5289	10.8959	7.3311	102.378	509.396
	-78	6.5306	10.9173	7.3355	102.425	510.746
	-58	6.5325	10.94	7.3403	102.473	512.196
	-18	6.5369	10.9903	7.3514	102.575	515.471
	2	6.5395	11.0183	7.3578	102.63	517.336

	22	6.5424	11.0487	7.3649	102.686	519.378
β-HMX Deschamps et al.[4]	-150	6.525	10.84	7.3207	102.271	505.977
	-100	6.5288	10.8862	7.3306	102.365	508.91
	-100	6.5273	10.8834	7.3286	102.366	508.54
	-75	6.5206	10.9123	7.3395	102.467	509.93
	-50	6.5334	10.9419	7.3421	102.491	512.45
	-25	6.5254	10.9702	7.3503	102.582	513.53
	0	6.5289	10.9875	7.3453	102.616	514.2
	20	6.5245	11.024	7.3619	102.642	516.68
	30	6.5255	11.0369	7.364	102.67	517.45
β-HMX Yan et al.[5]	-100	6.525	10.884	7.331	102.369	508.553
	-60	6.529	10.930	7.340	102.467	511.451
	-20	6.533	10.980	7.350	102.567	514.565
	20	6.535	11.034	7.361	102.694	517.840
	60	6.540	11.092	7.373	102.793	521.541
	100	6.542	11.152	7.383	102.93	524.984
	120	6.546	11.184	7.388	102.985	526.960
	140	6.549	11.212	7.391	103.063	528.618
	150	6.5457	11.242	7.395	103.135	530.113

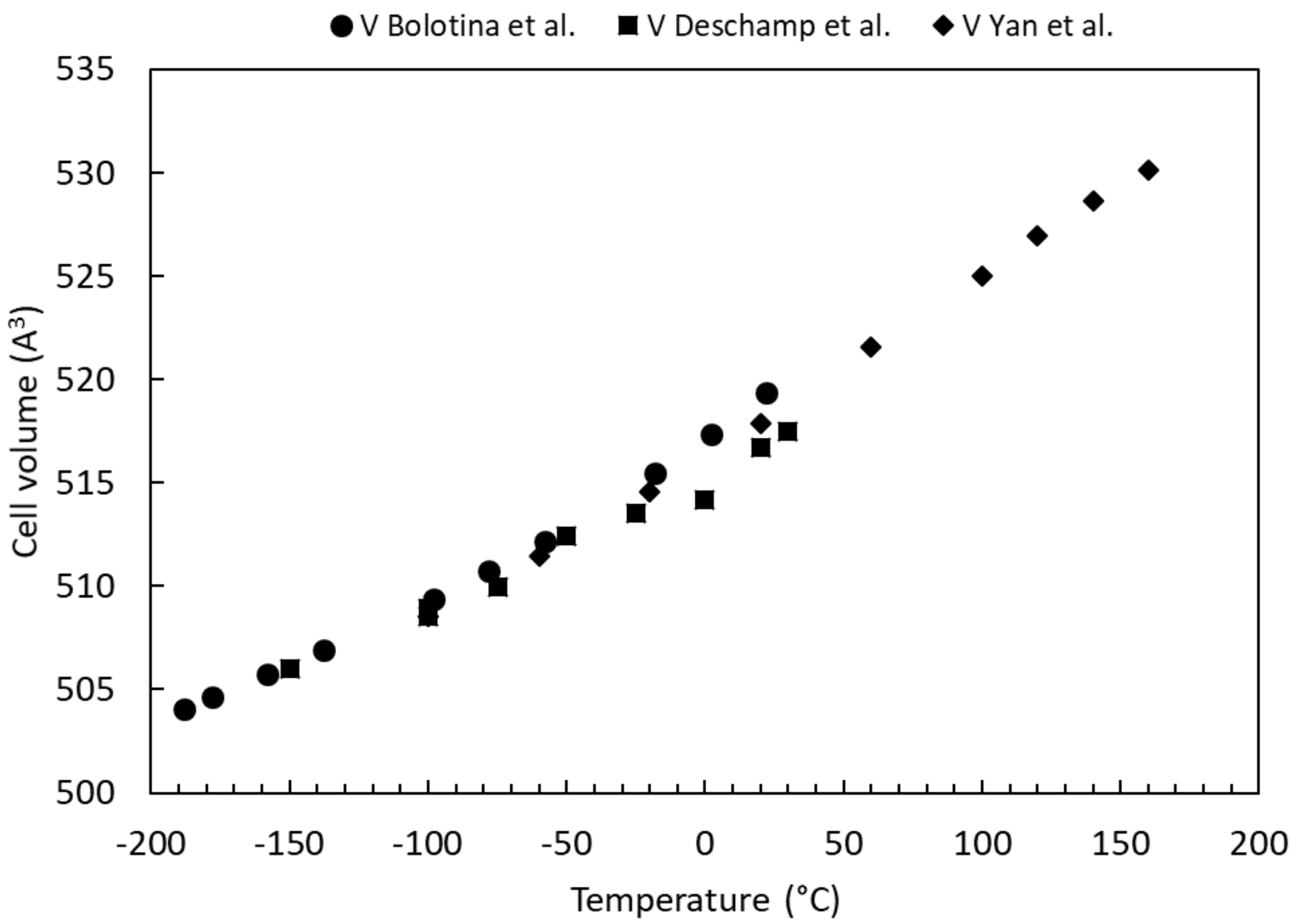

Figure S3 : Variation of Cell volume of β-HMX with temperature

Table S12 : Lattice parameters and cell volume of β-HMX in function of Pressure

Energetic materials	P (GPa)	a (Å)	b (Å)	c (Å)	β (°)	Volume (Å^3)
β-HMX Yoo et al.[29]	0	6.54	11.05	8.7	124.3	519.386915
	0.2	6.501	11.072	8.702	124.1	518.665432
	2.5	6.369	10.465	8.568	125.29	466.129919
	4.1	6.274	10.268	8.465	125.44	444.290893
	4.6	6.251	10.222	8.47	125.31	441.650258
	5.7	6.192	10.15	8.446	124.86	435.565731

	7	6.177	9.826	8.465	125.29	419.370969
	7.1	6.173	10.01	8.38	125.36	422.294462
	10.6	5.997	9.654	8.334	124.36	398.305201
	14.1	6.188	9.503	8.235	127.29	385.263687
	15.8	6.092	9.303	8.252	125.52	380.644882
	22.9	5.793	8.957	8.381	125.11	355.747164
	24.9	5.702	9.212	8.168	124.44	353.836643
	26	5.677	9.188	8.152	124.49	350.469196
	29.3	5.468	9.01	8.153	125	329.029819
	32.1	5.208	8.96	8.299	124.63	318.654153
	36.9	5.205	8.429	8.639	123.93	314.479175
	42.6	5.116	8.374	8.584	123.44	306.874265
β-HMX Sui et al.[30]	0	6.543	11.049	8.708	124.43	519.24
	1.5	6.481	10.618	8.628	125.08	485.82
	1.9	6.429	10.492	8.576	125.22	472.58
	3.2	6.36	10.307	8.526	125.52	454.86
	4.6	6.253	10.24	8.449	125.49	440.44
	8.5	6.118	9.854	8.363	125.33	411.32
	11.3	6.1	9.705	8.361	125.53	402.87
	12.2	6.099	9.556	8.404	125.54	398.56
	13.8	6.084	9.476	8.4	125.49	394.27
	15.8	5.992	9.427	8.308	124.9	384.84
	20.5	5.933	8.991	8.393	124.56	368.72
	21.6	5.902	8.914	8.405	124.45	364.6
	24	5.874	8.784	8.408	124.3	358.38
	29.3	5.833	8.702	8.359	124	351.81
	35.5	5.807	8.57	8.317	124.13	344.19
	38	5.765	8.486	8.242	123.84	336.53
β-HMX Gao et al.[31]	0.2	6.54	11.022	8.7012	124.731	515.469
	1	6.37	10.892	8.5699	124.1	492.362
	2	6.345	10.771	8.4963	124.174	480.396
	3.1	6.282	10.629	8.43	124.3	464.996
	4.2	6.252	10.548	8.376	124.5	455.218
	5.1	6.233	10.474	8.3691	124.49	450.333
	6.2	6.215	10.417	8.2724	124.88	439.355
	7.5	6.183	10.24	8.2993	124.902	430.948
	8.1	6.182	10.146	8.29	124.92	426.351
	9.1	6.126	10.083	8.2746	124.75	419.951
	10.6	6.146	10.037	8.2593	124.94	417.659
	11.6	6.125	9.978	8.257	124.93	413.721
	12.6	6.13	9.846	8.2578	125.06	407.972
	13.6	6.13	9.763	8.282	125.19	405.071
	14.6	6.109	9.672	8.3097	124.96	402.391

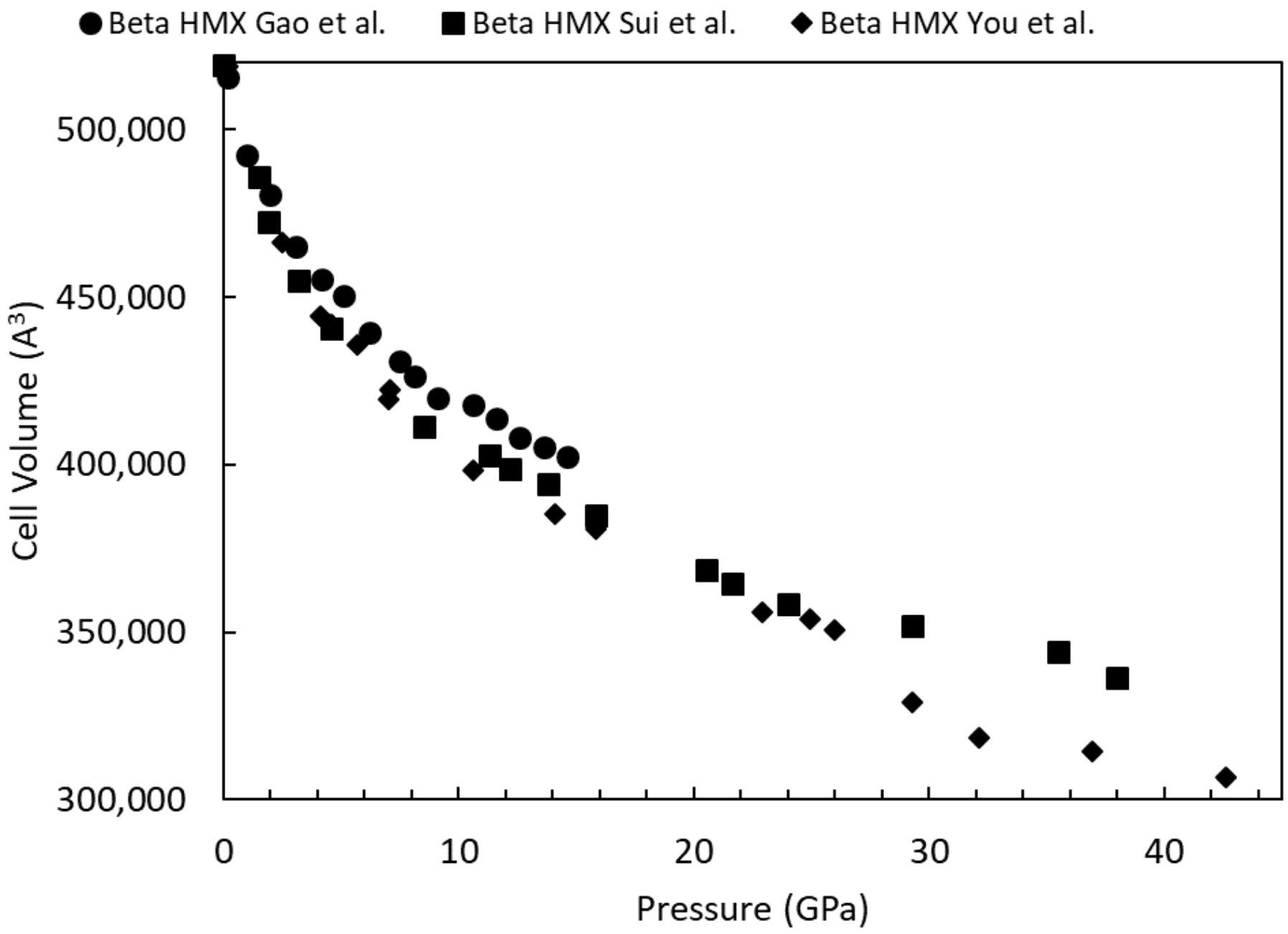

Figure S4 : Variation of Cell volume of β-HMX with pressure

4) PETN

Table S13 : Lattice parameters and cell volume of I-PETN in function of temperature

Energetic materials	T (°C)	a (Å)	c (Å)	Volume (Å^3)
I-PETN Bolotina et al.[1]	-263	9.2555	6.5901	564.54
	-238	9.2591	6.5948	565.38
	-203	9.2701	6.6067	567.75
	-173	9.2796	6.6203	570.08
	-153	9.2879	6.6289	571.84
	-133	9.2965	6.6359	573.51
	-113	9.3053	6.6438	575.28
	-93	9.3144	6.6521	577.12
	-73	9.3242	6.6609	579.1
	-53	9.3355	6.6708	581.37
	-33	9.3495	6.682	584.09
	-13	9.3604	6.6914	586.28
	7	9.3727	6.7023	588.78
	27	9.3863	6.7147	591.58
I-PETN Stankevich et al.[6]	-123	9.90016	6.64305	575.34712
	-113	9.91014	6.64305	576.52611
	-103	9.92012	6.6464	577.115605
	-93	9.92511	6.6531	577.7051
	-83	9.89517	6.6464	574.757625
	-73	9.90016	6.6531	576.52611
	-63	9.91014	6.66315	579.473585
	-53	9.92012	6.6598	578.88409
	-43	9.9301	6.6665	579.473585
	-33	9.93509	6.66985	581.24207
	-23	9.94008	6.6732	582.42106
	-13	9.95006	6.6799	584.189545
	-3	9.96004	6.6866	586.252778

	7	9.96004	6.6933	586.252778
	17	9.98	6.7	589.495
	27	9.98499	6.7134	591.263485
	37	9.98998	6.71675	592.442475
	47	9.99996	6.7201	593.916213
	57	10.00495	6.7268	595.38995
	67	10.00994	6.73685	597.158435
	77	10.01992	6.73685	598.337425
	87	10.02491	6.74355	598.92692
	97	10.031896	6.7536	601.2849

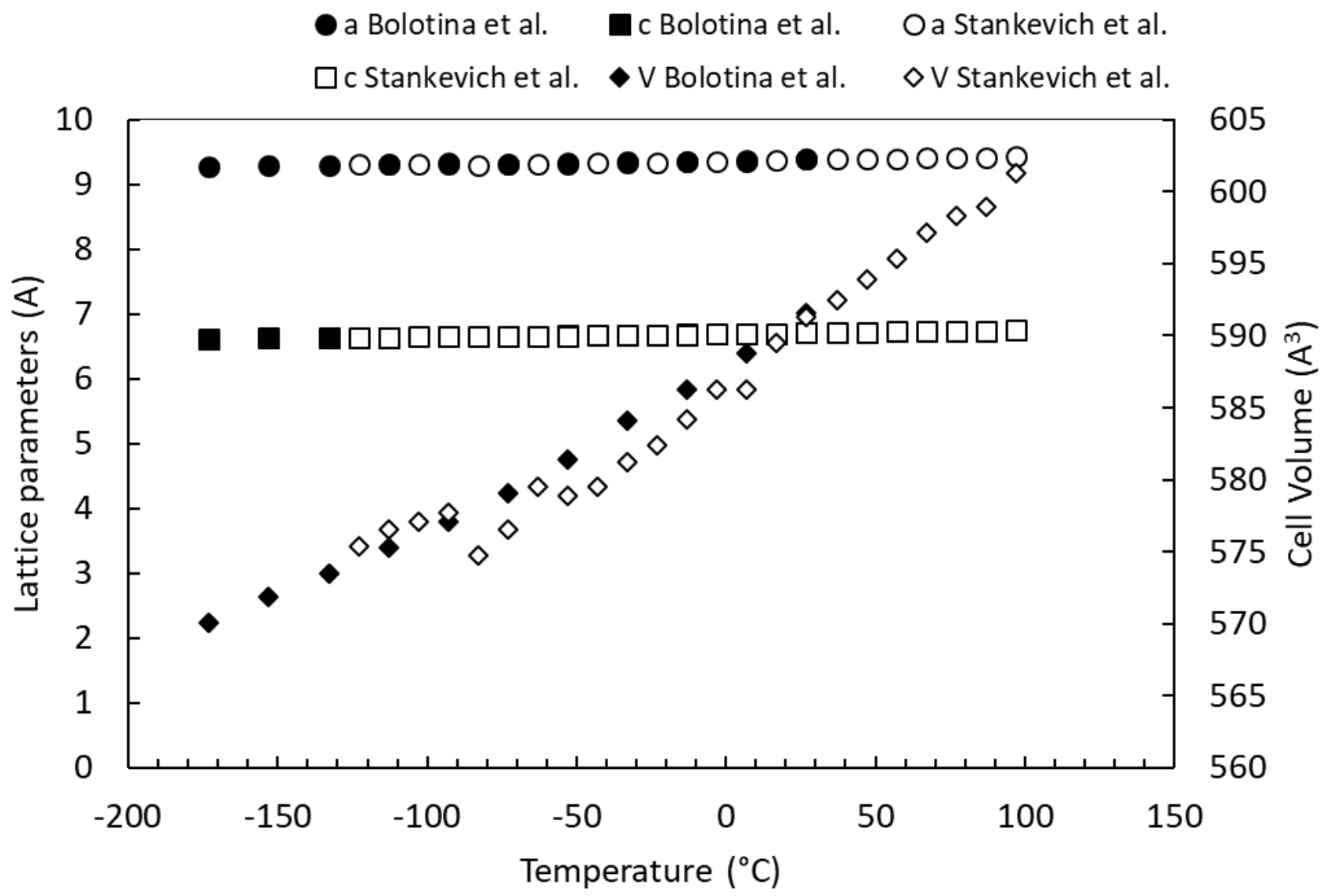

Figure S5 : : Evolution of a. c-axis and volume of PETN during the heating process[1,6]

Table S14 : Lattice parameters and cell volume of I-PETN in function of pressure

Energetic materials	P (GPa)	a (Å)	c (Å)	Volume (Å^3)
I-PETN Olinger et al.[32]	0	9.383	6.711	590.841064
	0.68	9.2216124	6.556647	557.576712
	0.86	9.1897102	6.4928925	548.300507
	1.28	9.1521782	6.4667196	541.683087
	1.71	9.110893	6.4412178	534.652079
	2.25	9.0039268	6.3868587	517.754024
	2.9	8.9748395	6.3036423	507.76881
	3.42	8.9485671	6.3023001	503.632923
	3.53	8.9269862	6.2915625	501.387727
	3.55	8.923233	6.2935758	501.092306
	3.65	8.9110351	6.2573364	496.897335
	3.95	8.9025904	6.2452566	495.006643
	4.9	8.8631818	6.2163993	488.330139
	4.98	8.8303413	6.194253	483.01257
	5	8.8265881	6.1915686	482.30356
	5.64	8.8059455	6.1794888	479.172103
	5.76	8.7937476	6.1855287	478.344925
	5.8	8.7815497	6.1613691	475.154384
	6.73	8.7421411	6.1372095	469.009637
	7.02	8.7130538	6.1150632	464.16474
	7.05	8.7271283	6.1385517	467.532534

	7.17	8.7139921	6.1224453	464.637413
	7.45	8.6952261	6.1096944	461.919544
	7.6	8.6924112	6.1304985	463.219394
	7.65	8.6924112	6.0956013	460.619693
	8.11	8.651126	6.1023123	456.720142
	8.32	8.6661388	6.0909036	457.429152
	8.4	8.6370515	6.0754683	453.23418
	9.04	8.6370515	6.0560064	451.816162
	9.16	8.622977	6.046611	449.570966
	10.12	8.5713705	6.0519798	444.607901
	10.45	8.5601109	6.0519798	443.426218

5) FOX-7

Table S15 : Lattice parameters and cell volume of α-FOX-7 in function of temperature

Energetic materials	T (°C)	a (Å)	b (Å)	c (Å)	B(°)	Volume ($Å^3$)	V/Z ($Å^3$)
α-FOX-7 Evers et al.[7] (same work than β-FOX-7)	-73	6.55	6.92	11.27	-	511	127.75
	27	6.61	6.93	11.32	-	518	129.5
	57	6.65	9.95	11.34	-	523	130.75
	97	6.7	6.95	11.35	-	527	131.75
α-FOX-7 Aree et al.[8]	-193	6.9129	6.4872	11.2465	90.4816	504.336	126.084
	-173	6.9146	6.4963	11.252	90.49	505.414	126.35
	-153	6.9165	6.5072	11.2575	90.4987	506.648	126.66
	-133	6.9184	6.5187	11.2646	90.5108	508.002	127.00
	-109	6.9209	6.5336	11.2726	90.5215	509.708	127.427
	-73	6.9254	6.5582	11.2866	90.5441	512.593	128.148
	-33	6.9303	6.5873	11.3017	90.5672	515.92	128.98
	7	6.935	6.619	11.3172	90.5886	519.463	129.865
	47	6.9413	6.6532	11.3353	90.608	523.46	130.865
	87	6.9484	6.6942	11.3547	90.618	528.12	132.03
α-FOX-7 McMon-agle et al[9] (same work than β-FOX-7)	1	6.92	6.6	11.34	-	517	129.25
	11	6.92218	6.60538	11.34732	-	518.5	129.62
	21	6.92436	6.61076	11.35464	-	519.5	129.87
	31	6.92654	6.61614	11.36196	-	521	130.25
	41	6.92872	6.62152	11.36928	-	522	130.5
	51	6.9309	6.6269	11.3766	-	524	131
	61	6.93308	6.63228	11.38392	-	525	131.25
	71	6.93526	6.63766	11.39124	-	526	131.5
	81	6.93744	6.64304	11.39856	-	527	131.75
	91	6.93962	6.64842	11.40588	-	528.5	132.12
	101	6.9418	6.6538	11.4132	-	530	132.5
α-FOX-7 Zhang et al.[33] (Performed between 1 and 1.28 GPa)	27	Not provided				499.2	124.8
	50	Not provided				500.1984	125.0496
	81	Not provided				501.696	125.424
	104	Not provided				504.192	126.048
	121	Not provided				505.1904	126.2976
	137	Not provided				506.1888	126.5472
	155	Not provided				509.184	127.296
	173	Not provided				511.1808	127.7952
	192	Not provided				513.1776	128.2944
	212	Not provided				514.176	128.544

Table S16 : Lattice parameters and cell volume of β-FOX-7 in function of temperature

Energetic materials	T (°C)	a (Å)	b (Å)	c (Å)	Volume (Å^3)	V/Z (A^3)
β-FOX-7 Evers et al.[7] (same work than α-FOX-7)	117	6.65	6.98	11.65	538	134.5
	127	6.65	6.98	11.67	541.5	135.37
	150	6.68	6.98	11.68	543	135.75
β-FOX-7 McMon-agle et al[9] (same work than α-FOX-7)	107	6.96	6.64	11.68	540	135
	117	6.96112	6.64578	11.689095	541	135.25
	127	6.96224	6.65156	11.69819	542	135.5
	137	6.96336	6.65734	11.707285	543	135.75
	147	6.96448	6.66312	11.71638	544	136
	157	6.9656	6.6689	11.725475	545	136.25
	167	6.96672	6.67468	11.73457	546	136.5
	177	6.96784	6.68046	11.743665	548	137
	187	6.96896	6.68624	11.75276	550	137.5

Table S17 : Lattice parameters and cell volume of γ-FOX-7 in function of temperature

Energetic materials	T (°C)	a (Å)	b (Å)	c (Å)	β (°)	Volume (Å^3)	V/Z (A^3)
γ-FOX-7 McMonagl e et al[9]	171	6.96	6.6	12.08	Not pro-vided	552	138
	187	6.955152	6.599968	12.104232	Not pro-vided	554	138.5
	201	6.95091	6.59994	12.125435	Not pro-vided	556	139
γ-FOX-7 Crawford et al.[10]	-73	13.354	6.895	12.049	111.102	1035	129.375
	22	13.603	6.924	12.047	111.12	1058.4	132.3
	54	13.744	6.922	12.024	111.7	1062	132.75
	74	13.792	6.921	12.022	111.5	1067	133.375
	94	13.842	6.931	11.992	111.5	1070	133.75
	114	13.892	6.941	11.992	111.4	1076	134.5
	134	13.942	6.941	12.002	111.3	1082	135.25
	154	13.994	6.952	11.993	111.2	1087	135.875
	174	14.073	6.942	12.013	111.2	1093	136.625
	184	14.123	6.932	12.032	111	1099	137.375
	220	14.223	6.942	12.072	110.9	1113	139.125

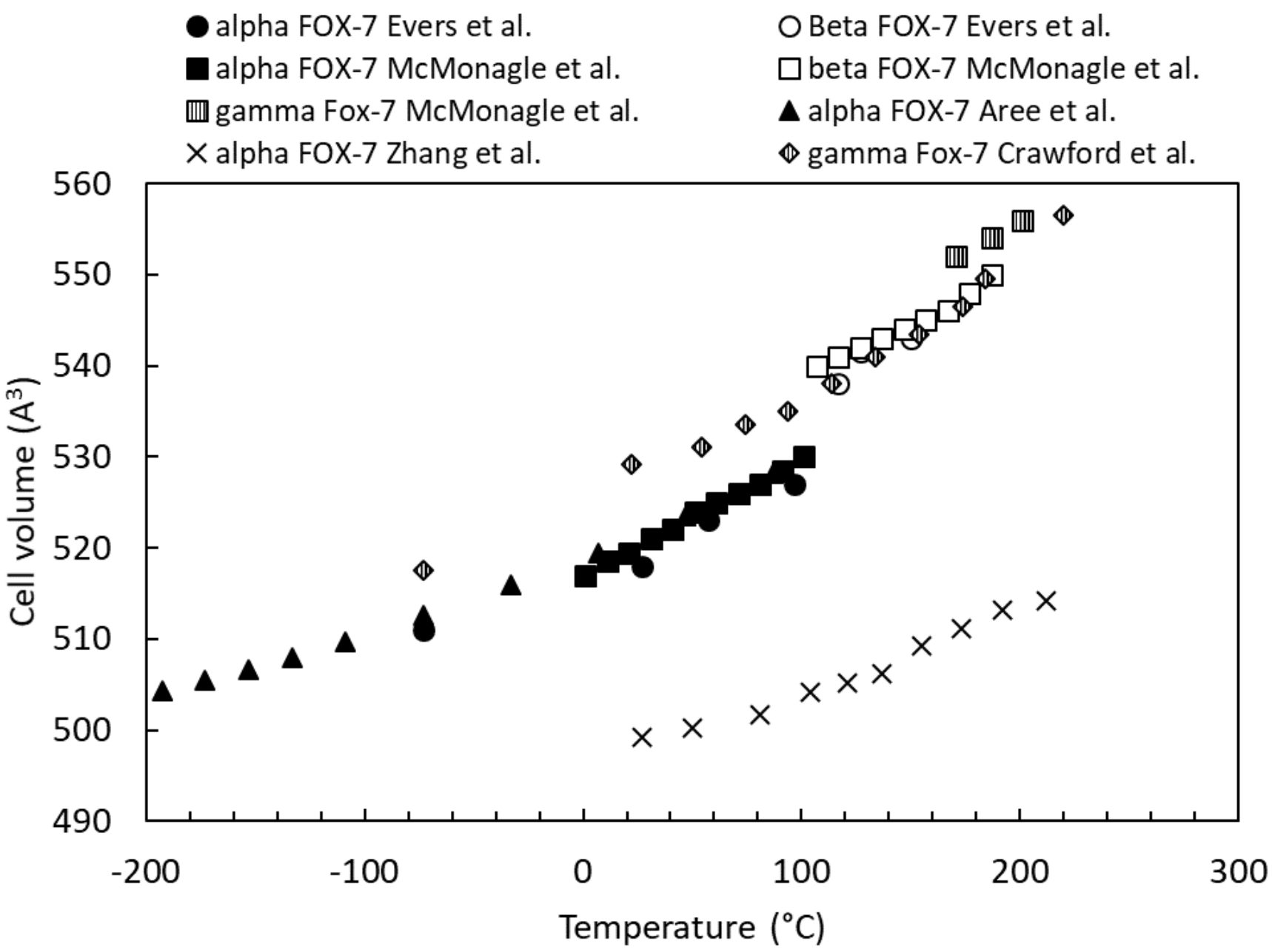

Figure S6 : Evolution of volume of FOX-7 during the heating process[1,6]. The volume of γ-FOX-7 is scaled to the same Z=4 as for α-FOX-7 and β-FOX-7.

Table S18 : Lattice parameters and cell volume of α-FOX-7 in function of pressure

Energetic materials	P (GPa)	a (Å)	b (Å)	c (Å)	β (Å)	Volume (Å^3)	V/Z (Å^3)
α-FOX-7 Zhang et al.[33]	0	Not provided				516.76	129,19
	0.8	Not provided				490.92	122,730
	1.43	Not provided				479.55	119,888
	2.1	Not provided				465.08	116,271
	2.47	Not provided				454.75	113,687
	3.2	Not provided				449.58	112,395
	4.18	Not provided				444.41	111,103
	4.88	Not provided				439.25	109,811
	5.69	Not provided				434.08	108,519
	6.76	Not provided				418.58	104,643
α-FOX-7 Hunter et al.[34]	0	6.94	6.628	11.3287	90.595	521.07	130.267
	0.09	6.9255	6.5759	11.305	90.547	514.82	128.705
	0.38	6.9014	6.5052	11.2701	90.471	505.96	126.49
	0.47	6.8956	6.5014	11.266	90.483	505.05	126.262
	0.68	6.8735	6.4349	11.2264	90.395	496.54	124.135
	1.33	6.8371	6.3428	11.1698	90.318	484.39	121.097
	1.34	6.8335	6.3271	11.163	90.321	482.64	120.66
	1.73	6.8146	6.2842	11.1294	90.267	476.6	119.15
	1.89	4.8046	6.2531	11.1121	90.263	472.81	118.202
	2.2	6.7905	6.2243	11.0877	90.224	468.63	117.157
	2.36	6.7835	6.2058	11.0722	90.198	466.1	116.525
	2.47	6.781	6.1955	11.0697	90.202	465.06	116.265
	2.58	6.7783	6.1865	11.0614	90.188	463.84	115.96
	2.64	6.767	6.1641	11.0516	90.184	460.99	115.247
	2.79	6.7622	6.1562	11.0438	90.147	459.75	114.937
	3.3	6.7482	6.117	11.0092	90.102	454.44	113.61
	3.63	6.7321	6.0788	10.9849	90.113	449.54	112.38
	4.14	6.7291	6.0629	10.9555	90.142	446.96	111.74
α-FOX-7	0	6.93	6.62	11.23	89.48	514.6	128.65

Peiris et al.[35]	0.21	6.898	6.54	11.22	89.48	506.5	126.625
	0.64	6.86	6.4	11.2	89.48	492	123
	1.15	6.84	6.35	11.16	89.48	484.5	121.125
	1.3	6.82	6.3	11.13	89.48	478.4	119.6
	1.33	6.82	6.34	11.08	89.48	478.5	119.625
	1.88	6.81	6.23	11.06	89.48	469.2	117.3
	2.14	6.78	6.23	11.02	89.48	465.9	116.475
	2.37	6.79	6.25	11	89.48	466.1	116.525
	2.92	6.77	6.18	10.99	89.48	459.7	114.925
	2.98	6.76	6.14	11.01	89.48	456.1	114.025
	3.17	6.76	6.15	10.93	89.48	455	113.75
	3.57	6.76	6.13	10.88	89.48	451.6	112.9
	3.75	6.75	6.1	11	89.48	452	113
	3.86	6.77	6.09	10.92	89.48	450.8	112.7
α-FOX-7 Dreger et al.[36]	0	6.948	6.6364	11.3413	90.569	522.92	130.729
	0.5	6.8931	6.5058	11.2567	90.48	504.79	126.197
	1	6.8358	6.3591	11.163	90.314	485.24	121.310
	1.3	6.822	6.3249	11.1378	90.263	480.57	120.143
	1.6	6.8011	6.2779	11.1042	90.249	474.11	118.526
	2	6.7982	6.2473	11.1067	90.215	471.70	117.925
	2.3	6.7789	6.2	11.0632	90.2	464.97	116.243
	3	6.7562	6.1435	11.0371	90.166	458.11	114.527
	3.3	6.7472	6.1083	11.0236	90.141	454.32	113.581
	4.27	6.7111	6.0339	10.9621	90.086	443.90	110.974

Table S19 : Lattice parameters and cell volume of ε-FOX-7 in function of pressure

Energetic materials	P (GPa)	a (Å)	b (Å)	c (Å)	α (°)	β (°)	γ (°)	Volume (A^3)	V/Z (A^3)
ε-FOX-7 Dreger et al.[36]	6	6.0745	6.6924	6.6972	119.505	93.913	110.042	213.04	106.52
	7	5.9859	6.6716	6.6854	119.463	94.11	110.145	208.53	104.26
	8.5	5.8965	6.6394	6.6545	119.395	94.308	110.262	203.13	101.56
	9.7	5.8695	6.6197	6.6343	119.409	94.338	110.317	200.80	100.4
	11.3	5.7268	6.603	6.6195	119.381	94.429	110.739	194.07	97.035
	12.8	5.6947	6.5793	6.5961	119.356	94.461	110.881	191.34	95.67

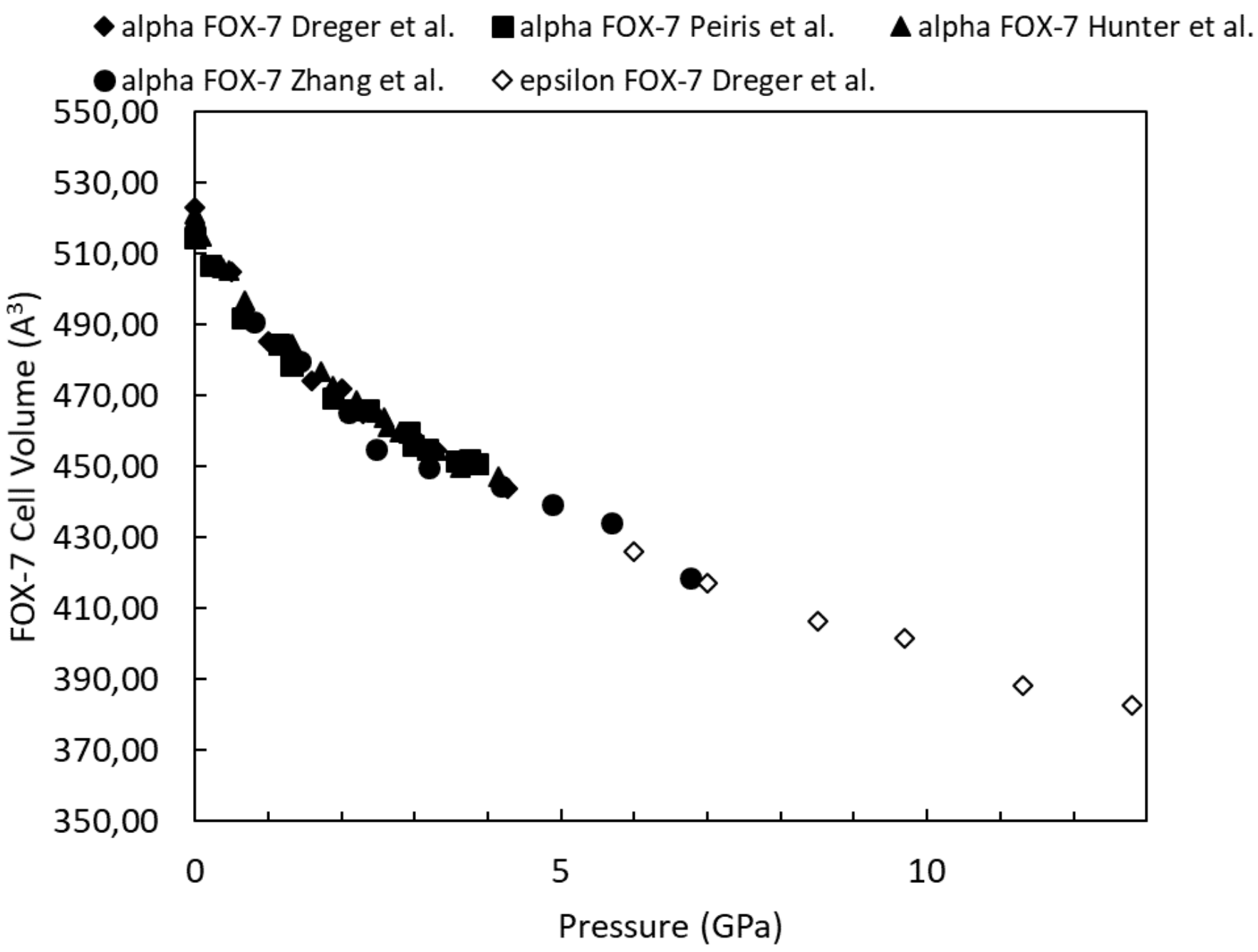

Figure S7 : Variation of Cell volume of α-FOX-7 and ε-FOX-7 with pressure[33–36]. The volume of ε-FOX-7 is scaled to the same Z=4 as for α-FOX-7.

6) CL-20

Table S20 : Lattice parameters and cell volume of ε-CL-20 in function of temperature

Energetic materials	T (°C)	a (Å)	b (Å)	c (Å)	β (°)	Volume ($Å^3$)	V/Z (A^3)
ε-CL-20 Bolotina et al.[1,12]	-173	8.791	12.481	13.285	106.551	1397.2	349.3
	-123	8.8	12.499	13.299	106.622	1401.7	350.425
	-73	8.821	12.537	13.333	106.74	1412	353
	-23	8.841	12.562	13.358	106.82	1420	355
	25	8.863	12.593	13.395	106.92	1430.2	357.55
ε-CL-20 Liang et al.[12]	25	8.8665	12.5975	13.3939	106.9081	1431.37	357.84
	65	8.8818	12.6233	13.4199	106.9803	1439.03	359.75
	100	8.9018	12.6547	13.4475	107.0459	1448.77	362.19
	105	8.9064	12.6615	13.4501	107.043	1450.14	362.53
	110	8.9093	12.6657	13.4527	107.0565	1451.26	362.81
	115	8.9113	12.6685	13.4588	107.0614	1452.54	363.13
ε-CL-20 Pu et al.[13]	30	8.80774	12.49322	13.31907	106.60636	1404.92	356.03
	40	8.812166	12.499498	13.325763	106.627724	1407.06	356.57
	50	8.816592	12.505776	13.332456	106.649088	1409.19	357.12
	60	8.821018	12.512054	13.339149	106.670452	1411.33	357.66
	70	8.825444	12.518332	13.345842	106.691816	1412.75	358.02
	80	8.82987	12.52461	13.352535	106.71318	1414.89	358.56
	90	8.834296	12.530888	13.359228	106.734544	1417.02	359.10
	100	8.838722	12.537166	13.365921	106.755908	1418.45	359.46
	110	8.843148	12.543444	13.372614	106.777272	1420.58	360.00
	120	8.847574	12.549722	13.379307	106.798636	1422.01	360.36
	130	8.852	12.556	13.386	106.82	1424.15	360.90

Table S21 : Lattice parameters and cell volume of γ-CL-20 in function of temperature

Energetic materials	T (°C)	a (Å)	b (Å)	c (Å)	β (°)	Volume ($Å^3$)	V/Z ($Å^3$)

γ-CL-20 Bolotina et al.[1.12]	-173	13.0342	8.1773	14.7465	108.566	1489.95	372.48
	-123	13.0698	8.1737	14.7718	108.696	1494.78	373.69
	-73	13.1156	8.1713	14.8059	108.841	1501.75	375.43
	-23	13.167	8.1676	14.8436	109.001	1509.34	377.33
	25	13.2272	8.1692	14.892	109.164	1519.99	379.99
γ-CL-20 Pu et al.[13]	30	13.058997	8.182255	14.72724	108.51498	1492.30	379.72
	40	13.072228	8.180621	14.734678	108.536814	1493.82	380.10
	50	13.0788435	8.179804	14.749554	108.558648	1495.34	380.49
	60	13.0920745	8.178987	14.756992	108.602316	1496.86	380.88
	70	13.09869	8.17817	14.771868	108.645984	1499.14	381.46
	80	13.111921	8.177353	14.779306	108.689652	1500.66	381.84
	90	13.125152	8.176536	14.786744	108.73332	1502.18	382.23
	100	13.138383	8.175719	14.786744	108.766071	1503.70	382.62
	110	13.151614	8.174902	14.80162	108.798822	1505.22	383.00
	120	13.1582295	8.174085	14.809058	108.84249	1506.74	383.39
	130	13.1714605	8.174085	14.816496	108.897075	1509.78	384.16
	140	13.178076	8.173268	14.823934	108.95166	1511.29	384.55
	150	13.191307	8.172451	14.83881	109.006245	1513.57	385.13
	160	13.204538	8.171634	14.846248	109.06083	1514.33	385.32
	170	13.217769	8.170817	14.861124	109.115415	1517.37	386.09
	180	13.231	8.17	14.876	109.17	1518.89	386.48

Table S22 : Lattice parameters and cell volume of α-CL-20 in function of temperature

Energetic materials	T (°C)	a (Å)	b (Å)	c (Å)	Volume (Å^3)	V/Z (A^3)
α-CL-20 Pu et al.[13]	30	9.449566	13.1373288	23.396073	2904.68	363.08
	40	9.454305	13.13997	23.4078535	2907.63	363.45
	50	9.459044	13.146573	23.419634	2912.05	364.00
	60	9.4618874	13.159779	23.4314145	2915.00	364.37
	70	9.4647308	13.1637408	23.4549755	2917.95	364.74
	80	9.468522	13.166382	23.466756	2925.32	365.66
	90	9.4704176	13.172985	23.4785365	2931.22	366.40
	100	9.4723132	13.179588	23.5020975	2935.64	366.95
	110	9.4742088	13.186191	23.513878	2938.59	367.32
	120	9.4761044	13.192794	23.537439	2943.02	367.87
	130	9.478	13.206	23.561	2948.92	368.61

Table S23 : : Lattice parameters and cell volume of β-CL-20 in function of temperature

Energetic materials	T (°C)	a (Å)	b (Å)	c (Å)	Volume (Å^3)	V/Z (A^3)
β-CL-20 Pu et al.[13]	30	9.62762	12.9903928	11.5849305	1448.38	362.09
	40	9.632458	12.992994	11.590755	1450.58	362.64
	50	9.637296	12.9942946	11.5965795	1452.05	363.01
	60	9.646972	12.9955952	11.602404	1454.25	363.56
	70	9.65181	12.9968958	11.614053	1456.45	364.11
	80	9.656648	12.999497	11.6198775	1458.65	364.66
	90	9.661486	13.0007976	11.625702	1460.11	365.02
	100	9.666324	13.0020982	11.6315265	1462.31	365.57
	110	9.671162	13.0033988	11.6431755	1463.78	365.94
	120	9.676	13.006	11.649	1465.98	366.49

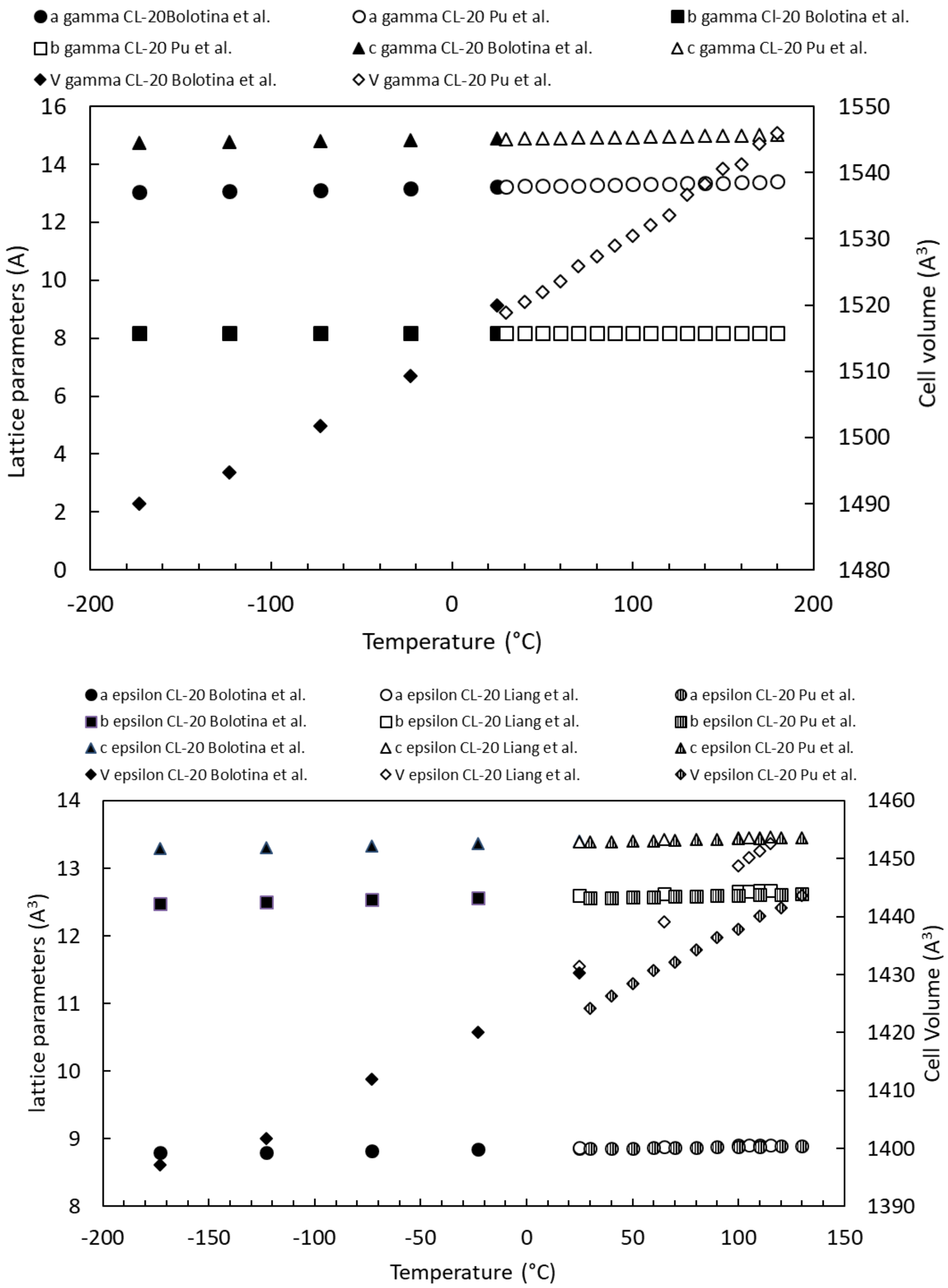

Figure S8 : Evolution of lattice parameters and volume of ε-CL-20 (up) and γ-CL-20 (down) during the heating process[1.24.25].

Table S24 : Lattice parameters and cell volume of ε-CL-20 in function of pressure

Energetic materials	P (GPa)	a (Å)	b (Å)	c (Å)	β (°)	Volume $(Å^3)$	V/Z (A^3)
ε-CL-20 Konar et al.[37]	0	8.8628	12.5959	13.3795	106.917	1429	357.25
	0.22	8.8138	12.5104	13.3113	106.742	1405.5	351.37
	0.43	8.7739	12.4427	13.2529	106.608	1386.50	346.62
	0.71	8.7295	12.3543	13.1809	106.405	1363.60	340.9
	1.01	8.6906	12.2765	13.1115	106.229	1343.10	335.77
	1.31	8.6571	12.2049	13.0487	106.081	1324.80	331.2
	1.63	8.6238	12.1386	12.9916	105.903	1307.90	326.97
	2.04	8.5912	12.0631	12.9278	105.713	1289.70	322.42
	2.4	8.5607	11.9942	12.8663	105.527	1272.90	318.22

	2.83	8.5322	11.9252	12.8078	105.366	1256.60	314.15
	3.34	8.5002	11.8539	12.7417	105.146	1239.30	309.82
	4.44	8.4483	11.7239	12.621	104.796	1208.60	302.15
	5.2	8.4158	11.6417	12.5467	104.601	1189.60	297.4
	5.75	8.3936	11.5923	12.501	104.493	1177.70	294.42
	6.24	8.3735	11.5491	12.4552	104.326	1167.00	291.75
	6.7	8.3604	11.509	12.4219	104.253	1158.40	289.6
	7.18	8.3441	11.4717	12.3905	104.183	1149.90	287.47
ε-CL-20 Gump et al.[37.38] (T = ambiant temperature)	0	8.84	12.58	13.38	107.02	1422.34	355.58
	0	8.89	12.58	13.28	106.45	1423.80	355.95
	0	8.83	12.63	13.33	106.81	1423.24	355.81
	0.91	8.65	12.31	13.27	106.17	1356.22	339.05
	0.96	8.69	12.31	13.26	106.4	1360.74	340.18
	1.37	8.57	12.24	13.07	106.41	1315.25	328.81
	1.59	8.63	12.15	13.09	106.22	1317.77	329.44
	1.64	8.58	12.17	13.08	106.08	1311.34	327.83
	1.92	8.61	12.07	13	106.05	1298.01	324.50
	2.2	8.55	12.05	13.01	106.02	1288.43	322.10
	2.47	8.58	11.97	12.98	105.92	1280.97	320.24
	2.53	8.58	11.97	12.87	106.08	1271.18	317.79
	2.58	8.53	11.98	13.03	105.64	1282.60	320.65
	2.97	8.6	11.9	12.89	105.8	1269.78	317.44
	3.22	8.54	11.82	12.77	105.56	1240.19	310.04
	3.3	8.5	11.87	12.89	105.01	1256.52	314.13
	3.51	8.56	11.86	12.85	105.56	1256.69	314.17
	3.99	8.52	11.75	12.72	105.35	1228.08	307.02
	4.13	8.52	11.8	12.77	105.15	1238.83	309.70
	4.26	8.52	11.78	12.81	105.18	1240.91	310.22
	4.55	8.45	11.78	12.69	104.97	1220.81	305.20
	4.74	8.49	11.65	12.57	104.81	1202.13	300.53
	4.79	8.48	11.76	12.71	104.92	1224.62	306.15
	5.02	8.46	11.73	12.66	104.76	1213.53	303.38
	5.57	8.45	11.73	12.64	104.69	1210.39	302.59
ε-CL-20 Gump et al.[37.38] (T = 75°C)	0	8.85	12.6	13.37	104.69	1427.02	356.75
	0	8.85	12.62	13.4	106.91	1430.20	357.55
	0.71	8.73	12.28	13.14	106.98	1352.61	338.15
	0.85	8.76	12.28	13.15	106.2	1356.63	339.15
	1.15	8.6	12.2	13.06	106.29	1318.42	329.605
	1.5	8.63	12.13	13.07	105.66	1313.73	328.43
	1.62	8.61	12.09	12.98	106.22	1303.88	325.97
	2.05	8.62	12.04	13	105.21	1295.53	323.88
	2.06	8.57	12.02	12.98	106.04	1289.12	322.28
	2.58	8.54	11.97	12.92	105.33	1272.97	318.24
	2.64	8.58	11.95	12.91	105.41	1275.39	318.84
	3.13	8.52	11.93	12.87	105.38	1263.18	315.79
	3.54	8.47	11.84	12.83	105.01	1244.16	311.04
	4.09	8.48	11.74	12.76	104.82	1229.72	307.43
	4.25	8.49	11.75	12.72	104.57	1226.81	306.70
	5.1	8.46	11.59	12.61	104.79	1197.18	299.29

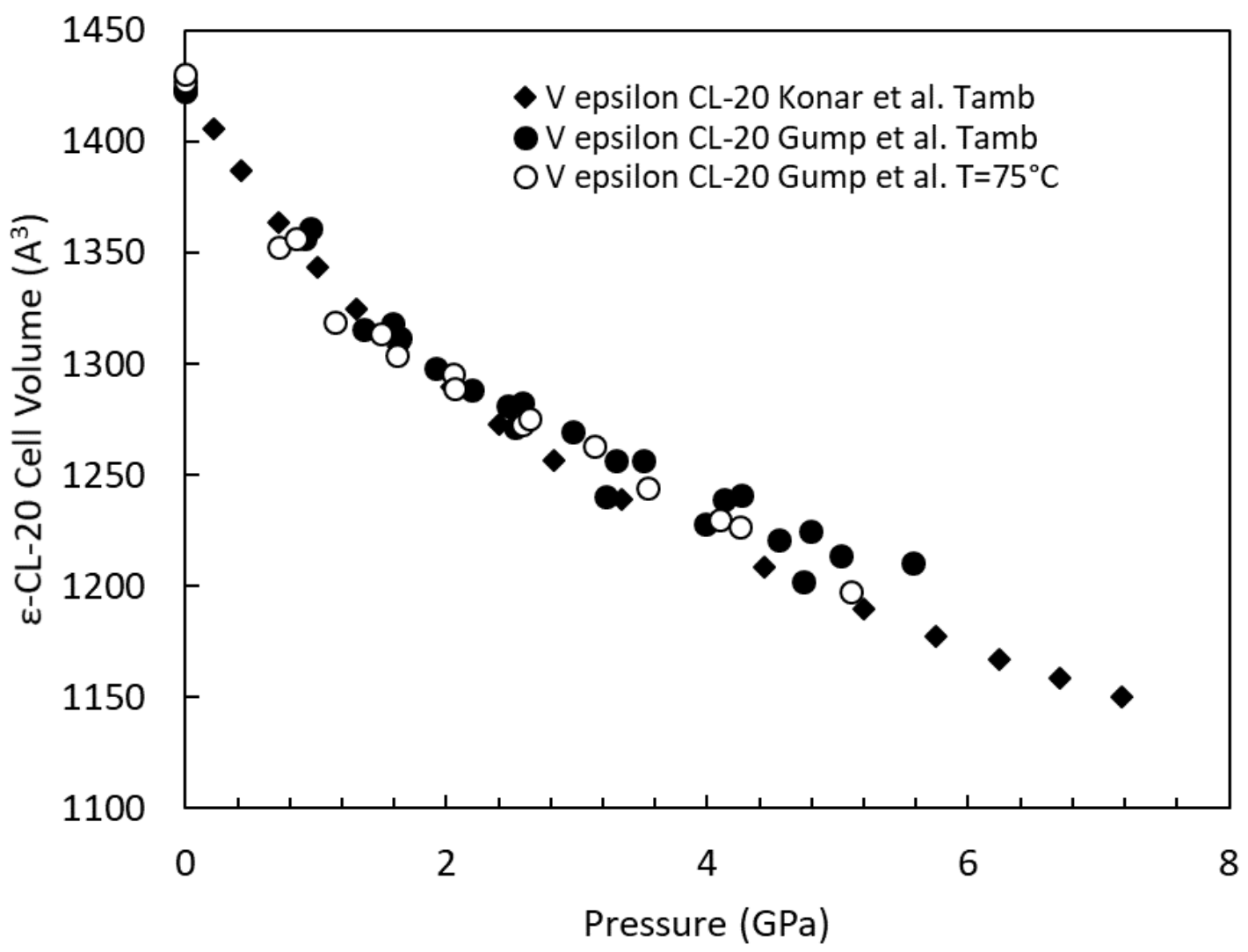

Figure S9 : Variation of Cell volume of ε-CL-20 with pressure

7) TATB

Table S25 : Lattice parameters and cell volume of TATB in function of temperature

Energetic materials	T (°C)	a (Å)	b (Å)	c (Å)	α (°)	β (°)	γ (°)	Volume (Å^3)
TATB[14]	-59	9.007	9.023	6.713	108.75	92.03	119.98	434.5
	-45	9.013	9.032	6.727	108.78	91.96	119.99	436.2
	-36	9.007	9.029	6.734	108.74	92.04	119.97	436.3
	-26	9.007	9.033	6.748	108.75	91.98	119.96	437.4
	-13	9.007	9.030	6.762	108.64	92.04	119.93	438.7
	-1	8.989	9.033	6.776	108.69	92.07	119.90	438.8
	11	9.015	9.033	6.797	108.66	91.88	119.96	441.6
	23	9.017	9.031	6.815	108.63	91.90	119.95	442.9
	35	9.018	9.047	6.856	108.39	92.04	119.95	447
	44	9.014	9.045	6.587	108.43	92.04	119.94	446.7
	54	9.016	9.046	6.872	108.49	91.97	119.95	447.7
	65	9.016	9.049	6.891	108.40	91.99	119.93	449.5
	76	9.015	9.050	6.923	108.41	91.97	119.89	451.9
	83	9.019	9.054	6.943	108.41	91.99	119.90	453.4
	94	9.014	9.052	6.963	108.39	91.96	119.87	454.7
	104	9.016	9.054	6.980	108.39	91.90	119.86	456.2
TATB[15]	30	9.016	9.033	6.827	108.68	91.77	119.97	443.7
	60	9.022	9.037	6.86	108.64	91.73	119.97	446.66
	90	9.02	9.034	6.888	108.61	91.69	119.98	448.4
	120	9.025	9.041	6.925	108.58	91.66	119.97	451.63
	150	9.029	9.042	6.959	108.55	91.61	119.98	454.29
	180	9.029	9.047	6.993	108.55	91.56	119.98	456.91
	210	9.035	9.051	7.033	108.52	91.53	119.99	460.08
	240	9.036	9.049	7.068	108.53	91.5	119.97	462.43

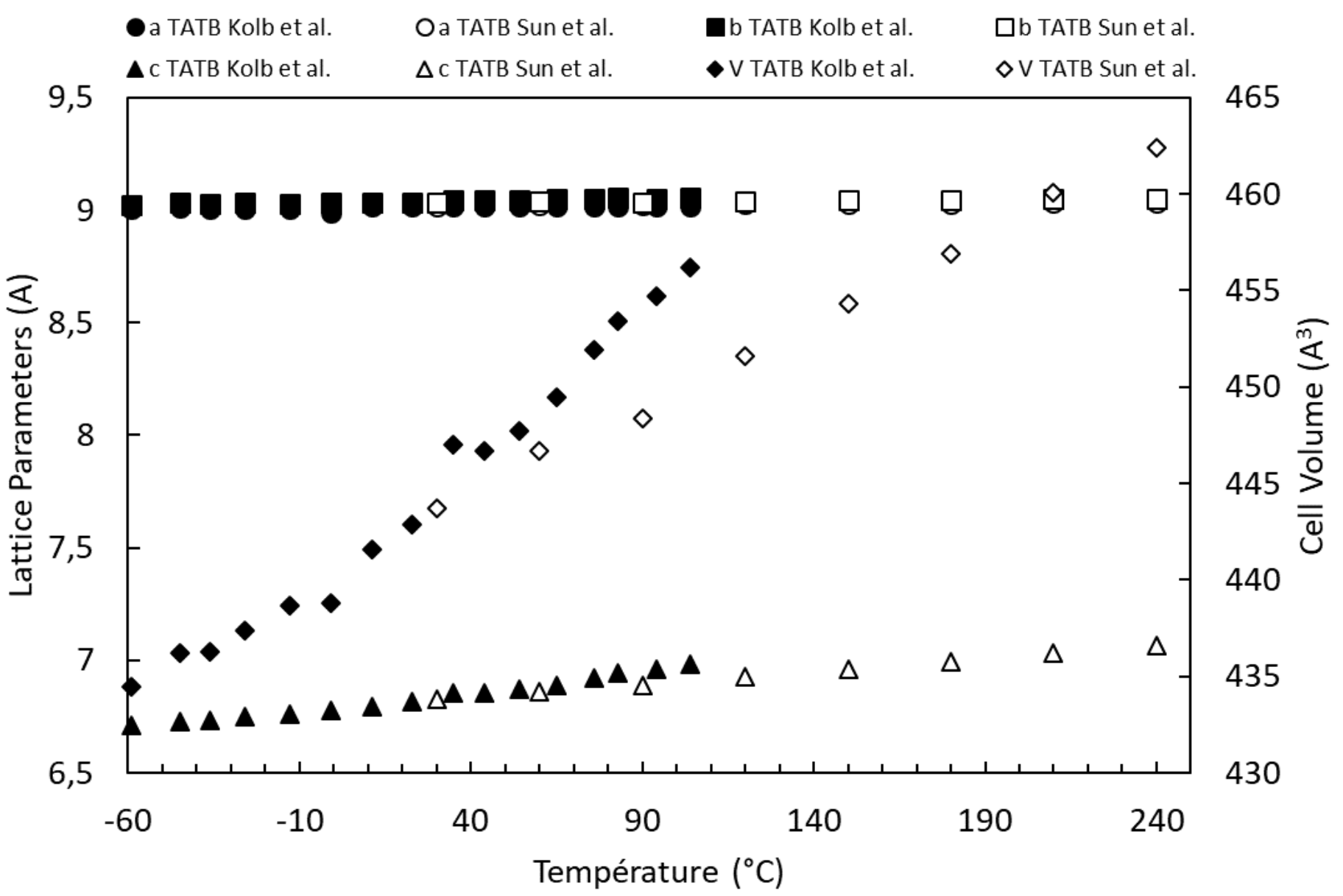

Figure S10 : Evolution of lattice parameters and volume of TATB during the heating process[29.30].

Table S26 : Lattice parameters and cell volume of TATB in function of Pressure

Energetic materials	P (GPa)	a (Å)	b (Å)	c (Å)	α (°)	β (°)	γ (°)	Volume (Å^3)
TATB Stevens et al.[40]	0	Not provided						442.5
	0.56	8.967	9.082	6.624	110.5	93	118.9	425.154
	0.85	Not provided						420.286
	1.2	Not provided						415.817
	1.66	Not provided						410.197
	1.95	Not provided						404.799
	2.42	Not provided						400.639
	3.29	Not provided						390.904
	4.95	Not provided						379.665
	8.18	Not provided						359.929
	8.53	Not provided						355.991
	9.53	Not provided						351.477
	10.17	Not provided						348.203
	11.64	Not provided						341.034
	13.22	8.507	8.762	5.93	114.2	96.5	116.6	335.370
TATB Sun et al.[41]	0	9.028	9.0871	6.8434	Not provided			450.00
	1	8.98	9.02	6.6	Not provided			427.50
	2	8.95	8.96	6.5	Not provided			414.00
	2.5	8.92	8.96	6.4	Not provided			405.00
	4	8.88	8.92	6.3	Not provided			391.50
	5	8.82	8.8	6.25	Not provided			373.50
	7.5	8.78	8.79	6.05	Not provided			364.50
	8.5	8.76	8.76	6	Not provided			360.00
	10.5	8.68	8.7	5.85	Not provided			346.50
	12	8.66	8.68	5.8	Not provided			335.25
	14.5	8.6	8.62	5.7	Not provided			326.25
	16.5	8.58	8.6	5.6	Not provided			319.50
TATB	0	9.04	9.03	6.81	108.5	91.7	120	443.80
	2	8.88	8.89	6.41	110	92.2	120.1	397.20

Plisson et al.[42]	4.4	8.78	8.81	6.17	110.8	92	120.2	371.40
	7.6	8.76	8.72	5.994	111.5	91.8	119.9	355.70
	9.9	8.71	8.68	5.902	111.8	91.6	119.9	346.30
	13.3	8.67	8.63	5.796	112.3	91.3	119.9	335.50
	17.3	8.62	8.57	5.684	112.7	91.1	119.9	323.90
	21	8.59	8.51	5.598	113	90.9	119.9	315.00
	25.8	8.55	8.46	5.509	113.3	90.6	119.9	305.90
	28.3	8.53	8.43	5.458	113.5	90.5	119.9	301.00
	30.8	8.52	8.4	5.422	113.7	90.4	119.9	297.00
	36	8.48	8.36	5.342	114	90.3	120	289.10
	41.2	8.4	8.3	5.301	114	90	119.8	283.10
	46.1	8.35	8.27	5.249	114.1	89.9	119.8	277.60
	49.5	8.3	8.24	5.21	114	90	119.5	274.40
	55.1	8.25	8.2	5.17	114	89.9	119.5	269.20
	58.5	8.24	8.17	5.13	114.2	89.8	119.6	265.30
	61.8	8.23	8.15	5.1	114.3	89.8	119.5	262.40
	66	8.23	8.12	5.07	114.6	89.6	119.6	259.10
TATB Steele et al.[43]	0	9.01	9.028	6.812	108.59	91.82	119.97	442.49
	0.64	8.98	9.021	6.846	109	91.55	120.3	437
	0.97	8.97	8.986	6.781	107.4	91.65	119.67	436.9
	1.81	8.977	8.947	6.413	109.2	92.5	118.0	416.9
	1.9	8.98	8.99	6.404	111.68	91.67	119.3	404.7
	3.2	8.909	8.926	6.068	109.07	90.13	119.89	387.3
TATB Steele et al.[44]	0	9.04	9.05	6.62	109.17	91.1	119.89	430.07
	2.5	8.9	8.9	6.4	110.2	91.7	119.89	399.599
	5	8.78	8.8	6.1	111	92	119.8	368.388
	10	8.66	8.7	5.8	112	91.7	119.8	338.700
	15	8.57	8.58	5.75	112.5	91.5	119.8	326.387
	20	8.5	8.52	5.65	113	91.5	119.8	314.157
	25	8.45	8.46	5.5	113.2	91.5	119.8	301.209
	30	8.4	8.42	5.4	113.6	91.5	119.8	291.280
	34	8.35	8.38	5.3	113.8	91.5	119.8	282.188

8) LLM-105

Table S27 : Lattice parameters and cell volume of LLM-105 in function of Temperature

Energetic materials	T (°C)	a (Å)	b (Å)	c (Å)	β (°)	Volume (Å^3)
LLM-105 Xu et al.[16]	-268	5.70	15.6	8.38	Not provided	732
	-253	5.70	15.6	8.38	Not provided	732
	-233	5.70	15.6	8.38	Not provided	732
	-213	5.702	15.62	8.384	Not provided	733
	-193	5.703	15.63	8.388	Not provided	734
	-173	5.704	15.64	8.39	Not provided	735
	-153	5.705	15.67	8.392	Not provided	737
	-133	5.707	15.68	8.398	Not provided	738
	-113	5.71	15.72	8.40	Not provided	740
	-93	5.713	15.75	8.405	Not provided	742
	-73	5.715	15.77	8.41	Not provided	744
	-53	5.720	15.8	8.415	Not provided	746
	-33	5.724	1.83	8.425	Not provided	748
	-13	5.727	15.86	8.43	Not provided	752
	7	5.735	15.88	8.44	Not provided	754
	27	5.736	15.9	8.442	Not provided	756
	37	5.737	15.92	8.444	Not provided	757
	47	5.744	15.94	8.448	Not provided	758
	67	5.747	15.97	8.452	Not provided	760
	87	5.750	16	8.46	Not provided	763
LLM-105 Gump et al.[17]	25	5.71	15.83	8.42	100.98	747.7
	26	5.70	15.78	8.45	100.70	746.7
	28	5.72	15.87	8.40	101.01	749.4
	40	5.71	15.82	8.42	100.89	746.1
	45	5.72	15.87	8.44	100.97	752.1
	50	5.72	15.91	8.43	100.88	753.4
	60	5.72	15.86	8.42	100.77	750.2
	65	5.73	15.90	8.44	100.88	754.6
	70	5.72	15.94	8.44	100.96	756
	80	5.72	15.89	8.43	100.90	751.8
	85	5.73	15.92	8.48	100.89	759.3
	90	5.73	15.96	8.44	101.03	758
	100	5.72	15.91	8.43	100.90	754.1
	105	5.74	15.98	8.48	101.18	762.7
	110	5.74	15.99	8.42	101.32	757.4
	120	5.73	15.99	8.44	101.19	758.7
	125	5.76	16.06	8.38	100.96	761
	130	5.75	16.03	8.44	101.18	762.3
	140	5.73	16.01	8.46	101.20	761.3
	145	5.74	16.06	8.51	101.42	769.1
	150	5.75	16.10	8.46	101.46	767.6
	160	5.74	16.06	8.46	101.28	765

	165	5.77	16.17	8.39	101.23	767.2
	170	5.75	16.15	8.48	101.43	771.3
	180	5.75	16.08	8.47	101.37	768.4
	185	5.76	16.11	8.51	101.14	774.7
	190	5.76	16.14	8.52	101.08	776.9
	200	5.76	16.14	8.48	101.40	772.4
	205	5.78	16.18	8.48	101.38	777.6
	210	5.77	16.20	8.49	101.49	777.7
	220	5.77	16.18	8.47	101.53	774.3
	225	5.78	16.21	8.48	101.23	779.1
	230	5.78	16.23	8.51	101.47	782.2
	240	5.77	16.22	8.48	101.27	778.6
LLM-105 Li et al.[18]	30	5.7188	15.8667	8.4258	101.15	750.111
	40	5.7198	15.8788	8.4286	101.162	751.029
	50	5.7214	15.8878	8.4303	101.173	751.772
	60	5.7231	15.8988	8.433	101.186	752.739
	70	5.7249	15.9082	8.435	101.198	753.57
	80	5.7265	15.9164	8.4368	101.206	754.311
	90	5.7281	15.9258	8.4391	101.214	755.151
	100	5.7296	15.9345	8.441	101.222	755.913
	110	5.7311	15.9441	8.4433	101.233	756.747
	120	5.7329	15.9557	8.4457	101.242	757.729
	130	5.7348	15.9672	8.4485	101.253	758.749
	140	5.7369	15.9791	8.4514	101.263	759.821
	150	5.7396	15.9915	8.4548	101.274	761.04
	160	5.7418	16.0047	8.4584	101.285	762.268
	170	5.7443	16.0172	8.4613	101.295	763.421
	180	5.7465	16.0294	8.4646	101.306	764.567
	190	5.7483	16.0412	8.4666	101.317	765.521
	200	5.751	16.0541	8.4702	101.327	766.796
	190	5.7484	16.0413	8.4668	101.316	765.558
	180	5.7461	16.0282	8.4644	101.308	764.531
	170	5.7438	16.0167	8.4616	101.296	763.402
	160	5.7413	16.0054	8.4586	101.285	762.233
	150	5.7392	15.9924	8.455	101.276	761.023
	140	5.7364	15.9801	8.4517	101.264	759.866
	130	5.7345	15.9679	8.4488	101.254	758.753
	120	5.7323	15.9571	8.4459	101.245	757.758
	110	5.7307	15.9458	8.4436	101.236	756.778
	100	5.7294	15.9359	8.4414	101.224	755.962
	90	5.7278	15.9253	8.4395	101.212	755.143
	80	5.7264	15.9148	8.4371	101.204	754.295
	70	5.7251	15.9068	8.4354	101.196	753.628
	60	5.7233	15.8969	8.4334	101.184	752.698
	50	5.7213	15.8857	8.4306	101.172	751.757
	40	5.7197	15.8769	8.4283	101.163	750.952
	30	5.7187	15.8652	8.4259	101.151	750.068

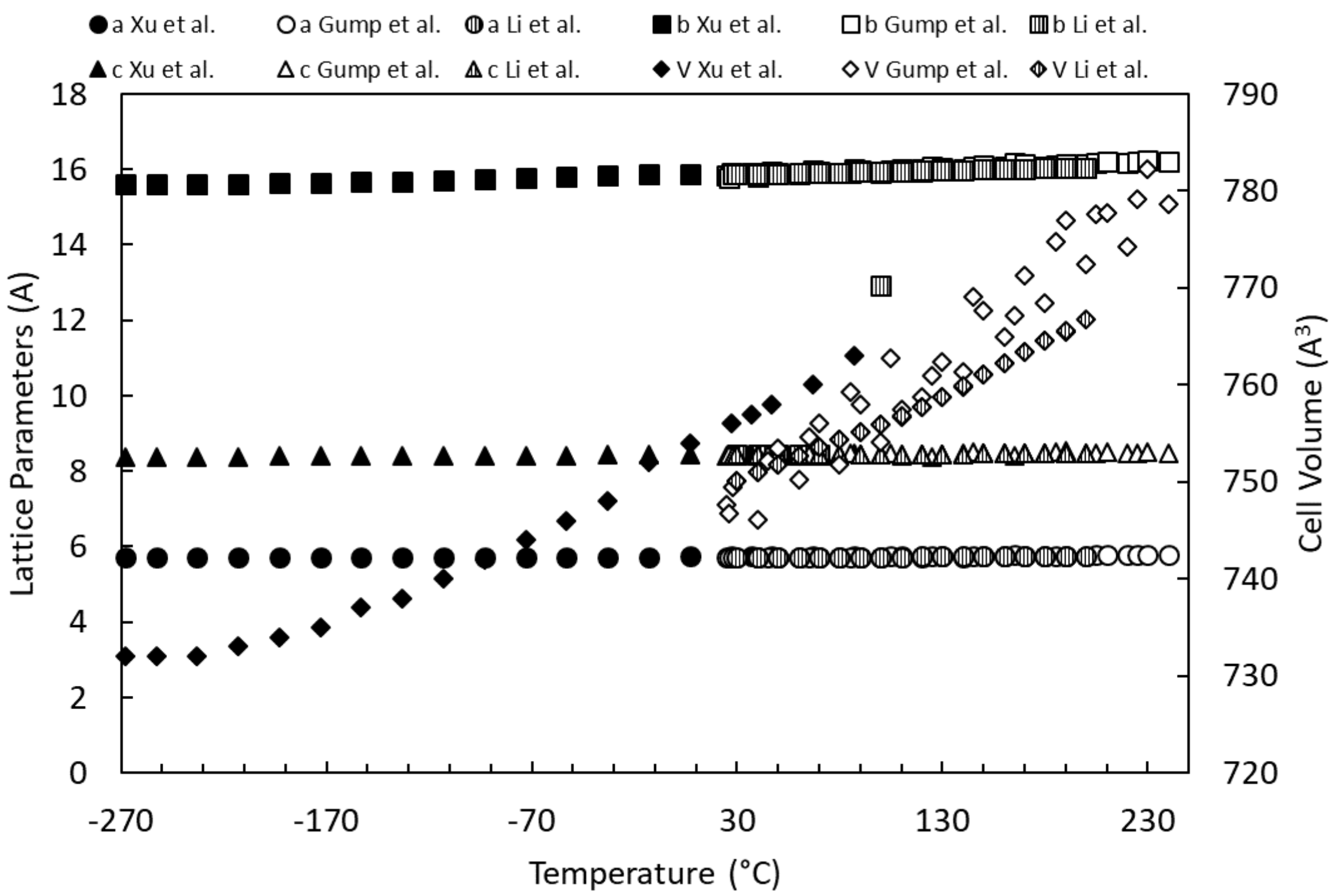

Figure S11 : Evolution of lattice parameters a. b and c and volume of LLM-105 during the heating process[16–18]

Table S28 : Lattice parameters and cell volume of LLM-105 in function of Pressure

Energetic materials	P (GPa)	a (Å)	b (Å)	c (Å)	β (°)	Volume (Å^3)
LLM-105 Xu et al.[16]	0	5.642	15.956	8.455	100.93	747.38
	6	5.5	14.6	8	not provided	620
	9	5.45	14.5	8	not provided	585
	12	5.4	14.2	8	not provided	575
	17	5.35	13.9	7.8	not provided	555
	25.8	5.3	13.3	7.7	not provided	510
LLM-105 Stavrou et al.[45]	0	5.723	15.87	8.424	not provided	750.08
	1	5.7	15.7	8.3	not provided	720
	2	5.65	15.5	8.25	not provided	700
	2.5	5.6	15.2	8.2	not provided	665
	3.8	5.45	14.9	8.1	not provided	630
	7.6	54	14.25	8.1	not provided	600
	11	5.4	14.2	8.05	not provided	575
	12.5	5.35	13.9	8	not provided	560
	16	5.3	13.7	7.8	not provided	550
	19.5	5.15	13.62	7.74	not provided	542
LLM-105 Gump et al.[17] T= Ambiant temperature	0	5.72	15.82	8.42	101.15	747.3
	0	5.71	15.85	8.39	101.11	745.8
	0	5.71	15.83	8.41	101.08	745.5
	0.1	5.71	15.76	8.41	101.24	742
	0.2	5.69	15.67	8.39	101.13	733.2
	0.3	5.69	15.69	8.36	101.16	732.5
	0.6	5.66	15.43	8.32	101.09	712.9
	0.8	5.66	15.45	8.3	101.13	711.9
	1	5.64	15.3	8.29	101.06	702.2
	1.1	5.65	15.29	8.29	101.03	702.9

	1.4	5.63	15.29	8.31	101.17	701.8
	1.5	5.59	15.27	8.24	100.62	691.3
	1.8	5.59	15.21	8.18	100.5	683.4
	1.9	5.61	15.14	8.26	101.09	687.7
	2	5.58	15.21	8.26	100.77	688.5
	2.1	5.59	15.14	8.19	100.64	680.50
	2.2	5.6	15.08	8.2	100.99	679.20
	2.3	5.57	15.16	8.2	100.48	680.20
	2.5	5.56	14.97	8.2	100.08	672.20
	2.7	5.59	14.98	8.14	100.78	669.30
	2.9	5.56	14.98	8.13	100.58	665.40
	3.1	5.58	14.94	8.04	100.48	658.50
	3.3	5.53	14.92	8.19	100.12	665.20
	3.6	5.56	14.7	8.12	100.87	652.80
	3.7	5.51	14.89	8.19	100.23	661.40
	3.8	5.56	14.82	8.07	100.47	654.10
	4	5.52	14.71	8.09	100.46	645.40
	4.3	5.51	14.84	8.17	100.19	657.00
	4.4	5.53	14.66	8.05	100.42	641.30
	4.8	5.51	14.85	8.05	100.22	647.60
	4.9	5.53	14.57	8.07	100.63	638.90
	5.4	5.5	14.48	8.04	100.51	629.50
LLM-105 Gump et al.[17] T= 100°C	0	5.72	15.91	8.43	100.9	754.10
	0.5	5.64	15.18	8.26	100.96	693.30
	0.6	5.65	15.34	8.28	101.11	704.00
	0.8	5.62	15.13	8.24	101.15	686.60
	1.1	5.56	14.99	8.19	100.79	670.40
	1.1	5.62	15.09	8.22	101.2	682.90
	1.3	5.62	15.14	8.24	101.09	688.20
	1.4	5.57	15.03	8.27	101.2	679.20
	1.4	5.61	15.02	8.21	101.16	678.10
	1.6	5.56	14.93	8.17	100.79	666.00
	1.8	5.61	14.98	8.21	101.11	676.50
	1.9	5.58	14.88	8.19	101.01	667.40
	1.9	5.6	14.91	8.2	101.23	670.90
	2	5.55	14.83	8.1	100.79	654.60
	2.2	5.57	14.85	8.17	100.83	664.2
	2.3	5.56	14.81	8.16	100.83	660.7
	2.5	5.54	14.82	8.11	100.54	653.8
	2.7	5.52	14.77	8.11	100.56	650
	3	5.54	14.71	8.14	100.77	652.2
	3.2	5.52	14.68	8.06	100.4	642.2
	3.5	5.51	14.7	8.11	100.61	645.8
	3.6	5.51	14.61	8.05	100.44	637.3
	3.7	5.52	14.74	8.04	100.48	643.3
	4.2	5.5	14.64	8.04	100.36	635.8
	4.2	5.49	14.63	8.08	100.21	638.3
	4.7	5.49	14.53	8.02	100.38	628.8
	4.8	5.5	14.48	8.09	100.63	633.3
	5.5	5.51	14.4	8.1	100.8	631.2
LLM-105 Gump et al.[17]	0	5.75	16.08	8.47	101.37	768.4
	0.4	5.69	15.38	8.43	100.75	724.5
	0.4	5.68	15.58	8.33	101.27	723.5

T= 180°C	0.9	5.64	15.25	8.27	101.22	697.5
	1	5.65	15.19	8.35	100.28	705.2
	1.5	5.62	15.04	8.3	101.15	688.8
	1.6	5.62	15.06	8.23	101.15	682.9
	1.9	5.6	14.98	8.2	101.02	675.3
	2.3	5.59	14.86	8.17	101.1	665.8
	2.4	5.57	14.89	8.18	101.08	665.3
	3.3	5.57	14.72	8.15	101.1	655.6
	4.1	5.57	14.64	8.14	101.12	650.8
	5	5.54	14.57	8.1	100.97	642.3

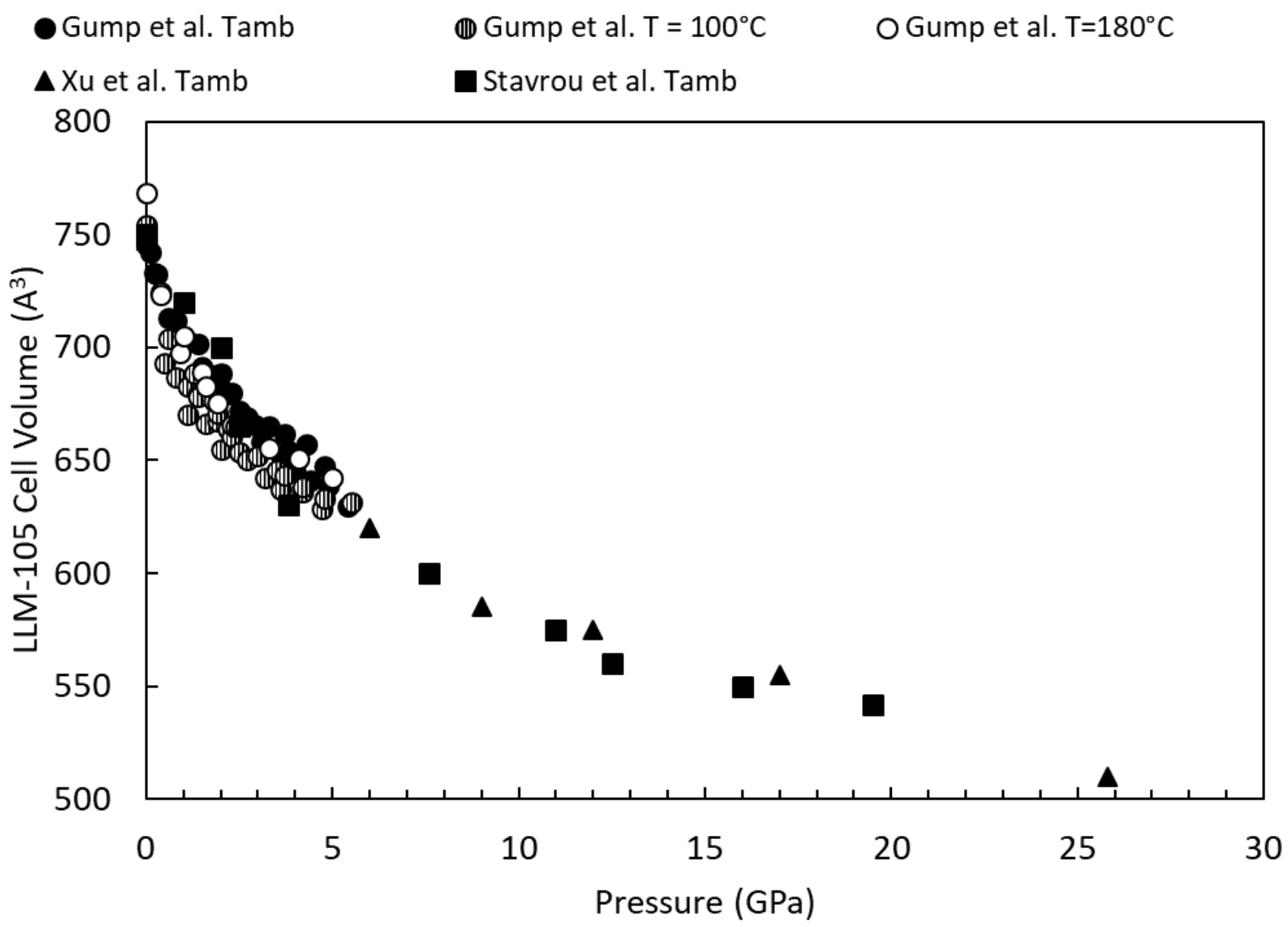

Figure S12 : Variation of Cell volume of LLM-105 with pressure[36.37.39]

9) HNS

Table S29 : Lattice parameters and cell volume of Trans-HNS in function of Temperature

Energetic materials	T (°C)	a (Å)	b (Å)	c (Å)	β (°)	Volume (Å^3)
Trans-HNS[19]	30	22.3472	5.5753	14.6698	110.05	1716.98
	60	22.3927	5.5851	14.6740	110.00	1724.51
	90	22.4400	5.5961	14.6798	109.96	1732.67
	120	22.4915	5.6076	14.6864	109.92	1741.49
	150	22.5382	5.6174	14.6889	109.88	1748.94
	180	22.5931	5.6300	14.6947	109.83	1758.29
	210	22.6501	5.6431	14.7015	109.79	1768.10
	240	22.7062	5.6542	14.7025	109.74	1776.61
Trans-HNS [20]	20	22.326	5.5538882	14.622999	not provided	1720
	30	22.348326	5.5594588	14.637666	not provided	1720

	40	22.370652	5.5650294	14.637666	not provided	1720
	50	22.392978	5.5706	14.667	not provided	1725
	60	22.415304	5.5594588	14.667	not provided	1735
	70	22.43763	5.5706	14.652333	not provided	1725
	80	22.459956	5.5817412	14.622999	not provided	1730
	90	22.459956	5.5817412	14.622999	not provided	1740
	100	22.459956	5.5817412	14.667	not provided	1740
	110	22.54926	5.5928824	14.681667	not provided	1740
	120	22.526934	5.5928824	14.652333	not provided	1745
	130	22.504608	5.5928824	14.681667	not provided	1750
	140	22.482282	5.598453	14.637666	not provided	1750
	150	22.54926	5.5928824	14.711001	not provided	1750
	160	22.593912	5.6095942	14.711001	not provided	1750
	170	22.616238	5.6430178	14.725668	not provided	1760
	180	22.54926	5.6151648	14.740335	not provided	1760
	190	22.593912	5.626306	14.725668	not provided	1765
	200	22.616238	5.6374472	14.711001	not provided	1770
	210	22.638564	5.6374472	14.711001	not provided	1780
	220	22.638564	5.654159	14.711001	not provided	1780
	230	22.705542	5.6653002	14.667	not provided	1790
	240	22.727868	5.6708708	14.711001	not provided	1790
	250	22.77252	5.682012	14.740335	not provided	1800
	260	22.817172	5.6875826	14.725668	not provided	1795
	270	22.77252	5.6875826	14.769669	not provided	1800
	290	22.839498	5.709865	14.711001	not provided	1810

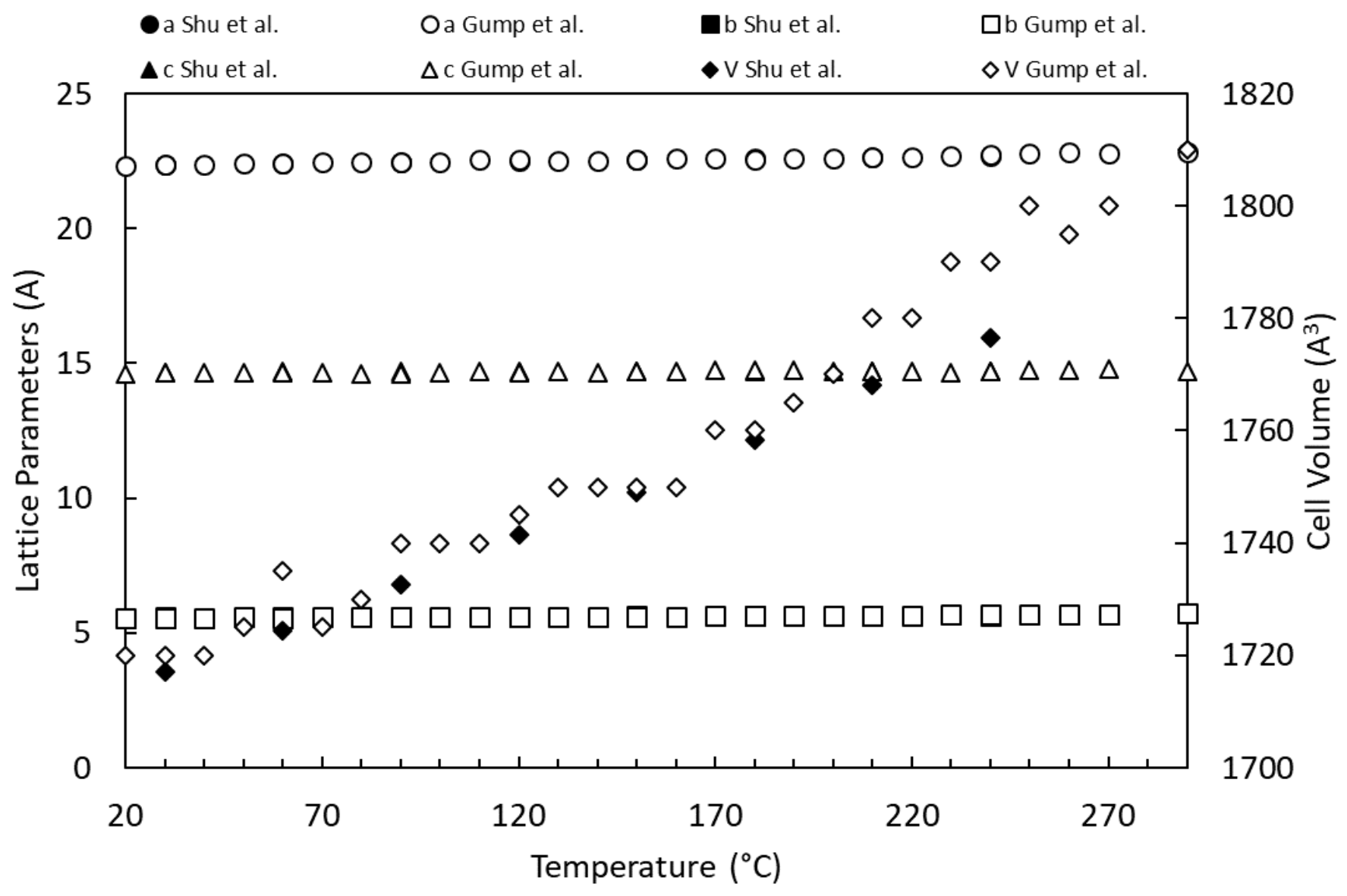

Figure S13 : Evolution of lattice parameters a. b and c and volume of trans-HNS during the heating process[40.41]

Table S30 : Lattice parameters and cell volume of Trans-HNS in function of Pressure

Energetic materials	P (GPa)	a (Å)	b (Å)	c (Å)	β (°)	Volume ($Å^3$)
Trans-HNS Gump et al.[17.20]	0	22.083	5.554	14.634	108.45	1702.59051
	0.5	21.751755	5.44292	14.634	not provided	1634.4864
	1	21.530925	5.30407	14.634	not provided	1566.3828

	1.8	21.42051	5.231868	14.634	not provided	1515.3051
	2.4	21.089265	5.231868	14.634	not provided	1481.2533
	3	20.75802	5.10968	14.634	not provided	1447.2015
	3.8	20.272194	5.05414	14.78034	not provided	1413.1497
	4	20.205945	5.05414	14.78034	not provided	1396.1238
	4.6	20.09553	5.02637	14.809608	not provided	1379.0979
	5	19.830534	4.97083	14.85351	not provided	1362.072
Trans-HNS Deng et al.[46]	0	22.122	5.5747	14.6672	108.487	1715.46809
	0.5	21.688	5.3559	14.757	108.44	1626.14224
	1	21.434	5.2569	14.764	108.7	1575.73572
	1.5	21.3	5.1648	14.7908	108.639	1541.79775
	2	21.024	5.046	14.8201	108.75	1488.7839
	2.5	20.995	4.9882	14.8276	108.678	1471.07024
	3	21	4.9406	14.773	108.67	1452.0815
	3.8	20.771	4.8819	14.771	108.66	1419.07416
	4.3	20.684	4.8371	14.739	108.64	1397.29366
	5.2	20.526	4.781	14.686	108.65	1365.52952
	6.2	20.499	4.7253	14.628	108.61	1342.83832
	7.2	20.416	4.6742	14.559	108.64	1316.46576
	7.8	20.443	4.6447	14.546	108.52	1309.63934
	9	20.43	4.6	14.5	not provided	1300

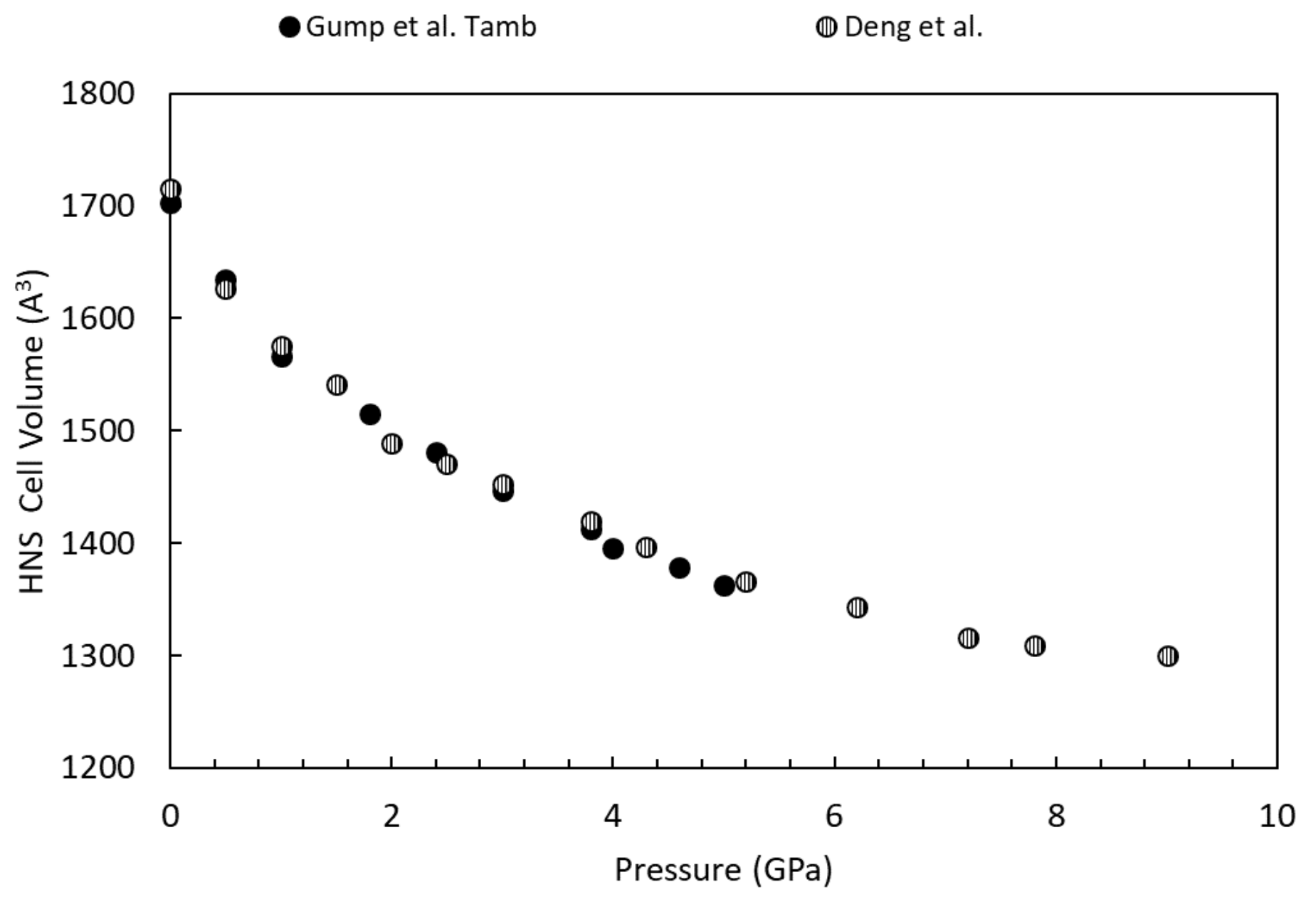

Figure S14 : Variation of Cell volume of trans-HNS with pressure[20,46]

10) TNP

Table S31 : Lattice parameters and cell volume of I-TNP in function of Pressure

Energetic materials	P (GPa)	a (Å)	b (Å)	c (Å)	β (°)	Volume (Å^3)
I-TNP Atceken et al.[47]	0.08	15.1685	8.2791	17.1995	92.258	2158.3
	0.24	15.1096	8.2388	17.1381	92.24	2131.8
	0.56	15.0127	8.1765	17.0501	92.217	2091.4
	0.94	14.8868	8.1009	16.9416	92.137	2041.7

	1.17	14.8192	8.0595	16.8916	92.106	2016.1
	1.31	14.7831	8.0363	16.8668	92.091	2002.5
	1.48	14.7374	8.0076	16.8368	92.073	1985.6
	1.59	14.6997	7.9827	16.8119	92.063	1971.5
	1.81	14.6509	7.9565	16.7874	92.006	1955.7
	1.99	14.6027	7.9293	16.7675	91.972	1940.3
	2.12	14.5646	7.9067	16.7509	91.944	1927.9
	2.31	14.5338	7.882	16.7371	91.969	1916.2
	2.53	14.5072	7.8613	16.7238	91.984	1906.1
	2.68	14.4765	7.8391	16.7086	91.996	1895.0
	2.85	14.4512	7.82	16.6974	92.009	1885.8
	3.02	14.4205	7.7985	14.6817	92.016	1650.1
	3.23	14.3879	7.7744	14.6731	92.022	1640.3
	3.34	14.3636	7.7548	16.6619	92.031	1854.8
	3.58	14.3312	7.7337	16.6455	92.024	1843.7
	3.77	13.3001	7.7112	16.6294	92.022	1704.4
	4.02	14.2726	7.6828	16.6126	92.11	1820.4
	4.37	14.2292	7.6577	16.5867	92.073	1806.2
II-TNP Atceken et al.[47]	5.3	19.5713	7.6509	14.6982	127.661	1742.3
	6.5	19.3994	7.5957	14.5289	127.637	1695.3
	7.3	19.319	7.5668	14.458	127.623	1674

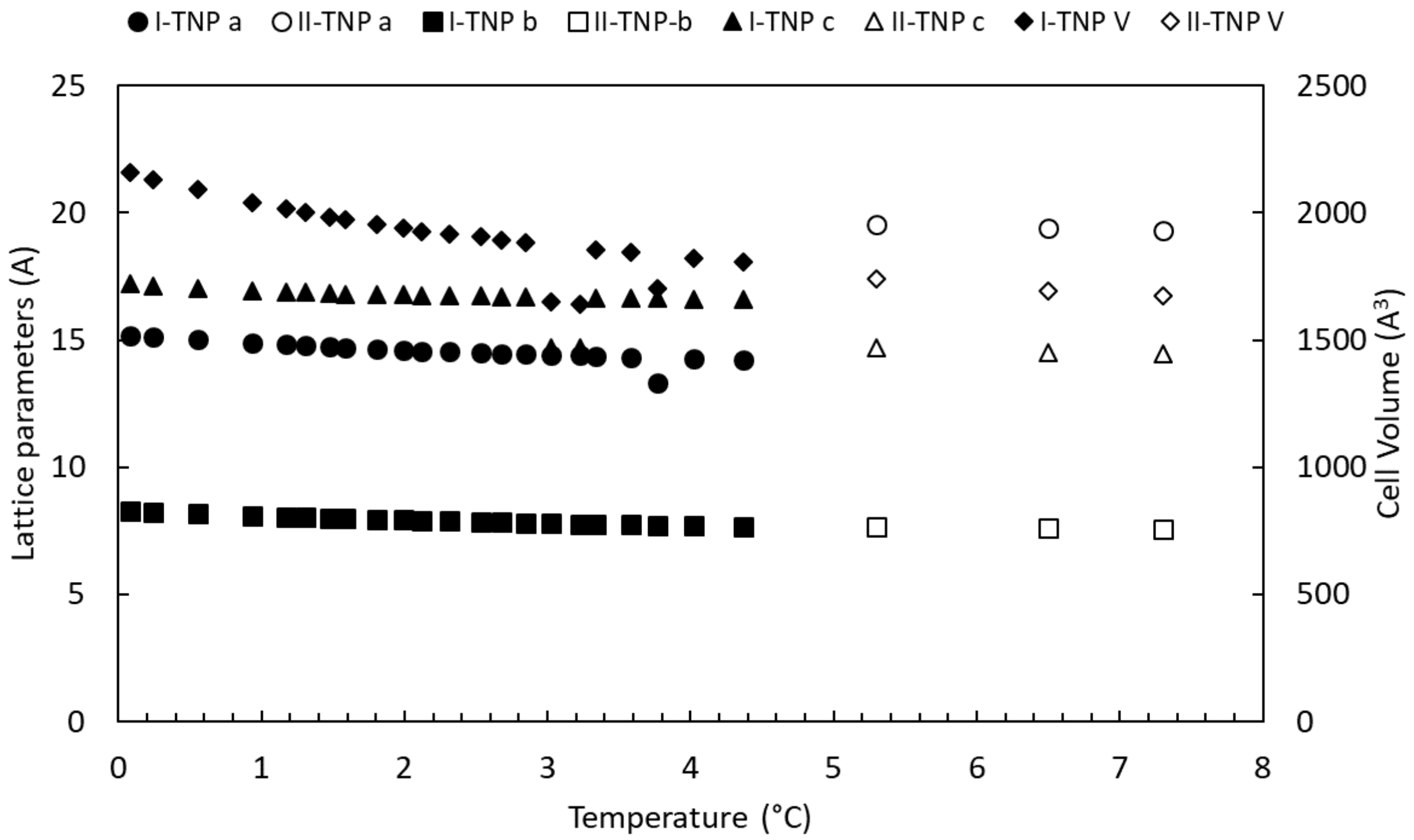

Figure S15 : Variation of lattice parameters and Cell volume of TNP with pressure[47]

11) DAAF

Table S32 : Lattice parameters and cell volume of DAAF in function of Pressure

Energetic materials	P (GPa)	a (Å)	b (Å)	c (Å)	β (°)	Volume (Å^3)

DAAF Chellappa et al.[48]	0	9.25	9.639	8.998	91.63	802.1
	1	9	9.5	8.75	91.63	747.82
	2	8.8	9.45	8.55	91.63	710.73
	3	8.75	9.4	8.45	91.7	694.70
	4	8.7	9.35	8.4	91.8	682.96
	5	8.55	9.3	8.3	92.4	659.39
	7	8.4	9.25	8.2	93.2	636.14
	8	8.38	9.25	8.1	93.2	626.89
	10	8.3	9.25	8	93.8	612.84
	12	8.25	9.2	7.9	94.4	597.84
	14	8.25	9.15	7.8	94.8	586.73
	16	8.25	9.1	7.8	95	583.35

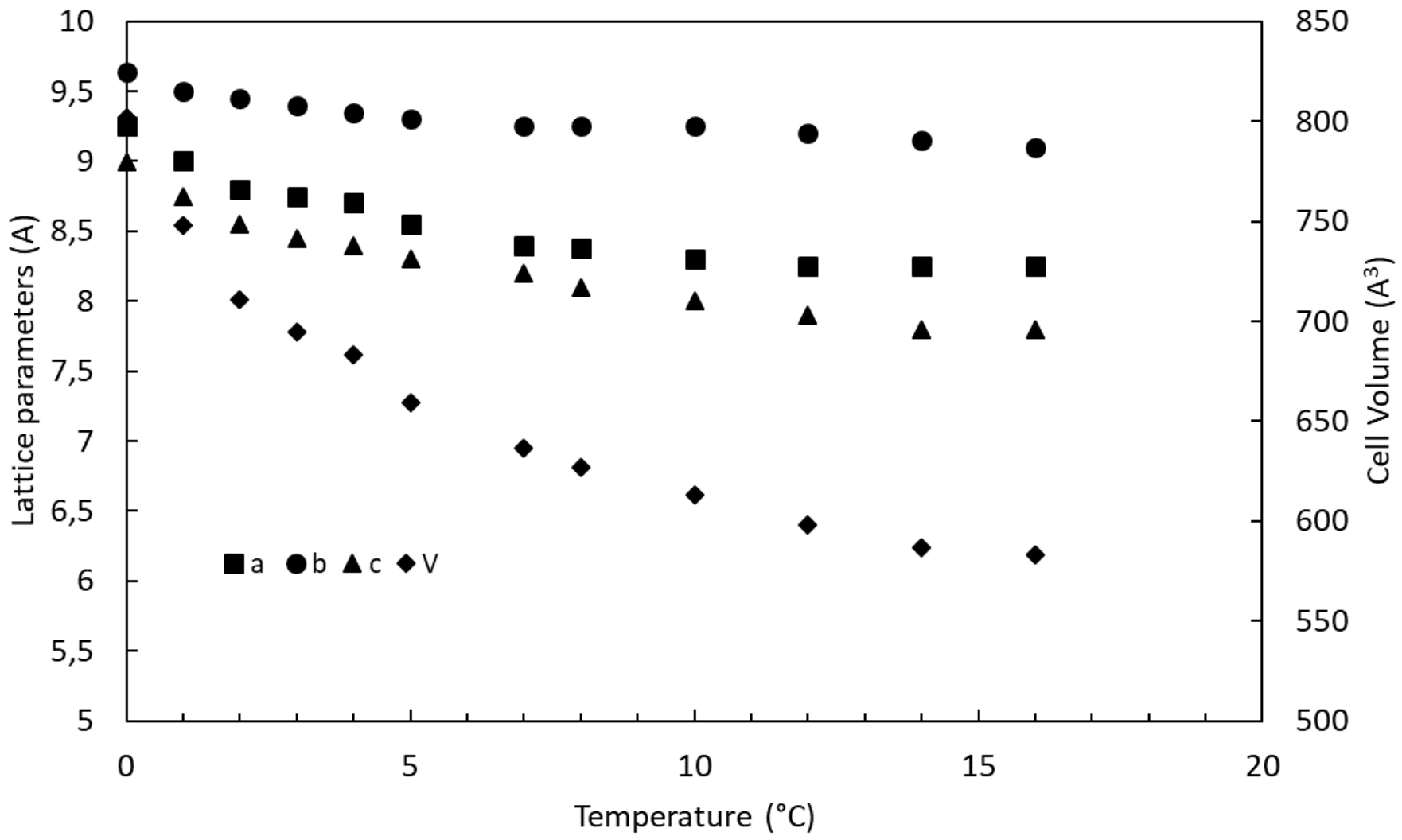

Figure S16 : Variation of lattice parameters and Cell volume of DAAF with pressure[48]

12) NTO

Table S33 : Lattice parameters and cell volume of α-NTO in function of Temperature

Energetic materials	T (°C)	a (Å)	b (Å)	c (Å)	α (°)	β (°)	γ (°)	Volume ($Å^3$)
α-NTO[1]	-173	5.1096	10.2161	17.758	106.721	97.905	90.108	878.50
	-148	5.1097	10.2204	17.7755	106.709	97.901	90.103	879.83
	-123	5.1116	10.2308	17.8049	106.699	97.888	90.110	882.59
	-98	5.1141	10.2419	17.8350	106.687	97.876	90.117	885.53
	-73	5.1160	10.2524	17.8665	106.675	97.861	90.122	888.42
	-48	5.1184	10.2642	17.9006	106.663	97.853	90.122	891.64
	-23	5.1275	10.2911	17.9593	106.653	97.846	90.121	989.57
	2	5.1296	10.3046	17.9936	106.643	97.830	90.126	901.92
	27	5.1247	10.3049	18.0024	106.629	97.822	90.116	901.62
α-NTO[21]	30	5.130	10.335	18.025	Not provided			906
	40	5.1305	10.340	18.035	Not provided			908
	50	5.1315	10.348	18.05	Not provided			910
	60	5.132	10.352	18.06	Not provided			911
	70	5.1325	10.355	18.07	Not provided			912
	80	5.1335	10.362	18.09	Not provided			914
	90	5.134	10.368	18.115	Not provided			915

	100	5.135	10.372	18.13	Not provided	916
	110	5.136	10.378	18.145	Not provided	917
	120	5.1365	10.385	18.16	Not provided	919
	130	5.1375	10.39	18.17	Not provided	921
	140	5.138	10.395	18.18	Not provided	922
	150	5.1385	10.40	18.19	Not provided	923

Table S34 : Lattice parameters and cell volume of β-NTO in function of Temperature

Energetic materials	T (°C)	a (Å)	b (Å)	c (Å)	β (°)	Volume ($Å^3$)
β-NTO[1]	-173	9.3129	5.4458	9.0261	101.464	448.64
	-123	9.3185	5.4615	9.0475	101.332	451.48
	-73	9.3220	5.4791	9.0685	101.182	454.39
	-23	9.3233	5.4977	9.0885	101.019	457.26
	-25	9.3207	5.5215	9.1102	100.870	460.44

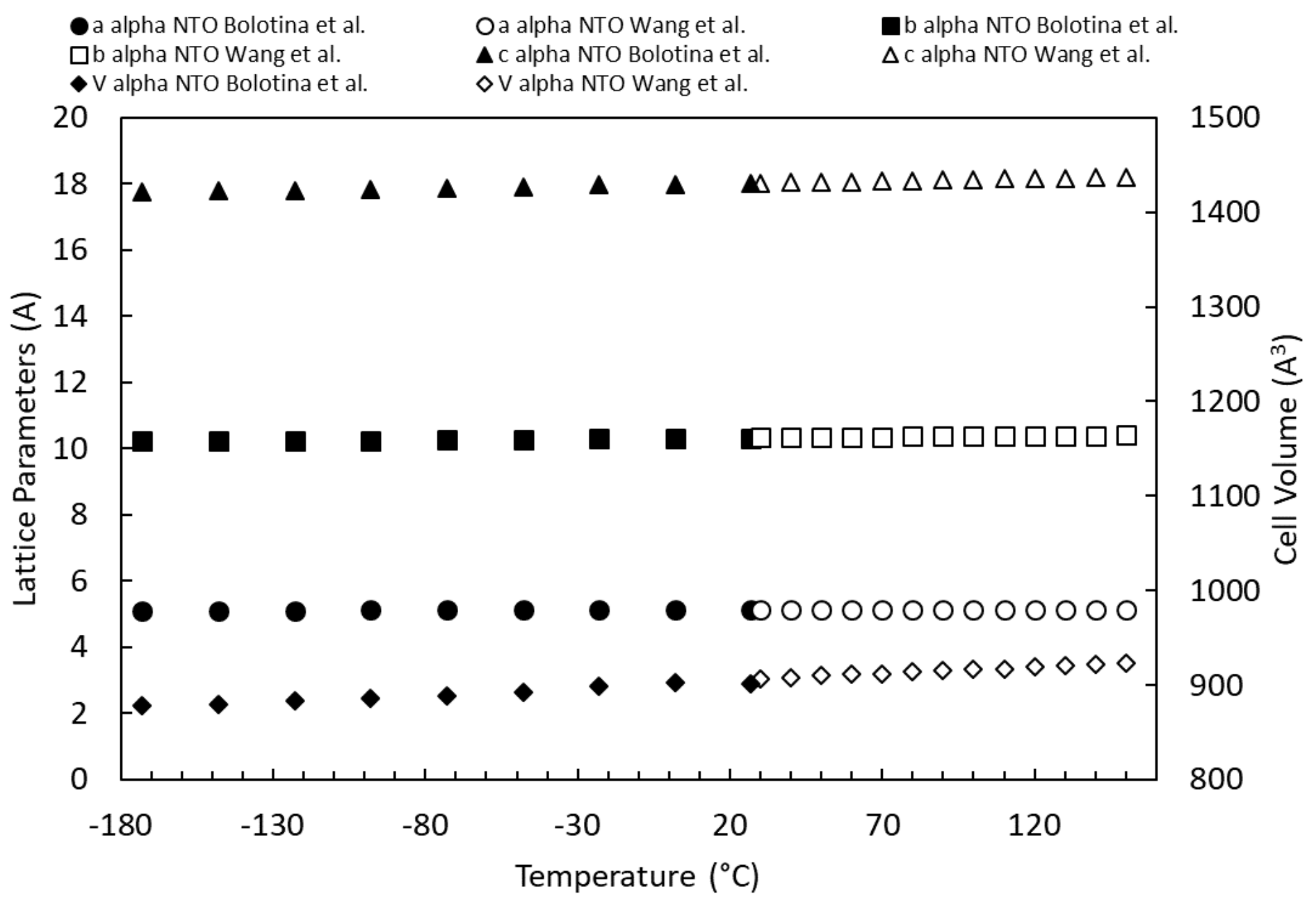

Figure S17 : Evolution of lattice parameters a. b and c and volume of α-NTO during the heating process[1,21]

Table S35 : Lattice parameters and cell volume of α-NTO in function of Pressure

Energetic materials	P (GPa)	a (Å)	b (Å)	c (Å)	β (°)	Volume ($Å^3$)
α-NTO Stavrou et al.[49]	0	not provided				900
	0.5	not provided				850
	1	not provided				800
	5	not provided				700
	8	not provided				660
	10	not provided				650
	13	not provided				625
	15	not provided				620
	20	not provided				600
	25	not provided				575

	30.8	not provided	530
	33	not provided	510

13) BTF

Table S36 : Lattice parameters and cell volume of ortho-BTF in function of Pressure

Energetic materials	T (°C)	a (Å)	b (Å)	c (Å)	Volume ($Å^3$)
Ortho-BTF[6]	-123	6.888385	19.340356	6.498446	865.747788
	-113	6.888385	19.340356	6.504964	866.616141
	-103	6.895308	19.359872	6.504964	868.362478
	-93	6.895308	19.36963	6.498446	867.929619
	-83	6.902231	19.389146	6.504964	870.548694
	-73	6.902231	19.398904	6.508223	871.423182
	-63	6.902231	19.408662	6.504964	871.424938
	-53	6.909154	19.437936	6.504964	873.61467
	-43	6.909154	19.437936	6.504964	873.61467
	-33	6.909154	19.457452	6.504964	874.491793
	-23	6.909154	19.457452	6.504964	874.491793
	-13	6.916077	19.476968	6.504964	876.246039
	-3	6.916077	19.496484	6.504964	877.124041
	7	6.923	19.516	6.511482	879.761567
	17	6.929923	19.535516	6.504964	880.639565
	27	6.936846	19.555032	6.504964	882.399964
	37	6.936846	19.574548	6.511482	884.165653
	47	6.936846	19.555032	6.518	884.1683
	57	6.9333845	19.574548	6.518	884.609062
	67	6.936846	19.594064	6.511482	885.047174
	77	6.9403075	19.633096	6.511482	887.252736
	87	6.9403075	19.633096	6.518	888.140877
	97	6.950692	19.642854	6.518	889.911849
	107	6.9541535	19.652612	6.518	890.797334
	117	6.957615	19.672128	6.511482	891.233657
	127	6.964538	19.71116	6.518	894.785323
	137	6.957615	19.730676	6.518	894.780919
	147	6.957615	19.779466	6.524518	897.890527
	157	6.971461	19.789224	6.524518	900.12122
	167	6.971461	19.847772	6.531036	903.686183
	177	6.978384	19.867288	6.531036	905.47305

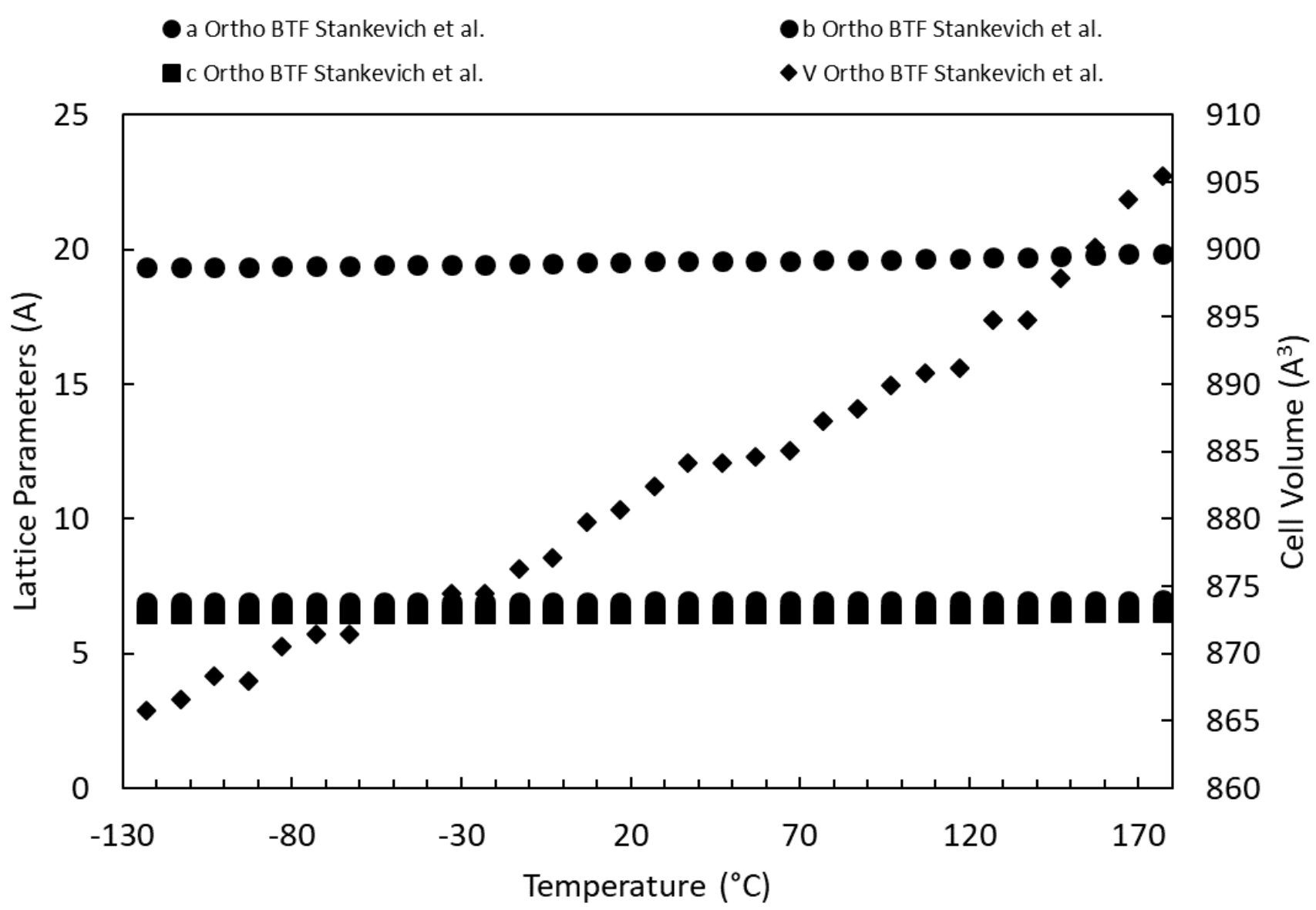

Figure S18 : Evolution of lattice parameters a. b and c and volume of ortho-BTF during the heating process[6]

14) DNAN

Table S37 : Lattice parameters and cell volume of I-DNAN in function of Temperature

Energetic materials	T (°C)	a (Å)	b (Å)	c (Å)	β (°)	Volume (Å^3)	V/Z (A^3)
I-DNAN[22]	-123	8.6870375	12.715	15.403595	82.83744	1688.19	211.024
	-113	8.691436	12.715	15.403595	82.79635	1688.19	211.024
	-103	8.700233	12.715	15.419076	82.5909	1689.91	211.239
	-93	8.717827	12.715	15.4268165	82.75526	1691.63	211.453
	-83	8.7222255	12.715	15.434557	82.5909	1695.06	211.882
	-73	8.726624	12.715	15.4422975	82.5909	1698.49	212.311
	-63	8.735421	12.715	15.4422975	82.50872	1701.92	212.740
	-53	8.744218	12.715	15.4469418	82.50872	1703.6	212.955
	-43	8.753015	12.715	15.450038	82.38545	1705.35	213.169
	-33	8.761812	12.715	15.450038	82.26218	1708.78	213.598
	-23	8.770609	12.715	15.481	82.18	1711.36	213.920
	-13	8.779406	12.715	15.481	82.18	1713.07	214.134
	-3	8.797	12.715	15.481	82.18	1715.65	214.456
	7	8.805797	12.715	15.481	82.18	1719.08	214.885
	17	8.814594	12.715	15.496481	82.09782	1719.93	214.992
	27	8.8277895	12.715	15.511962	82.01564	1723.37	215.421
	37	8.8453835	12.715	15.5197025	81.97455	1727.65	215.957
	47	8.858579	12.715	15.5197025	81.93346	1729.37	216.171
	57	8.867376	12.715	15.527443	81.7691	1732.80	216.600
	67	8.88497	12.715	15.542924	81.68692	1736.23	217.029
	77	8.902564	12.715	15.542924	81.56365	1739.66	217.458
	80	8.9069625	12.727715	15.558405	81.56365	1744.81	218.102

Table S38 : Lattice parameters and cell volume of II-DNAN in function of Temperature

Energetic materials	T (°C)	a (Å)	b (Å)	c (Å)	β (°)	Volume (A^3)	V/Z (A^3)
II-DNAN[22]	-123	3.85478	13.838194	15.426048	96.4282	818.84	204.711
	-113	3.862728	13.831323	15.426048	96.24626	820.53	205.134
	-103	3.870676	13.824452	15.426048	96.06432	821.383	205.345

	-93	3.878624	13.817581	15.418304	95.88238	823.07	205.768
	-83	3.886572	13.81071	15.418304	95.70044	823.92	205.980
	-73	3.89452	13.803839	15.41056	95.5185	824.76	206.191
	-63	3.902468	13.796968	15.41056	95.42753	826.45	206.614
	-53	3.910416	13.790097	15.41056	95.33656	828.99	207.249
	-43	3.918364	13.783226	15.41056	95.06365	830.68	207.672
	-33	3.926312	13.776355	15.426048	94.88171	832.38	208.095
	-23	3.93426	13.769484	15.441536	94.69977	834.07	208.518
	-13	3.938234	13.755742	15.472512	94.24492	837.45	209.363
	-3	3.942208	13.742	15.488	90.97	845.91	211.478
	7	3.974	13.742	15.472512	90.97	846.76	211.690
	17	3.981948	13.742	15.472512	90.87903	846.76	211.690
	27	3.985922	13.742	15.472512	90.78806	846.76	211.690
	37	3.989896	13.742	15.457024	90.51515	847.60	211.901
	47	3.99387	13.742	15.457024	90.24224	849.29	212.324
	57	3.997844	13.755742	15.457024	90.87903	850.99	212.747
	67	4.001818	13.769484	15.457024	89.60545	852.68	213.170
	77	4.009766	13.783226	15.472512	89.33254	853.52	213.382
	80	4.01374	13.796968	15.472512	89.1506	856.06	214.016
II-DNAN[23]	-173	3.8139	13.823	15.349	96.777	803.5	200.875
	-145	3.8278	13.813	15.364	96.512	807.1	201.775
	-100	3.8537	13.804	15.381	96.164	813.5	203.375
	-75	3.876	13.791	15.388	95.882	818.2	204.55
	-65	3.88	13.786	15.404	95.639	820	205
	-47	3.8968	13.781	15.419	95.382	824.4	206.1
	-37	3.908	13.78	15.42	95.31	826.8	206.7
	-12	3.9291	13.739	15.443	94.454	831.1	207.775
	0	3.957	13.735	15.454	91.156	839.7	209.925
	10	3.9618	13.745	15.47	91.089	842.3	210.575
	25	3.98	13.759	15.461	90.994	846.5	211.625

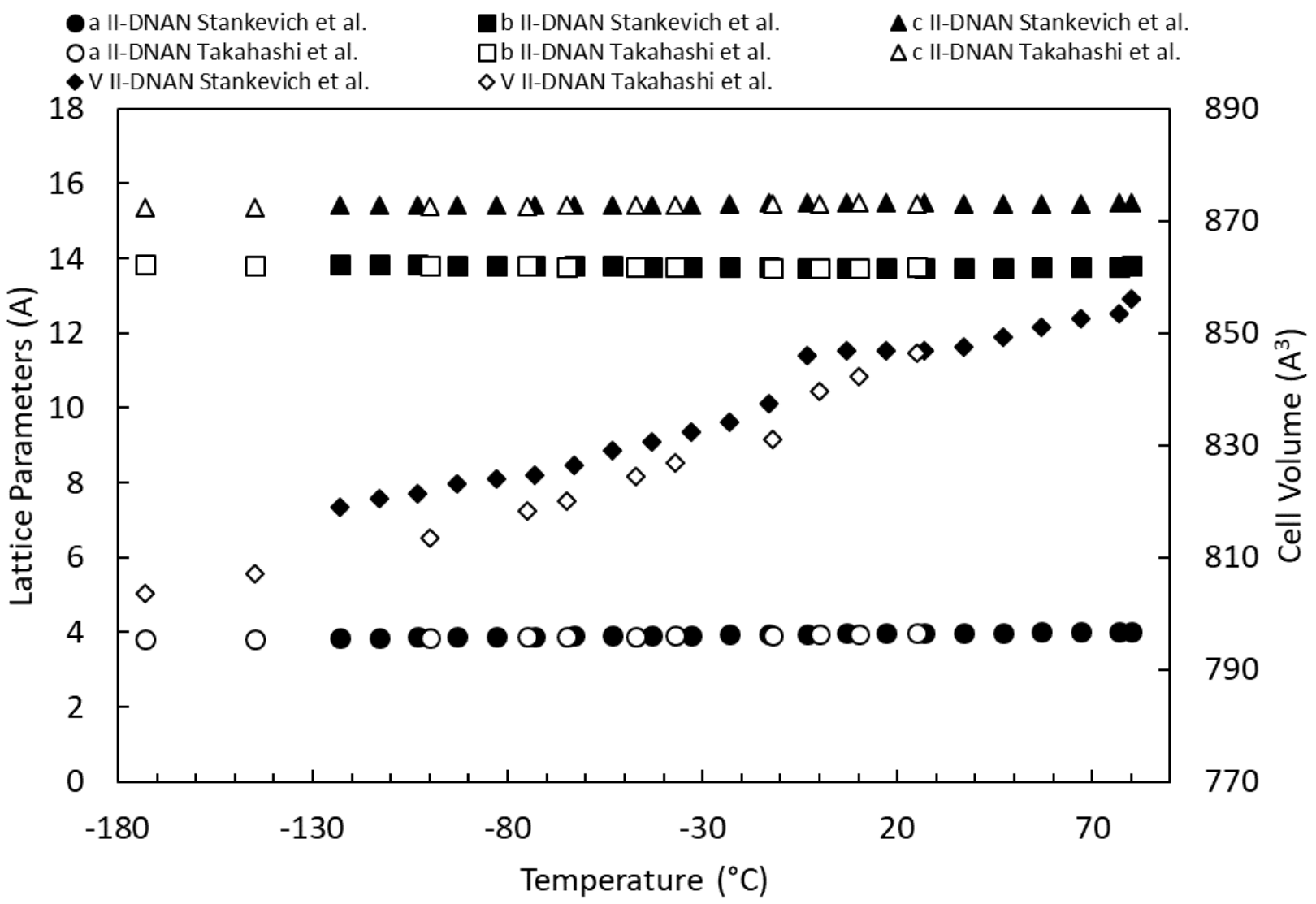

Figure S19 : Evolution of lattice parameters a. b and c and volume of II-DNAN during the heating process[22,23]

Table S39 : Lattice parameters and cell volume of I-DNAN and Ortho-DNAN in function of Pressure

Energetic materials	P (GPa)	a (Å)	b (Å)	c (Å)	β (°)	Volume (Å3)	V/Z (A^3)
I-DNAN Rajan et al.[50]	0	8.772	12.645	15.429	81.89	1694.3	211.67
	0.6	8.35	12.65	15.2	not provided	1625	203.12
	1	8.25	12.65	15.2	not provided	1600	200
	1.8	8.1	12.65	14.9	not provided	1550	193.75
	2.8	8.1	12.7	14.9	not provided	1550	193.75
	3	8.1	12.7	14.9	not provided	1550	193.75
Ortho-DNAN Rajan et al.[50]	3	7.8	12.25	14.5	90	1360	170
	6.8	7.9	11.6	14.25	90	1290	161.25
	8	7.9	11.6	14.2	90	1285	160.62
	9	8	11.5	14.15	90	1275	159.37

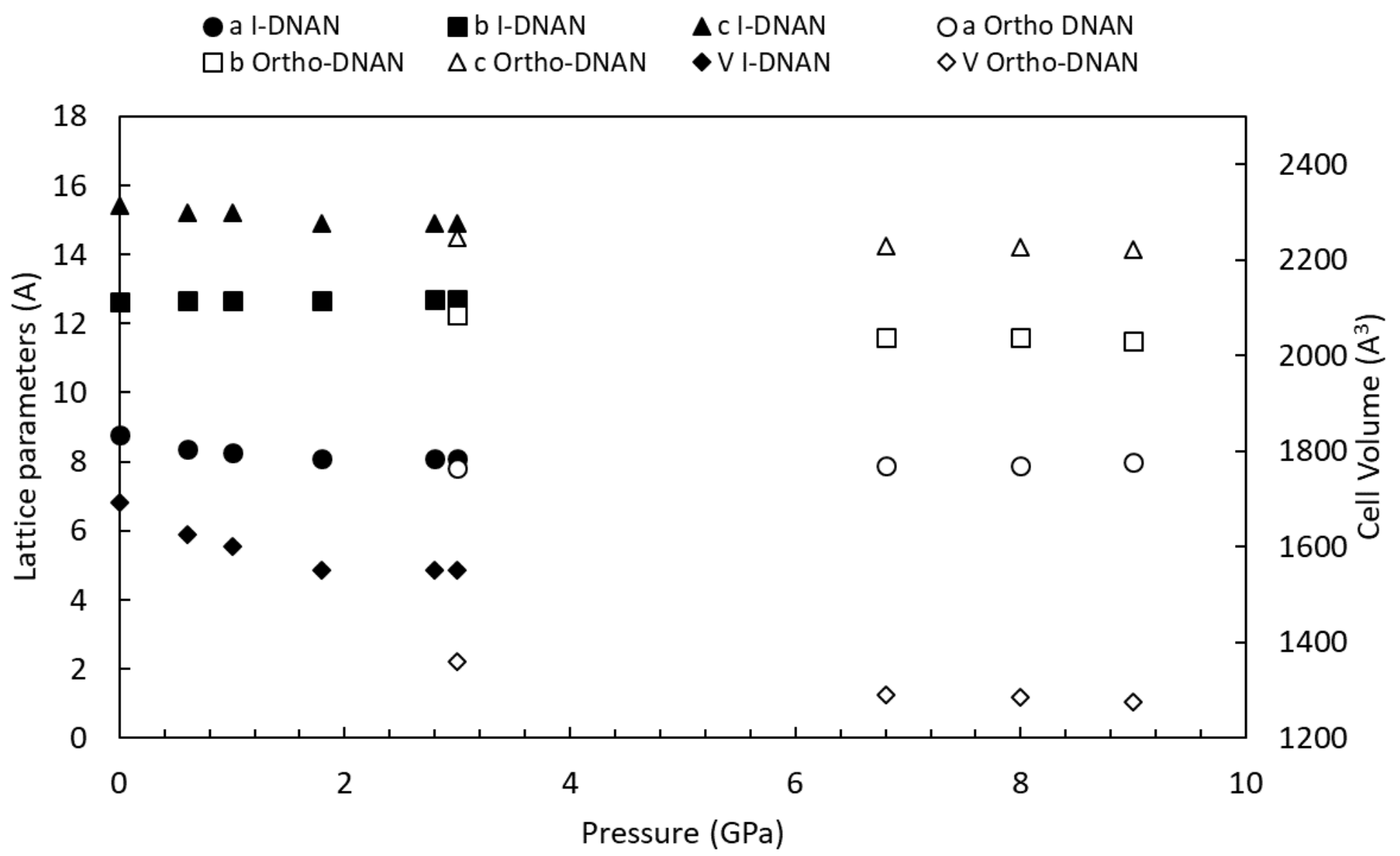

Figure S20 : Variation of lattice parameters and Cell volume of DNAN with pressure[50]

Table S40 : Temperature range. pressure range and CSD reference code of the energetic materials studied

Energetic Materials	Temperature range studied (°C)	Pressure range studied (GPa)	CSD reference code
RDX<br>$C_3H_6N_6O_6$	α-RDX : [-183 ; 27] [1]<br>α-RDX : [30°C ; 170][2]	α/γ-RDX [0;8][25]<br>ε-RDX : [0;5][24]<br>α-RDX : [0;5][24]	
TNT<br>$C_7H_5N_3O_6$	m-TNT :[-150 ;27][3]<br>o-TNT : [-150 ;27][3]	m-TNT :[0 ;10][27]<br>m-TNT :[0 ;26.5][28]	
HMX<br>$C_4H_8N_8O_8$	β-HMX : [-188 ;22][1]<br>β-HMX : [-150 ; 30][4]<br>β-HMX :[-100 ;160][5]	β-HMX :[0 ;42.6][29]<br>β-HMX : [0 ; 40][30]<br>β-HMX : [0 ; 14.5][31]	[4] : [OCHTET14-OCHTET21]<br>[5] : [OCHTET44-OCHTET56]
PETN<br>$C_5H_8N_4O_{12}$	I-PETN : [-263 ; 27][1]<br>I-PETN : [-123 ; 97][6]	I-PETN : [0 ; 10.45][32]	
FOX-7<br>$C_2H_4N_4O_4$	α/β-FOX-7 : [-73 ; 120][7]<br>α/β/γ-FOX-7 : [1 ;201][9]<br>α-FOX-7 : [27 ; 200][33]<br>γ-FOX-7 :[-173 ; 220][10]	α-FOX-7 : [0 ; 4.14][34]<br>α-FOX-7 : [0 ; 3.86][35]<br>α-FOX-7 : [0 ; 6.5][33]<br>α-Fox-7 : [0 ; 12.8][36]	[7] : [SEDTUQ02-SEDTUQ08]<br>[9] : [SEDTUQ15-SEDTUQ24]<br>[36] : [SEDTUQ33-SEDTUQ49]

CL-20<br>$C_6H_6N_{12}O_{12}$	ε-CL-20 : [25 ; 115][12]<br>ε-CL-20 : [-173 ; 25][1,11]<br>ε-CL-20 : [30 ; 130][13]<br>γ-CL-20 : [-173 ; 25][1,11]<br>γ-CL-20 : [30 ; 180] [13]<br>α-CL-20 : [30-120][13]<br>β-CL-20 : [30-120][13]	ε-CL-20 : [0 ; 12][37]<br>ε-CL-20 : [0 ; 5.6][27.28]	[11] : [PUBMUU07-PUBMUU16]<br>[37] : [PUBMUU28-PUBMUU44]
TATB<br>$C_6H_6N_6O_6$	TATB : [-59 ; 104][14]<br>TATB : [30 ; 240][15]	TATB : [0 ; 40][34.35]<br>TATB : [0 ; 16.5][41]<br>TATB : [0 ; 66][42]<br>TATB : [0 ; 13.2][40]	
LLM-105<br>$C_4H_4N_6O_5$	LLM -105 : [-268 ; 87][16]<br>LLM -105 : [25; 240][17]<br>LLM -105 : [30; 200][18]	LLM-105 : [0 ; 20][45]<br>LLM-105 : [0 ; 25] [16]<br>LLM-105 : [0 ; 5.4] [17]	
HNS<br>$C_{14}H_6N_6O_{12}$	*trans*-HNS : [30 ; 240][19]<br>*trans*-HNS : [30 ; 300][20]	*trans*-HNS : [0 ; 9][46]<br>*trans*-HNS : [0 ; 5.4][20]	[46] : [GIMBOT03-GIMBOT15]
TNP<br>$C_3HN_5O_6$	-	I/II-TNP : [0 ; 7.3][47]	[47] : [KAHHIM04-KAHHIM12]
DAAF<br>$C_4H_4N_8O_3$	-	DAAF : [0 ; 16][48]	
NTO<br>$C_2H_2N_4O_3$	α-NTO : [-173 ; 27][1]<br>α-NTO : [30 ; 150][21]<br>β-NTO : [-173 ; 25][1.50]	*α-NTO : [0 ; 33.3]*[49]	[51] : [QOYJOD01-QOYJOD05]
DATB<br>$C_6H_5N_5O_6$	-	-	
TNB<br>$C_6H_3N_3O_6$	-	-	
BTF<br>$C_6N_6O_6$	Ortho-BTF : [-123 :177][6]	-	
DNAN<br>$C_7H_6N_2O_5$	I-DNAN : [-123 ; 80][22]<br>II-DNAN : [-173 ; 80][22]<br>II-DNAN : [-173 ; 25][23]	I-DNAN : [0 ; 9][50]	[23] : [FESNEW03-FESNEW21]

References

1. Bolotina, N. B. & Pinkerton, A. A. Temperature dependence of thermal expansion tensors of energetic materials. *J. Appl. Crystallogr.* **48**, 1364–1380 (2015).
2. Sun, J. *et al.* Investigation on the Thermal Expansion and Theoretical Density of 1,3,5-Trinitro-1,3,5-Triazacyclohexane. *Propellants, Explos. Pyrotech.* **36**, 341–346 (2011).
3. Vrcelj, R. M., Sherwood, J. N., Kennedy, A. R., Gallagher, H. G. & Gelbrich, T. Polymorphism in 2-4-6 Trinitrotoluene. *Cryst. Growth Des.* **3**, 1027–1032 (2003).
4. Deschamps, J. R., Frisch, M. & Parrish, D. Thermal Expansion of HMX. *J. Chem. Crystallogr.* **41**, 966–970 (2011).
5. Yan, M. *et al.* Thermal Expansion, Atomic Vibration, and Molecular Conformation of β-HMX at a Wide Temperature Range Prior to Phase Transformation. *J. Phys. Chem. C* **127**, 20838–20848 (2023).

6. Stankevich, A. V, Loboiko, B. G., Garmashev, A. Y., Kostitsyn, O. V & Taibinov, N. P. Thermal strain tensors for benzotrifuroxane and pentaerythritol tetranitrate molecular crystals. *J. Phys. Conf. Ser.* **1147**, 12009 (2019).
7. Evers, J., Klapötke, T. M., Mayer, P., Oehlinger, G. & Welch, J. α- and β-FOX-7, Polymorphs of a High Energy Density Material, Studied by X-ray Single Crystal and Powder Investigations in the Temperature Range from 200 to 423 K. *Inorg. Chem.* **45**, 4996–5007 (2006).
8. Aree, T., McMonagle, C. J., Michalchuk, A. A. L. & Chernyshov, D. Low-frequency lattice vibrations from atomic displacement parameters of {$α$}-FOX-7, a high energy density material. *Acta Crystallogr. Sect. B* **78**, 376–384 (2022).
9. McMonagle, C. J., Michalchuk, A. A. L. & Chernyshov, D. FOX-7 high-energy-density material: thermal expansion and phase transitions revisited. *Acta Crystallogr. Sect. B* **78**, 91–95 (2022).
10. Crawford, M.-J. *et al.* γ-FOX-7: Structure of a High Energy Density Material Immediately Prior to Decomposition. *Propellants, Explos. Pyrotech.* **32**, 478–495 (2007).
11. Bolotina, N. B., Hardie, M. J., Speer Jr, R. L. & Pinkerton, A. A. Energetic materials: variable-temperature crystal structures of γ- and ε-HNIW polymorphs. *J. Appl. Crystallogr.* **37**, 808–814 (2004).
12. Liang, W. *et al.* Thermally induced polymorphic transformation of Hexanitrohexaazaisowurtzitane (CL-20). *Powder Technol.* **395**, 732–742 (2022).
13. Pu, L., Xu, J.-J., Liu, X.-F. & Sun, J. Investigation on the Thermal Expansion of Four Polymorphs of Crystalline CL-20. *J. Energ. Mater.* **34**, 205–215 (2016).
14. Kolb, J. R. & Rizzo, H. F. Growth of 1,3,5-Triamino-2,4,6-trinitrobenzene (TATB) I. Anisotropic thermal expansion. *Propellants, Explos. Pyrotech.* **4**, 10–16 (1979).
15. Sun, J. *et al.* Crystal State of 1,3,5-Triamino-2,4,6-Trinitrobenzene (TATB) Undergoing Thermal Cycling Process. *J. Energ. Mater.* **28**, 189–201 (2010).
16. Xu, Z. *et al.* Pressure- and Temperature-Dependent Structural Stability of LLM-105 Crystal. *J. Phys. Chem. C* **123**, 1110–1119 (2019).
17. Gump, J. C. *et al.* Equations of state of 2,6-diamino-3,5-dinitropyrazine-1-oxide. *J. Appl. Phys.* **110**, 73523 (2011).
18. Li, J. *et al.* The Temperature-Dependent Thermal Expansion of 2,6-Diamino-3,5-dinitropyrazine-1-oxide Effected by Hydrogen Bond Network Relaxation. *J. Energ. Mater.* **34**, 170–182 (2016).
19. Shu, X. *et al.* Thermal expansion and theoretical density of 2,2′,4,4′,6,6′-hexanitrostilbene. *J. Mater. Sci.* **46**, 2536–2540 (2011).
20. Gump, J. C., Stoltz, C., Mason, B. P. & Heim, E. M. Equations of state of hexanitrostilbene (HNS). *AIP Conf. Proc.* **1426**, 575–578 (2012).
21. Wang, Z. *et al.* Thermal expansion characteristics of high energy insensitive explosive α-NTO. *J. Energ. Mater.* **43**, 444–459 (2025).
22. Stankevich, A. V *et al.* Tensors of thermal deformation for various polymorphic modifications of 2,4-dinitroanisole. *Energ. Mater. Front.* **5**, 257–266 (2024).
23. Takahashi, H. & Tamura, R. Low temperature phase transition induced biaxial negative thermal expansion of 2{,}4-dinitroanisole. *CrystEngComm* **17**, 8888–8896 (2015).
24. Millar, D. I. A. *et al.* Pressure-cooking of explosives—the crystal structure of ε-RDX as determined by X-ray and neutron diffraction. *Chem. Commun.* **46**, 5662–5664 (2010).
25. Oswald, I. D. H. *et al.* High-pressure structural studies of energetic compounds. *High Press. Res.* **30**, 280–291 (2010).
26. Millar, D. I. A. Energetic Materials at Extreme Conditions. (2010).
27. Bowden, P. R. *et al.* The high-pressure phase stability of 2,4,6-trinitrotoluene ({TNT}). *J. Phys. Conf. Ser.* **500**, 52006 (2014).
28. Stevens, L. L., Velisavljevic, N., Hooks, D. E. & Dattelbaum, D. M. The high-pressure phase behavior and compressibility of 2,4,6-trinitrotoluene. *Appl. Phys. Lett.* **93**, 81912 (2008).
29. Yoo, C.-S. & Cynn, H. Equation of state, phase transition, decomposition of β-HMX (octahydro-1,3,5,7-tetranitro-1,3,5,7-tetrazocine) at high pressures. *J. Chem. Phys.* **111**, 10229–10235 (1999).
30. Sui, Z. *et al.* Phase Confirmation and Equation of State of β-HMX under 40 GPa. *J. Phys.*

Chem. C **123**, 30121–30128 (2019).
31. Gao, D. *et al.* Phase transitions and chemical reactions of octahydro-1{,}3{,}5{,}7-tetranitro-1{,}3{,}5{,}7-tetrazocine under high pressure and high temperature. *RSC Adv.* **9**, 5825–5833 (2019).
32. Olinger, B., Halleck, P. M. & Cady, H. H. The isothermal linear and volume compression of pentaerythritol tetranitrate (PETN) to 10 GPa (100 kbar) and the calculated shock compression. *J. Chem. Phys.* **62**, 4480–4483 (1975).
33. Zhang, J., Velisavljevic, N., Zhu, J. & Wang, L. Equation of state and thermodynamic Grüneisen parameter of monoclinic 1,1-diamino-2,2-dinitroethylene. *J. Phys. Condens. Matter* **28**, 395402 (2016).
34. Hunter, S. *et al.* High-Pressure Experimental and DFT-D Structural Studies of the Energetic Material FOX-7. *J. Phys. Chem. C* **119**, 2322–2334 (2015).
35. Peiris, S. M., Wong, C. P. & Zerilli, F. J. Equation of state and structural changes in diaminodinitroethylene under compression. *J. Chem. Phys.* **120**, 8060–8066 (2004).
36. Dreger, Z. A. *et al.* High-Pressure Crystal Structures of an Insensitive Energetic Crystal: 1,1-Diamino-2,2-dinitroethene. *J. Phys. Chem. C* **120**, 1218–1224 (2016).
37. Konar, S. *et al.* High-Pressure Neutron Powder Diffraction Study of ε-CL-20: A Gentler Way to Study Energetic Materials. *J. Phys. Chem. C* **124**, 27985–27995 (2020).
38. Gump, J. C. & Peiris, S. M. Phase transitions and isothermal equations of state of epsilon hexanitrohexaazaisowurtzitane (CL-20). *J. Appl. Phys.* **104**, 83509 (2008).
39. Gump, J. C., Stoltz, C. A. & Peiris, S. M. PHASE STABILITY OF EPSILON AND GAMMA HNIW (CL-20) AT HIGH-PRESSURE AND TEMPERATURE. *AIP Conf. Proc.* **955**, 127–132 (2007).
40. Stevens, L. L., Velisavljevic, N., Hooks, D. E. & Dattelbaum, D. M. Hydrostatic Compression Curve for Triamino-Trinitrobenzene Determined to 13.0 GPa with Powder X-Ray Diffraction. *Propellants, Explos. Pyrotech.* **33**, 286–295 (2008).
41. Sun, X. *et al.* Pressure-Induced Conformer Modifications and Electronic Structural Changes in 1,3,5-Triamino-2,4,6-trinitrobenzene (TATB) up to 20 GPa. *J. Phys. Chem. C* **122**, 15861–15867 (2018).
42. Plisson, T. *et al.* Equation of state of 1,3,5-triamino-2,4,6-trinitrobenzene up to 66 GPa. *J. Appl. Phys.* **122**, 235901 (2017).
43. Steele, B. A. *et al.* Pressure-induced phase transition in 1,3,5-triamino-2,4,6-trinitrobenzene (TATB). *Appl. Phys. Lett.* **114**, 191901 (2019).
44. Steele, B. A., Stavrou, E., Prakapenka, V. B., Kroonblawd, M. P. & Kuo, I.-F. W. High-Pressure Equation of State of 1,3,5-triamino-2,4,6-trinitrobenzene: Insights into the Monoclinic Phase Transition, Hydrogen Bonding, and Anharmonicity. *J. Phys. Chem. A* **124**, 10580–10591 (2020).
45. Stavrou, E. *et al.* The high pressure structure and equation of state of 2,6-diamino-3,5-dinitropyrazine-1-oxide (LLM-105) up to 20 GPa: X-ray diffraction measurements and first principles molecular dynamics simulations. *J. Chem. Phys.* **143**, 144506 (2015).
46. Deng, X. *et al.* Negative linear compressibility and structural stability of the energetic material trans-hexanitrostilbene under pressure. *Phys. Chem. Chem. Phys.* **27**, 3375–3383 (2025).
47. Atceken, N. *et al.* High-pressure structural studies and pressure-induced sensitisation of 3{,}4{,}5-trinitro-1H-pyrazole. *Phys. Chem. Chem. Phys.* **25**, 31646–31654 (2023).
48. Chellappa, R. S. *et al.* Intermolecular Stabilization of 3,3′-Diamino-4,4′-azoxyfurazan (DAAF) Compressed to 20 GPa. *J. Phys. Chem. A* **118**, 5969–5982 (2014).
49. Stavrou, E., Zaug, J. M., Bastea, S. & Crowhurst, J. C. The equation of state of 5-nitro-2,4-dihydro-1,2,4,-triazol-3-one determined via in-situ optical microscopy and interferometry measurements. *J. Appl. Phys.* **119**, 135904 (2016).
50. Rajan, R. *et al.* Pressure dependent phase transformations of energetic material 2,4−dinitroanisole using Raman spectroscopy, X-ray diffraction and first principles calculations. *J. Mol. Struct.* **1247**, 131356 (2022).
51. Bolotina, N. B., Zhurova, E. & Pinkerton, A. A. Energetic materials: variable-temperature crystal structure of β-NTO. *J. Appl. Crystallogr.* **36**, 280–285 (2003).
52. Karpowicz, R. J., Sergio, S. T. & Brill, T. B. .beta.-Polymorph of hexahydro-1,3,5-trinitro-s-

triazine. A Fourier transform infrared spectroscopy study of an energetic material. *Ind. Eng. Chem. Prod. Res. Dev.* **22**, 363–365 (1983).

53. Cady, H. H., Larson, A. C. & Cromer, D. T. The crystal structure of {$α$}-HMX and a refinement of the structure of {$β$}-HMX. *Acta Crystallogr.* **16**, 617–623 (1963).
54. Achuthan, C. P. & Jose, C. I. Studies on octahydro-1,3,5,7-tetranitro-1,3,5,7-tetrazocine (HMX) polymorphism. *Propellants, Explos. Pyrotech.* **15**, 271–275 (1990).
55. Nielsen, A. T. *et al.* Synthesis of polyazapolycyclic caged polynitramines. *Tetrahedron* **54**, 11793–11812 (1998).
56. Foltz, M. F., Coon, C. L., Garcia, F. & Nichols III, A. L. The thermal stability of the polymorphs of hexanitrohexaazaisowurtzitane, part II. *Propellants, Explos. Pyrotech.* **19**, 133–144 (1994).
57. Wei, X., Xu, J., Li, H., Long, X. & Zhang, C. Comparative Study of Experiments and Calculations on the Polymorphisms of 2,4,6,8,10,12-Hexanitro-2,4,6,8,10,12-hexaazaisowurtzitane (CL-20) Precipitated by Solvent/Antisolvent Method. *J. Phys. Chem. C* **120**, 5042–5051 (2016).
58. Hervé, G., Roussel, C. & Graindorge, H. Selective Preparation of 3,4,5-Trinitro-1H-Pyrazole: A Stable All-Carbon-Nitrated Arene. *Angew. Chemie Int. Ed.* **49**, 3177–3181 (2010).
59. Nelyubina, Y. V, Dalinger, I. L. & Lyssenko, K. A. Pseudosymmetry in Trinitropyrazole: The Cost of Error in Space-Group Determination. *Angew. Chemie Int. Ed.* **50**, 2892–2894 (2011).
60. Beal, R. W., Incarvito, C. D., Rhatigan, B. J., Rheingold, A. L. & Brill, T. B. X-Ray Crystal Structures of Five Nitrogen-Bridged Bifurazan Compounds. *Propellants, Explos. Pyrotech.* **25**, 277–283 (2000).
61. Holden, J. R. The structure of 1,3-diamino-2,4,6-trinitrobenzene, form I. *Acta Crystallogr.* **22**, 545–550 (1967).
62. Kohno, Y. *et al.* Molecular Dynamics Studies of the Structural Change in 1,3-Diamino-2,4,6-trinitrobenzene (DATB) in the Crystalline State under High Pressure. *J. Phys. Chem. A* **113**, 2551–2560 (2009).
63. Ciezak, J. A. & Jenkins, T. A. The Low-Temperature High-Pressure Phase Diagram of Energetic Materials: I. Hexahydro-1,3,5-Trinitro-s-Triazine. *Propellants, Explos. Pyrotech.* **33**, 390–395 (2008).
64. Davidson, A. J. *et al.* Explosives under pressure—the crystal structure of γ-RDX as determined by high-pressure X-ray and neutron diffraction. *CrystEngComm* **10**, 162–165 (2008).
65. Ciezak, J. A., Jenkins, T. A., Liu, Z. & Hemley, R. J. High-Pressure Vibrational Spectroscopy of Energetic Materials: Hexahydro-1,3,5-trinitro-1,3,5-triazine. *J. Phys. Chem. A* **111**, 59–63 (2007).
66. Hu, J., Bi, J., Zhou, M., Sun, C. & Men, Z. Phase transition of HMX and RDX by low wavenumber Raman vibrations under high pressure. *J. Mol. Liq.* **336**, 116177 (2021).
67. Dreger, Z. A. & Gupta, Y. M. High Pressure–High Temperature Polymorphism and Decomposition of Pentaerythritol Tetranitrate (PETN). *J. Phys. Chem. A* **117**, 5306–5313 (2013).
68. Russell, T. P., Miller, P. J., Piermarini, G. J. & Block, S. Pressure/temperature phase diagram of hexanitrohexaazaisowurtzitane. *J. Phys. Chem.* **97**, 1993–1997 (1993).
69. Millar, D. I. A. *et al.* Putting the squeeze on energetic materials—structural characterisation of a high-pressure phase of CL-20. *CrystEngComm* **12**, 2524–2527 (2010).
70. Olinger, B. W. & Cady, H. H. Hydrostatic compression of explosives and detonation products to 10 GPa (100 kbars) and their calculated shock compression: results for PETN, TATB, CO/sub 2/, and H/sub 2/O. in (1976).
71. Wang, W.-P. *et al.* The structural response to pressure of energetic crystal 1,3-Diamino-2,4,6-trinitrobenzene: Density functional theory calculations and Hirshfeld surfaces analysis. *Comput. Theor. Chem.* **1091**, 57–63 (2016).
72. Deng, Z., Wang, Y., Qi, G. & Zhang, Q. High-pressure structural stability and melting performance of α-2,4-dinitroanisole. *Energ. Mater. Front.* **2**, 272–277 (2021).
